# Supplementary material for: Cellular model system to dissect the isoform-selectivity of Akt inhibitors
Source: Nat Commun. 2021 Sep 6;12:5297. doi: 10.1038/s41467-021-25512-8 (PMC8421423; doi:10.1038/s41467-021-25512-8)
Supplement: Supplementary file 1 — Supplementary Information [file 41467_2021_25512_MOESM1_ESM.pdf]

Supplementary Information

Cellular model system to dissect isoform-selectivity of Akt inhibitors

Lena Quambusch[‡1], Laura Depta[‡1], Ina Landel[1], Melissa Lubeck[1], Tonia Kirschner[1], Jonas Nabert[1], Niklas Uhlenbrock[1], Jörn Weisner[1], Michael Kostka[2], Laura M. Levy[2], Carsten Schultz-Fademrecht[3], Franziska Glanemann[4,5], Kristina Althoff[4,5], Matthias P. Müller[1], Jens T. Siveke[4,5] and Daniel Rauh[1]*.

[1]Faculty of Chemistry and Chemical Biology, TU Dortmund University and Drug Discovery Hub Dortmund (DDHD), Zentrum für Integrierte Wirkstoffforschung (ZIW), Otto-Hahn-Strasse 4a, 44227 Dortmund, Germany

[2]Medicinal Chemistry, Taros Chemicals GmbH & Co. KG, Emil-Figge-Strasse 76a, 44227, Dortmund, Germany

[3]Lead Discovery Center GmbH, Otto-Hahn-Strasse 15, 44227 Dortmund, Germany

[4]Bridge Institute of Experimental Tumor Therapy, West German Cancer Center, University Medicine Essen, Essen, Germany

[5]Division of Solid Tumor Translational Oncology, German Cancer Research Center (DKFZ) and German Cancer Consortium (DKTK), partner site Essen, Heidelberg, Germany

*E-mail, daniel.rauh@tu-dortmund.de.

‡These authors contributed equally: Lena Quambusch, Laura Depta

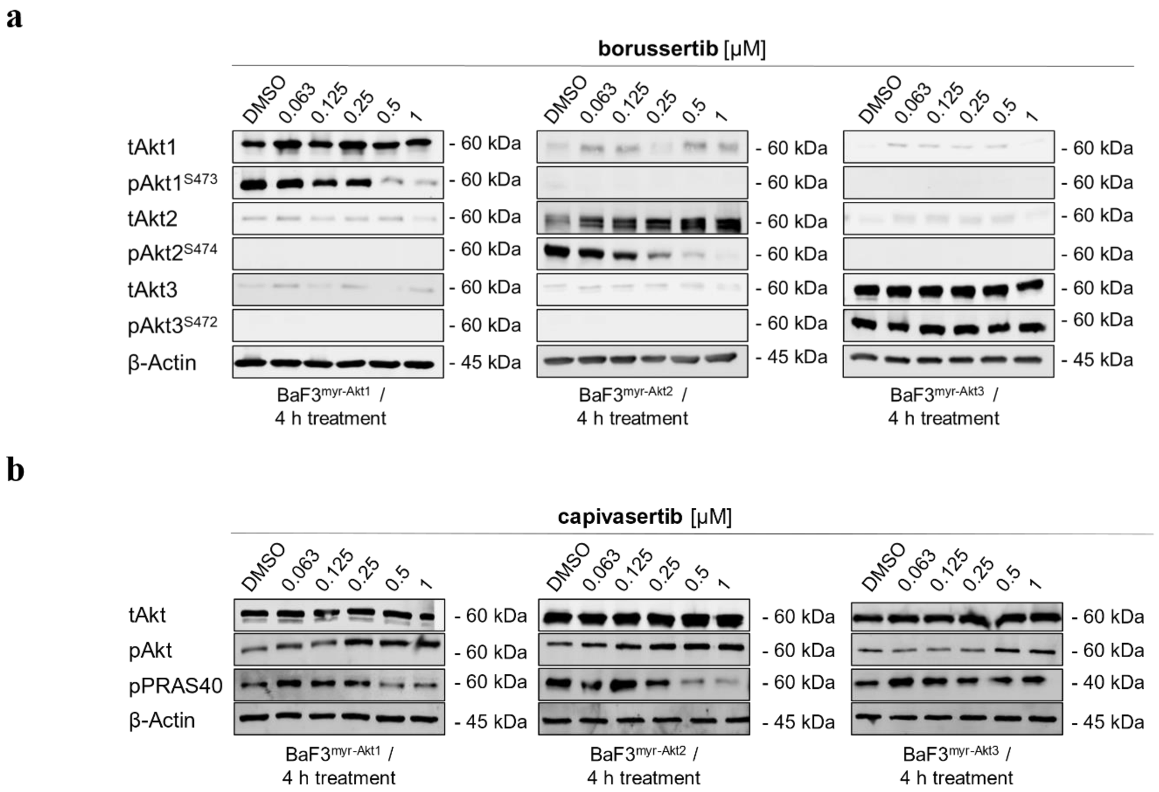

Supplementary Figure 1. Ba/F3 myr-Akt isoform dependent cell lines with literature known Akt inhibitors. (a) Immunoblots of Ba/F3 myr-Akt isoform dependent cell lines treated with borussertib using Akt isoform specific antibodies for comparison of expression levels and activity of the individual Akt isoform (n = 1 biologically independent experiment). **(b)** Immunoblots of Ba/F3 myr-Akt isoform dependent cell lines treated with ATP-competitive inhibitor capivasertib (n = 1 biologically independent experiment). Source Data are provided with this paper.

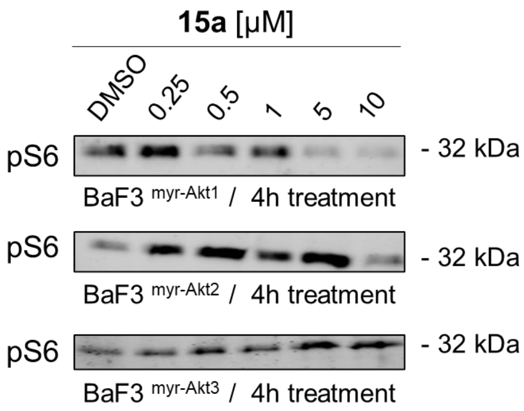

Supplementary Figure 2. BaF3 myr-Akt isoform dependent cell lines treated with Akt inhibitor 15a. Immunoblot of BaF3 myr-Akt isoforms dependent cell lines treated with molecule **15a** for the visualization of isoform specific induced dose-dependent downregulation of pS6 (n = 1 biologically independent experiment). Source Data are provided with this paper.

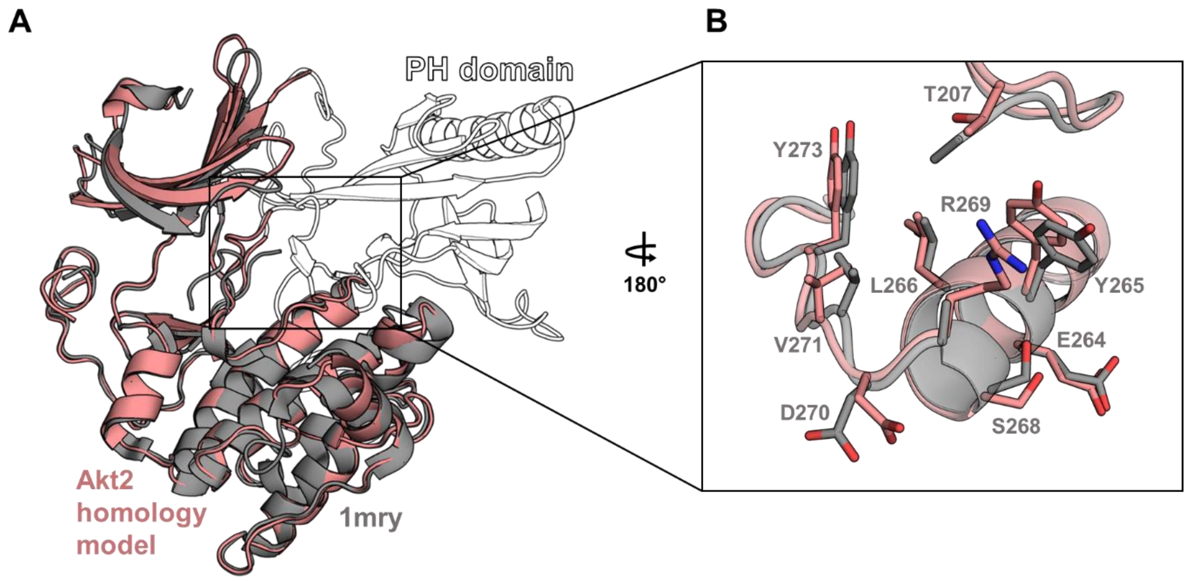

Supplementary Figure 3. Comparison of the Akt2 homology model with known crystal structure of Akt2 kinase domain. (A) Depiction of an overall alignment of the Akt2 homology model kinase domain (rose) with a known crystal structure of inactive Akt2 kinase domain (grey, PDB: 1MRY).[1]; the position of the PH domain of the Akt2 homology model is shown in white (Alignment generated with PyMOL). (B) Comparison of the c-terminal end of αE-helix and parts of the catalytic loop (grey: Akt2 kinase domain, PDB: 1MRY; rose: Akt2 homology model). The amino acids and secondary structure elements are in good agreement between both models. The side chain of Arg269 is not included in the model of the kinase domain (PDB: 1MRY).[1]

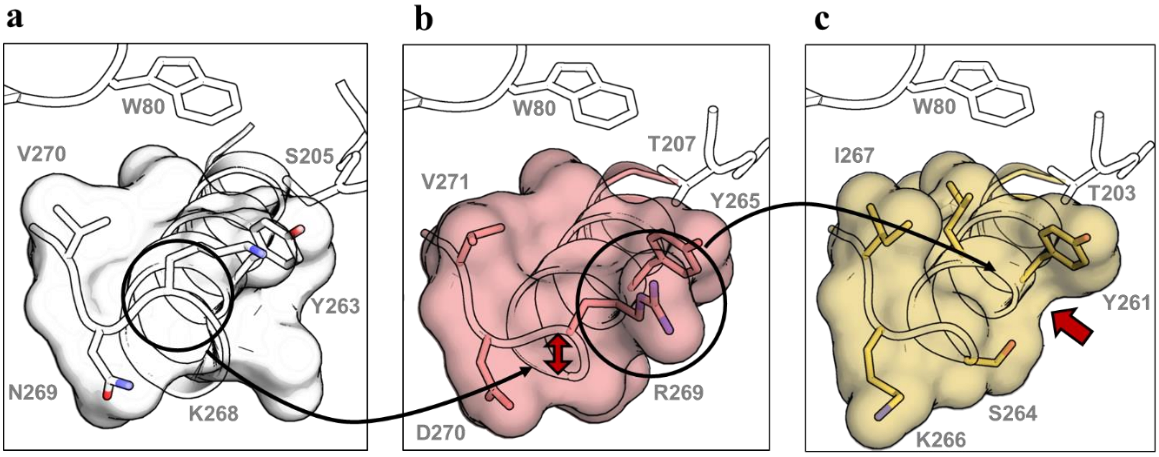

Supplementary Figure 4: Homology models reveal possible structural differences within the Akt isoforms allosteric binding site. View of the interdomain binding site in (a) Akt1 (white; pdb 6S9X)[2], (b) Akt2 (red), and (c) Akt3 (yellow) homology models. The structural changes within the allosteric site and the different side chain residues of each isoform are highlighted. Compared Akt1 to Akt2, the model shows a more narrow and slightly less helical c-terminal end of the αE-helix. Hence, the basic side chain of Arg269 in Akt2 is shifted in a slightly different position than the equivalent amino acid (K268) in Akt1. In contrast, the model of Akt3 highlights a more accessible surface at this before mentioned position, due to a glycine residue (G265).

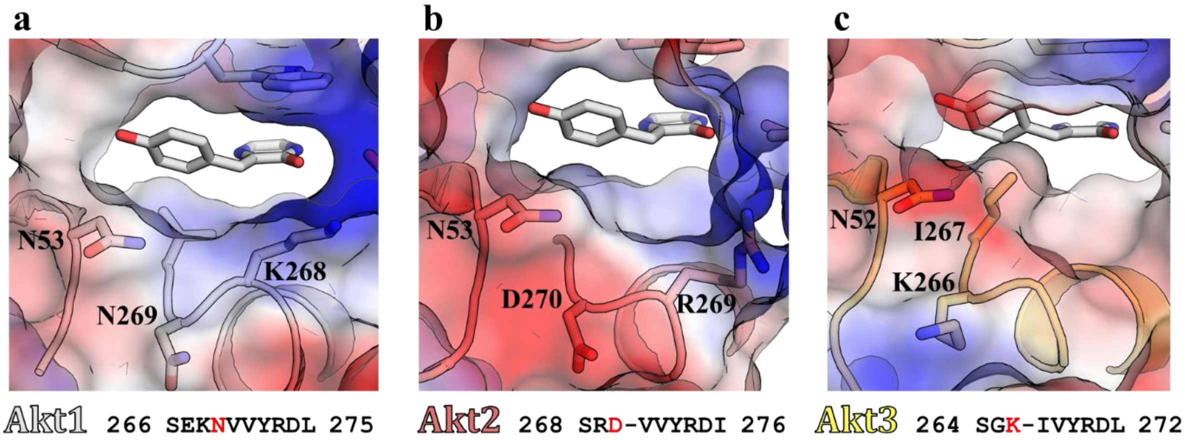

Supplementary Figure 5. Polar surface representation of the Akt isoform homology models and the corresponding sequences. After the c-terminal end of the αE-helix at position 269, each isoform has a different amino acid with distinct chemical properties: an asparagine in Akt1 (A), an aspartate in Akt2 (B), and a lysine in Akt3 (C). The visualization of the polar surfaces indicates the corresponding changes in the electrostatic potential within the different isoform binding pockets. The electrostatic surface was generated with APBS plugin for PyMOL and is defined according to a negative (red), neutral (white), or positive (blue) polarization.[3]

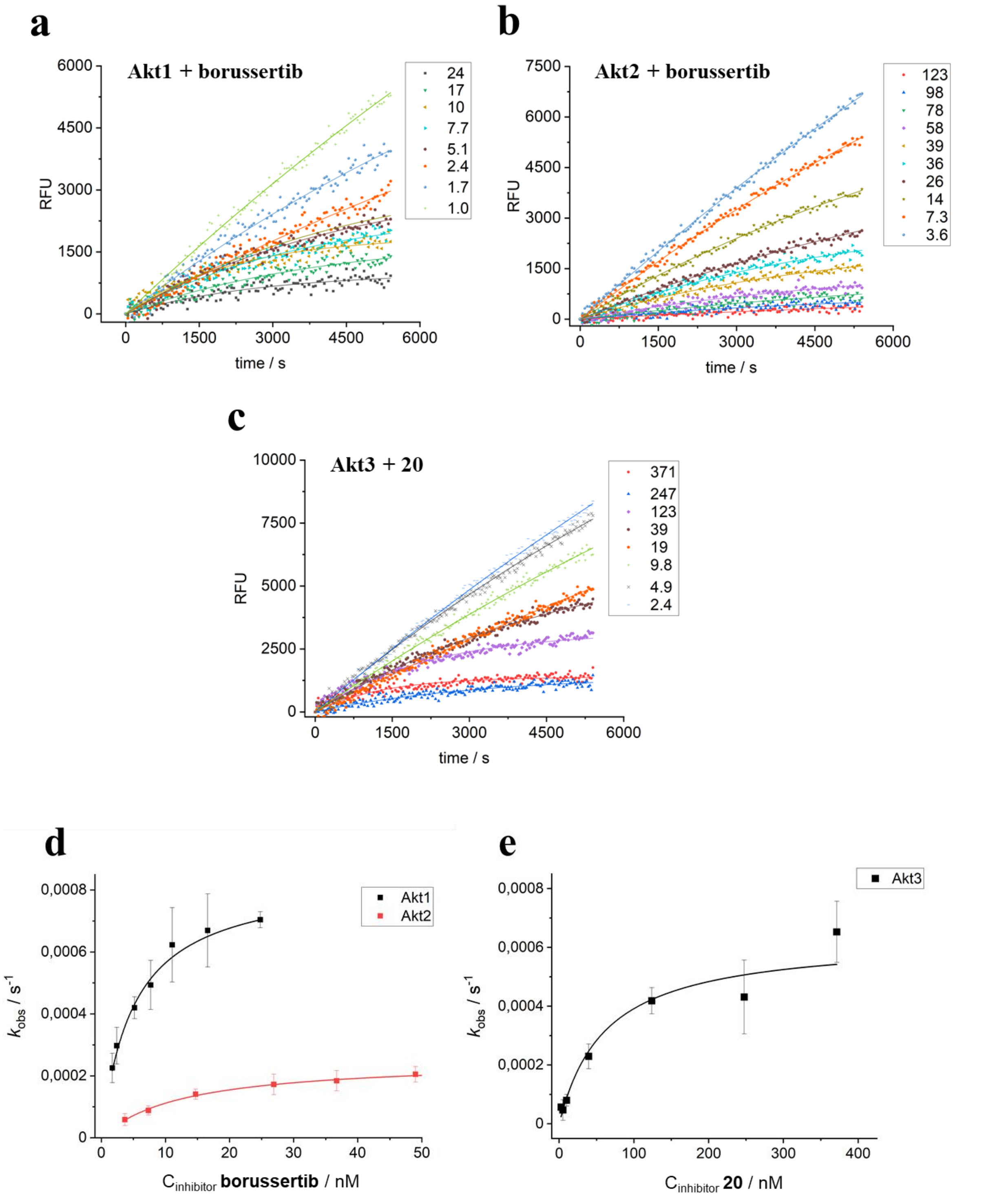

Supplementary Figure 6. Kinetic evaluation of the Akt Isoforms with selected covalent-allosteric inhibitors. (a-c) Exemplifying progression curves of fluorescent product formation over the time in presence of a covalent modifier (RFU: relative fluorescent unit). Akt1 (a) and Akt2 (b) were incubated with different inhibitor concentrations of borussertib, and Akt3 (c) was treated with aminopyridine **20**. The resulting curves of the PhosphoSens assay were fitted an exponential equation according to literature procedure, and gave the observed reactions rate k_{obs} for each inhibitor concentration.[4] The depicted curves represent $n = 1$ independent experiments. (d) Plot of obtained k_{obs}-values against inhibitor concentrations of borussertib for Akt1 (black), and Akt2 (red). (e) Plot of obtained reactions rates against inhibitor concentrations of CAAI **20**. A hyperbolic fit gives the desired kinetic parameters k_{inact} and K_i (see table 2). The graphs in d and e are depicted as mean values ± SD with n = 3 independent experiments. Source Data are provided with this paper.

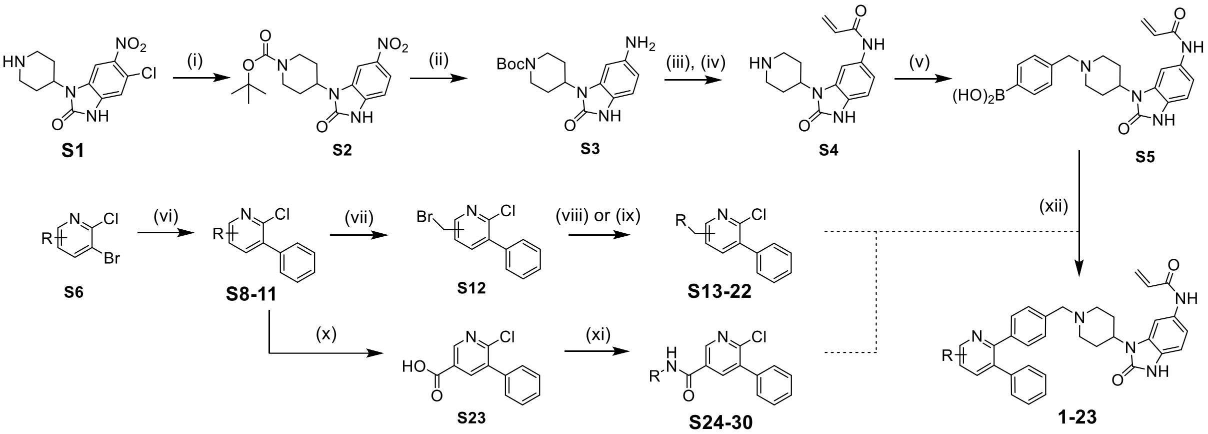

Supplementary Figure 7. Synthesis scheme of pyridine-based covalent-allosteric inhibitors (1-23).[a]

[a]Reagents and conditions: (*i*) $BocO_2$, Et_3N, DCM, rt, 12 h (85 %); (*ii*) Pd/C, NH_4HCOO, MeOH, 80 °C, 16 h (58 %); (*iii*) Acryloylchloride, DIPEA, THF, 0 °C to rt, 12 h (83 %); (*iv*) DCM/TFA (3:1), rt, 12 h (81 %); (*v*) 4-Formyl phenylboronic acid, $NaCNBH_3$, Et_3N/AcOH, MeOH, 75 °C, 12 h (66 %); (*vi*) Phenylboronic acid, $Pd(dppf)_2Cl_2$*DCM, K_3PO_4, 1,4-dioxane/H_2O (5:1), 130 °C, µw, 1 h (44–94 %); (*vii*) *N*-Bromosuccinimide, AIBN, TCM, 90 °C, 1 h (44 %); (*viii*) R-boronic acid/ester, $Pd(dppf)_2Cl_2$*DCM, K_3PO_4, 1,4-dioxane/H_2O (5:1), 75 °C, 12 h (52–85 %); (*ix*) R-NH_2, DMF, K_2CO_3, rt, 2–12 h, (64–69 %); (*x*) THF/MeOH, NaOH, rt, 6 h (90 %); (*xi*) R-NH_2, HATU, DIPEA, MeCN, rt, 16 h, (19–80 %); (*xii*) Pyridine-derivatives, $Pd(PPh_3)_2Cl_2$, K_2CO_3, 1,4-dioxane/H_2O (5:1), 130 °C, µw, 2 h (18–91 %).

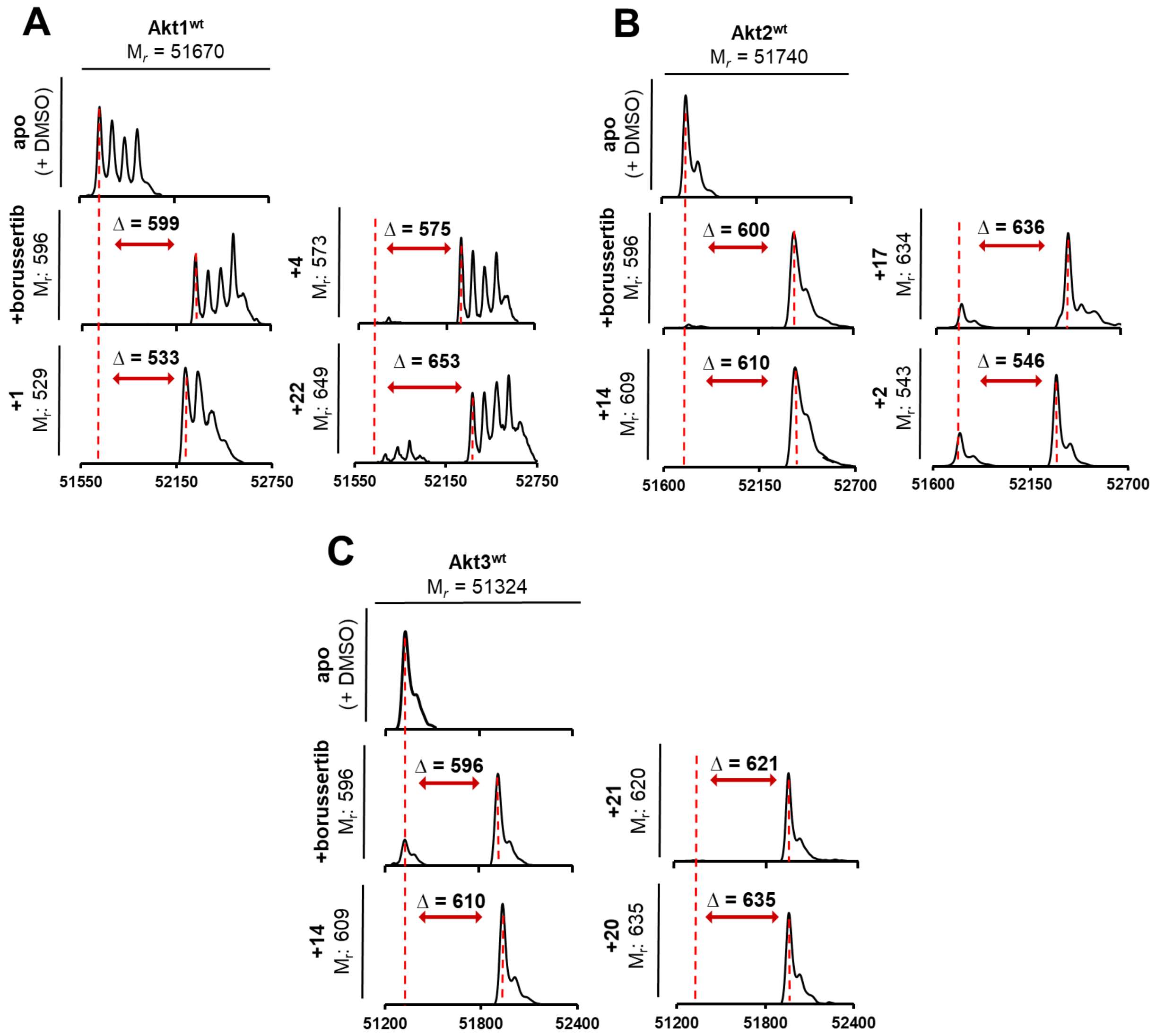

Supplementary Figure 8. Protein mass spectrometry analysis of selected CAAIs with the Akt Isoforms. Deconvoluted mass spectra of Akt1wt (A), Akt2wt (B), and Akt3wt (C) after incubation with DMSO (apo) and selected covalent-allosteric Akt ligands. All tested molecules show mass differences according to a mono-labeling of the protein and the completeness complies with the ligands potency for the specific isoform. Mass spectra were recorded using denaturing conditions. Source Data are provided with this paper.

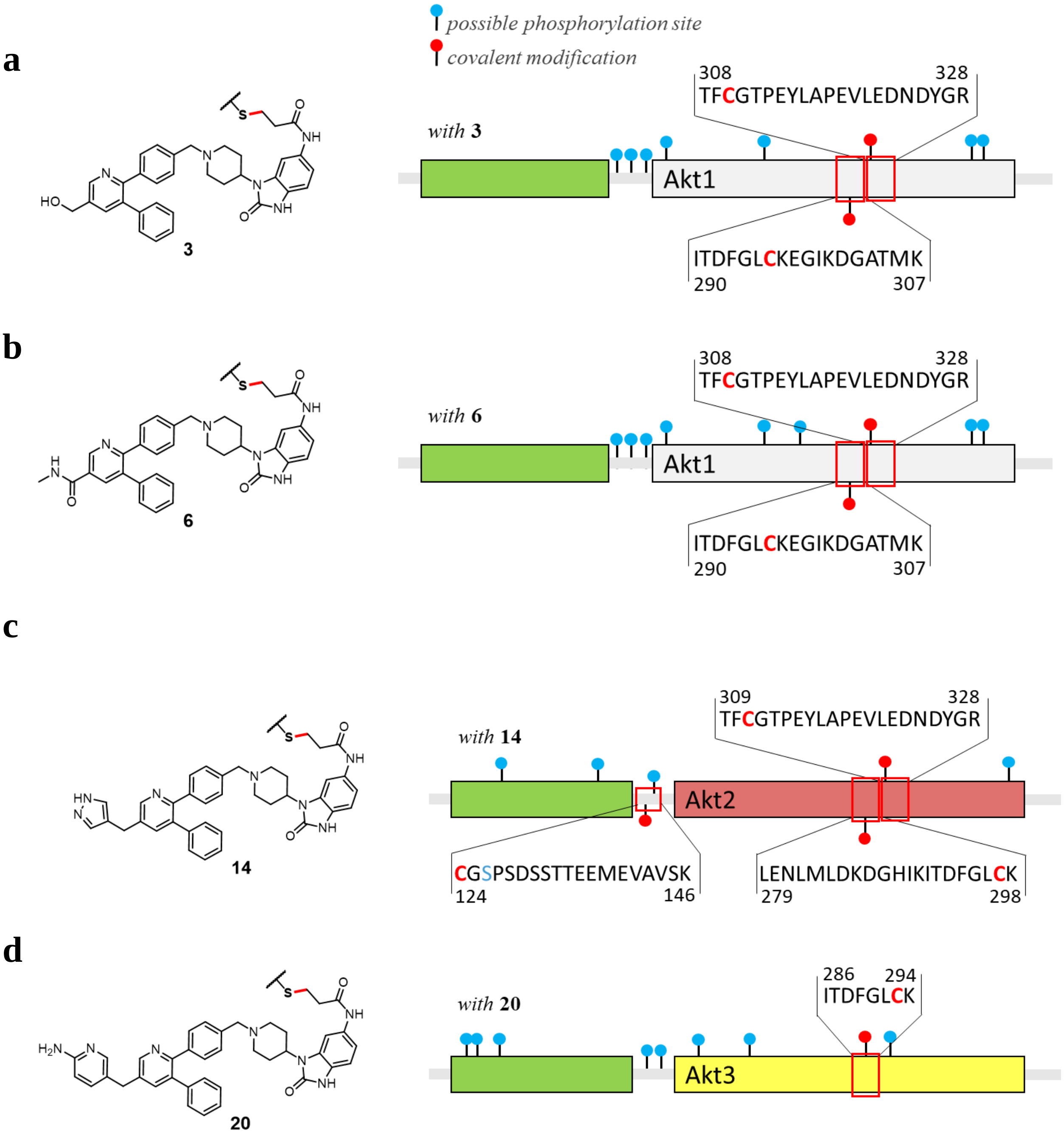

Supplementary Figure 9. CAAI's covalently bind to specific Akt Isoforms at targeted cysteine residues. Full-length Akt Isoforms were incubated with a 5-fold molar excess of inhibitor **3** (**a**) and **6** (**b**) with Akt, molecule **14** (**c**) with Akt2 and molecule **20** (**d**) with Akt3. The modified Proteins were digested with trypsin following standard protocol. Peptide fragments of Akt1 containing Cys296 and Cys310 modification with **3** and **6** were identified, whereas for **20** only a selective labelling of Cys293 in Akt3 could be observed. For Akt2 peptide fragments with modifications at Cys297, Cys311, and Cys124 were found (sequence coverage >90 %).

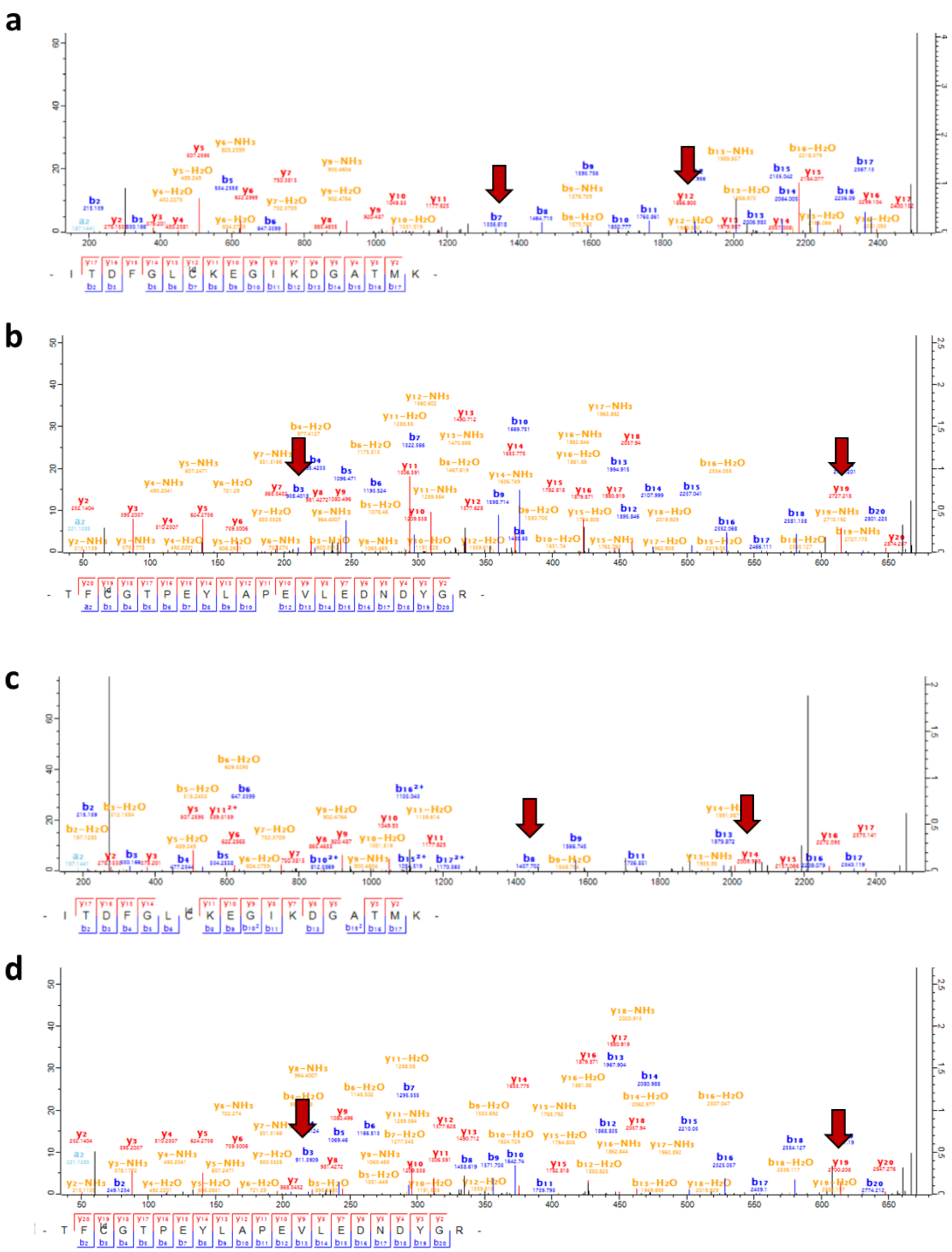

Supplementary Figure 10. Annotated MS/MS-spectra of Akt1^{WT}modified with CAAIs 3 and 6. Full-length Akt Isoforms were incubated with a 5-fold molar excess of inhibitor **3** and **6** with Akt1^{WT}. Annotated Tandem-MS/MS spectra show segmental labelling of Cys296 (a) and Cys310 (b) with CAAI **3**. As well as Cys296 (c) and Cys30(d) with CAAI **6**. The exact Peptide sequence is depicted under the spectra and red arrows mark the identified fragment containing the alkylated residues.

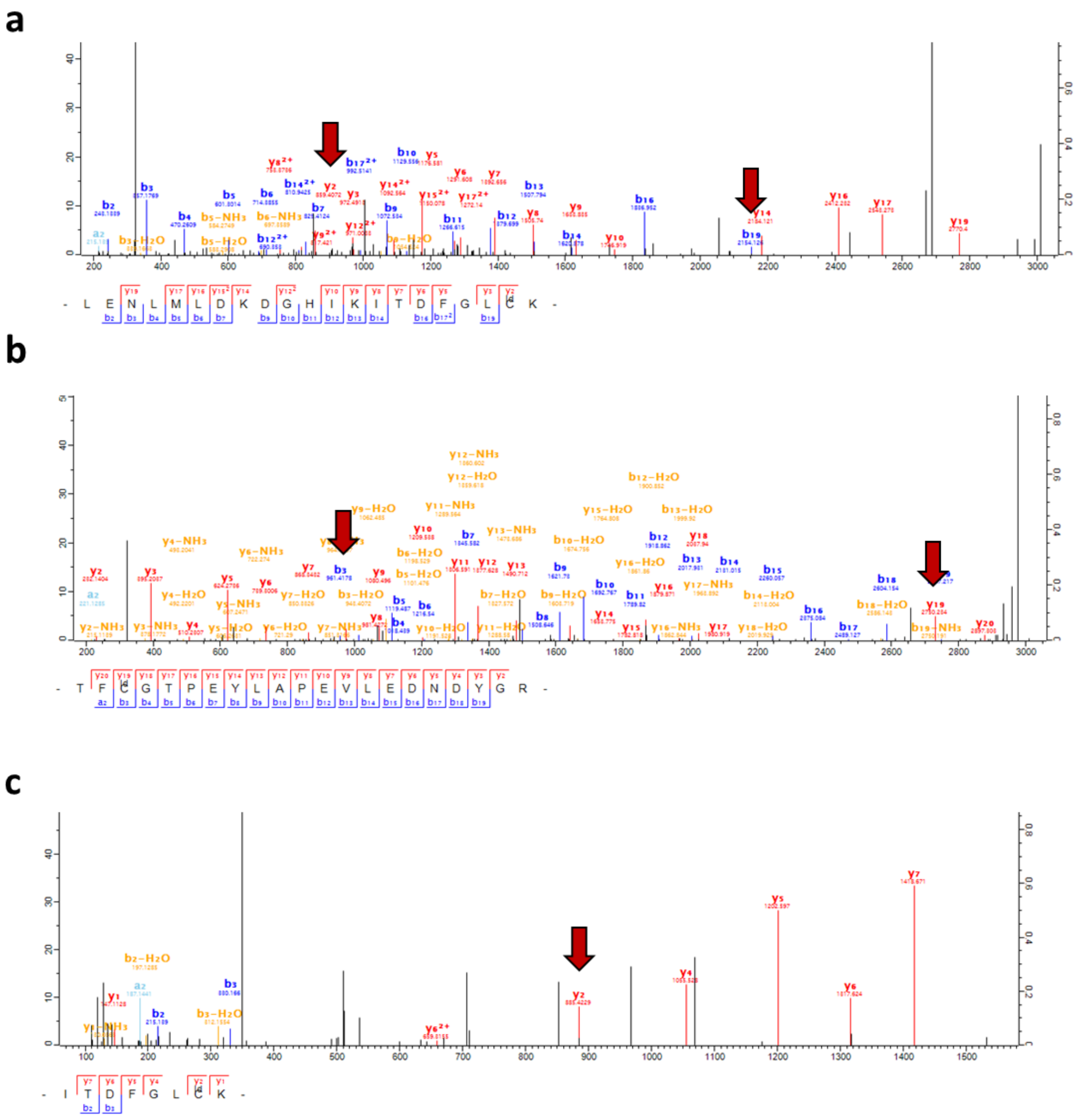

Supplementary Figure 11. Annotated MS/MS-spectra of Akt2^{WT}and Akt3^{WT}modified with CAAIs 14 and 20. Full-length Akt Isoforms were incubated with a 5-fold molar excess of inhibitor **14** with Akt2^{WT} and **20** with Akt3^{WT}. Annotated Tandem-MS/MS spectra show segmental labelling of Cys297 (a) and Cys311 (b) with CAAI **14**. As well as Cys293 (c) with CAAI **20**. The exact Peptide sequence is depicted under the spectra and red arrows mark the identified fragment containing the alkylated residues.

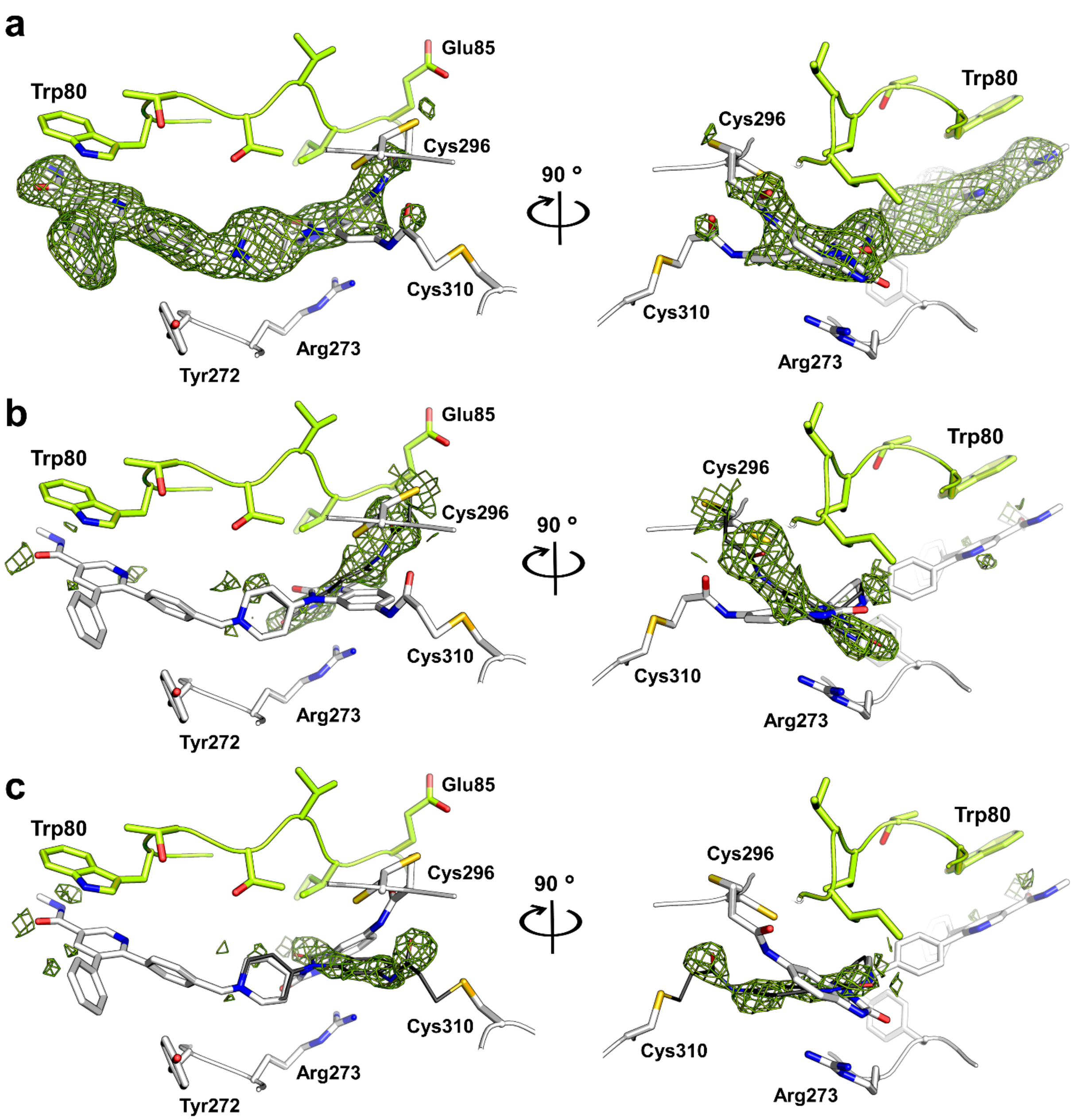

Supplementary Figure 12. Co-crystal structure of Akt1 (2-446) in complex with covalent-allosteric inhibitor 6 (PDB ID 7NH5). (a) Modelling of two ligand conformations targeting Cys296 and Cys310, indicated by the F_O-F_C simulated annealing omit map (σ = 2). The covalent bond formation of both cysteine residues was confirmed by tandem mass spectrometry (Fig. S4). (b) Modelling of **6** bound to Cys310. The F_O-F_C simulated annealing omit map (σ = 2) of the alternative ligand conformation (depicted as black lines) is shown. (c) Modelling of **6** bound to Cys296. The F_O-F_C simulated annealing omit map (σ = 2) of the alternative ligand conformation (depicted as black lines) is shown.

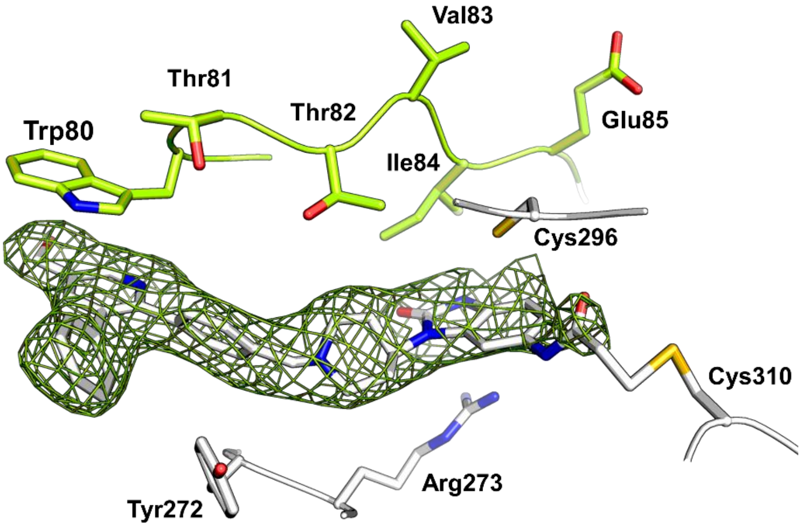

Supplementary Figure 13. Co-crystal structure of Akt1 (2-446) in complex with covalent-allosteric inhibitor 3 (PDB ID 7NH4). The F_O-F_C simulated annealing omit map (σ = 2) is shown.

Supplementary Table 1. Crystallographic statistics of Akt1 (2-446) in complex with 3 (PDB ID 7NH4) and 6 (PDB ID 7NH5). Values in parenthesis refer to the highest resolution shell**.**

**Data collection**		
	**3**<br>PDB: 7NH4	**6**<br>PDB: 7NH5
Space group	$P2_12_12_1$	$P2_12_12_1$
Cell dimensions		
a, b, c (Å)	70.76, 70.95, 91.13	70.65, 71.41, 91.02
α, β, γ (°)	90.00, 90.00, 90.00	90.00, 90.00, 90.00
Resolution (Å)	45.56 – 2.30 (2.40 – 2.30)	43.97 – 1.90 (1.90 – 2.00)
R_{meas} (%)	6.40 (161.40)	5.00 (188.50)
R_{merge} (%)	6.00 (150.20)	4.80 (181.70)
I/σI	15.03 (1.05)	25.85 (1.47)
$CC_{1/2}$	99.90 (60.80)	100.00 (67.90)
Completeness (%)	99.90 (100.00)	100.00 (100.00)
Redundancy	7.42 (7.50)	13.53 (14.14)
**Refinement**		
Resolution (Å)	45.56 – 2.30	43.97 – 1.90
No. reflections	20,958	36,964
R_{work}/R_{free}	22.28/24.57 (33.86/36.77)	19.98/22.84 (34.80/37.23)
No. atoms		
Protein	3,045	3,149
Ligand	42	65
Acetate	8	4
Water	15	83
B-factors		
Protein	82.18	58.00
Ligand	73.12	42.04
Acetate	102.70	76.33
Water	68.24	56.10
rms deviations		
Bond lengths (Å)	0.002	0.010
Bond angles (°)	0.494	1.084
Wavelength (Å)	0.99999	0.99998
Temperature (K)	100	100
X-ray source	PX II at SLS, Villigen, CH	PX II at SLS, Villigen, CH
Detector	Eiger2 X 16M	Eiger2 X 16M
**Ramachandran Plot**		
Residues in		
favored regions	97.32 %	97.32 %
allowed regions	2.68 %	2.68 %
outlier regions	0.00 %	0.00 %

Supplementary Table 2. Cellular evaluation of synthesized covalent-allosteric Akt inhibitors in myr-Akt isoform dependent BaF3 cell lines and the parental BaF3 cell line. The mean EC_{50} values ± s.d. were calculated from experimental points measured in duplicates for each plate and were replicated in n = 3 biologically independent experiments. Source Data are provided with this paper.

	**EC_{50} /nM**											
**#**	**$BaF3^{myr\text{-}Akt1}$**			**$BaF3^{myr\text{-}Akt2}$**			**$BaF3^{myr\text{-}Akt3}$**			**BaF3**		
**1**	1787	±	637	4473	±	2127	> 30000			6894	±	692
**2**	3095	±	307	2785	±	1205	> 30000			11455	±	1236
**3**	28324	±	1676	21194	±	8807	> 30000			> 30000		
**4**	> 30000			> 30000			> 30000			> 30000		
**5**	4919	±	689	2714	±	1039	7650	±	202	16331	±	2626
**6**	9614	±	1622	10803	±	2563	25686	±	3148	> 30000		
**7**	4711	±	863	1781	±	719	2509	±	522	8462	±	1441
**8**	> 30000			12785	±	948	26478	±	1737	25613	±	5480
**9**	> 30000			13602	±	2591	14110	±	3455	29492	±	718
**10**	26661	±	3929	7987	±	1821	24273	±	1724	24525	±	6003
**11**	28817	±	1242	2690	±	355	18561	±	3885	> 30000		
**12**	8290	±	1252	3046	±	672	5288	±	2956	24711	±	5297
**13**	19283	±	1743	7179	±	679	22620	±	4994	25580	±	8253
**14**	7557	±	482	529	±	51	4468	±	148	8643	±	1221
**15**	26685	±	2087	14308	±	5914	17160	±	1422	28578	±	2011
**16**	> 30000			25496	±	6370	> 30000			> 30000		
**17**	> 30000			3750	±	426	22036	±	2694	> 30000		
**18**	17806	±	499	1479	±	710	18153	±	7820	> 30000		
**19**	12736	±	4925	2893	±	688	7442	±	642	12490	±	5265
**20**	9483	±	366	4093	±	806	5054	±	595	12145	±	3732
**21**	19686	±	4737	2234	±	194	6515	±	654	14739	±	2368
**22**	22535	±	6092	3683	±	1254	18840	±	1056	> 30000		
**23**	14641	±	1219	2760	±	504	17064	±	7359	> 30000		
**15a**	7211	±	600	14866	±	4051	26950	±	5283	> 30000		
**16b**	29835	±	130	6343	±	597	21959	±	7802	20125	±	3002
**borussertib**	440	±	99	599	±	214	6837	±	670	20068	±	3660
**MK-2206**	1124	±	100	1339	±	449	5734	±	169	7386	±	1553
**capivasertib**	1074	±	223	938	±	240	848	±	103	15997	±	5100

Supplementary Table 3. Selectivity ratios of small molecule library based on in vitro results. Ratios of selectivity in log units obtained from a biochemical activity based assay in comparison to the ratios obtained from cell viability assay with Ba/F3 myr-Akt isoform dependent cells.

	Log ratio IC50 [Akt] / IC50 [Akt]		Log ratio EC50 [Akt] / EC50 [Akt]	
**#**	**Akt1 / Akt2**	**Akt3 / Akt2**	**Akt1 / Akt2**	**Akt3 / Akt2**
**1**	-1.05	-1.04	-0.40	-0.83
**2**	-0.83	-1.70	0.05	-0.46
**3**	-0.71	-1.32	0.13	-0.15
**4**	-0.75	-1.41	0	0
**5**	-0.52	-1.47	0.26	-0.45
**6**	0.02	-1.73	-0.05	-0.38
**7**	1.17	-1.62	0.42	-0.15
**8**	0.34	-0.99	0.37	-0.32
**9**	0.15	-0.30	0.34	-0.02
**10**	1.26	-1.22	0.52	-0.48
**11**	0.93	-1.40	1.03	-0.84
**12**	0.71	-0.38	0.43	-0.24
**13**	0.27	-1.83	0.43	-0.50
**14**	1.35	-1.19	1.15	-0.93
**15**	-0.37	-0.49	0.27	-0.08
**16**	1.14	-0.89	0.07	-0.07
**17**	1.33	-1.20	0.90	-0.77
**18**	0.37	-2.13	1.08	-1.09
**19**	0.55	-0.47	0.64	-0.41
**20**	0.97	-0.03	0.27	-0.09
**21**	1.30	-0.43	0.95	-0.46
**22**	0.58	-1.74	0.79	-0.71
**23**	-0.07	-1.62	0.72	-0.79
**15a**	-2.52	-0.19	-0.31	-0.26
**16b**	0.98	-2.14	0.67	-0.54
**borussertib**	-1.08	-1.71	-0.13	-1.06
**MK-2206**	-0.99	-0.78	-0.08	-0.63
**capivasertib**	-0.40	-0.20	0.06	0.04

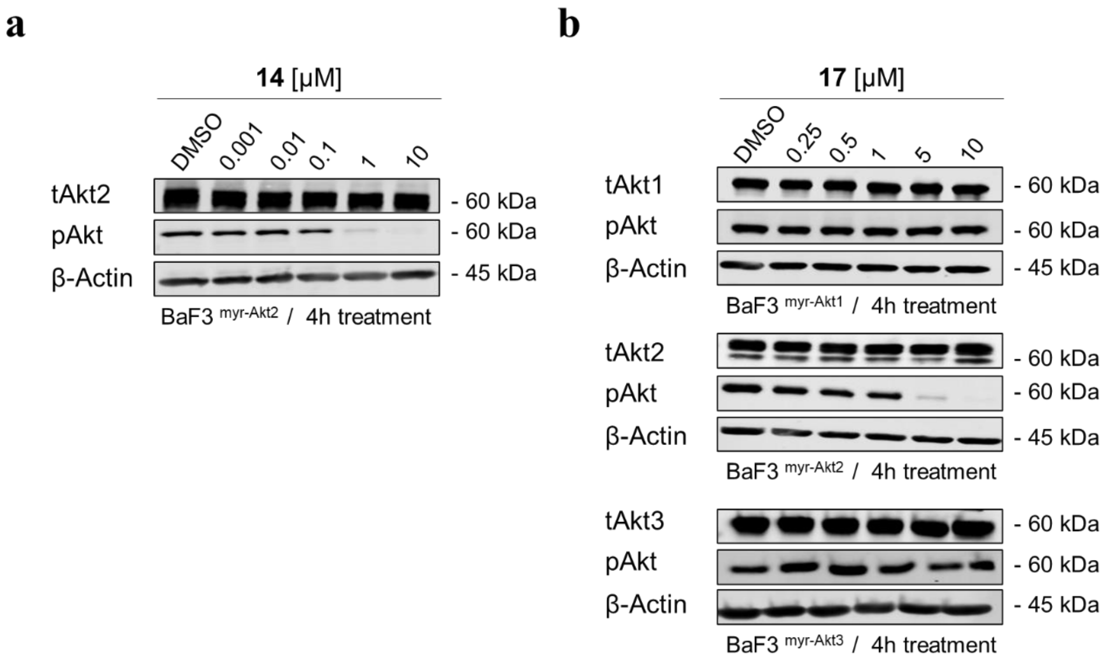

Supplementary Figure 14. BaF3 myr-Akt isoform dependent cell lines treated with Akt inhibitors 14 and 17. (a) Immunoblot of BaF3 myr-Akt2 treated with lower concentrations of **14** for the visualization of a dose-dependent downregulation of Akt2 (n = 1 biologically independent experiment). **(b)** Immunoblot of BaF3 myr-Akt isoform dependent cell lines treated with **17** (n = 1 biologically independent experiment). Source Data are provided with this paper.

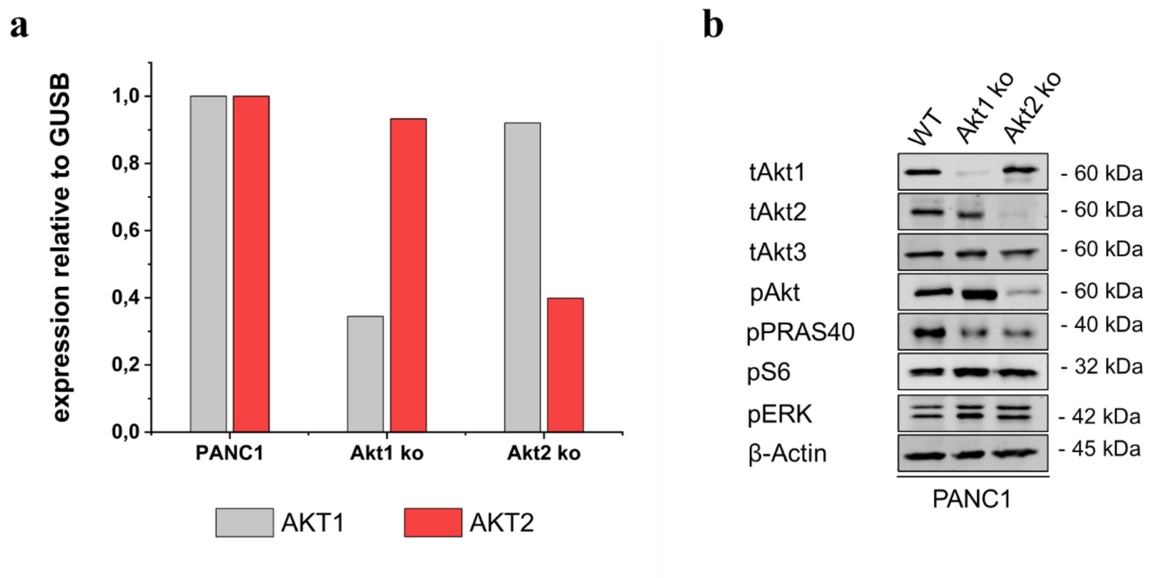

Supplementary Figure 15. PANC1 Akt1 knockout and PANC1 Akt2 knockout models. (a) Quantitative PCR (qPCR) analysis, depicting relative expression levels of Akt1/Akt2 normalized to GUSB (n = 1 biologically independent experiment). **(b)** Immunoblot of PANC1 Akt1/Akt2 ko cell lines in comparison to the parental PANC1 cell line highlighting the molecular expression levels, activities and downstream targets of Akt (n = 1 biologically independent experiment). Source Data are provided with this paper.

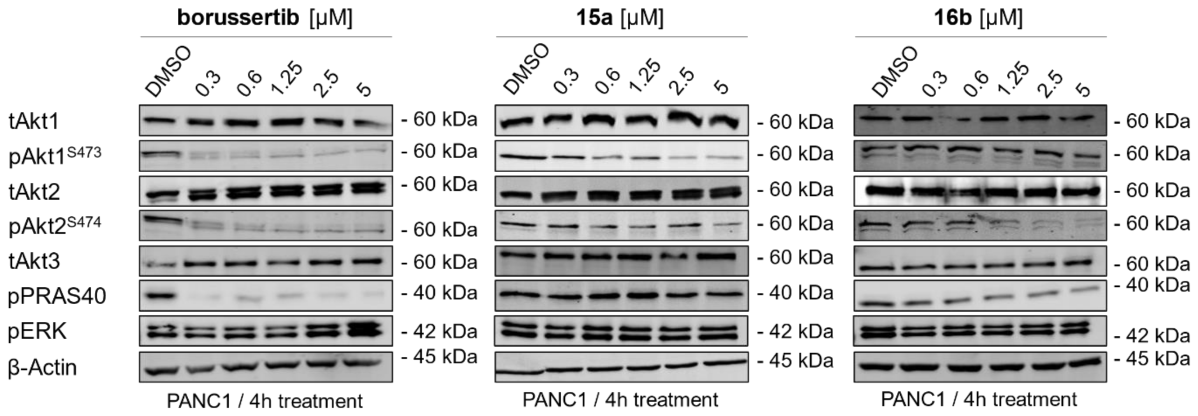

Supplementary Figure 16. Immunoblots of PANC1 treated with borussertib, 15a and 16b. Immunoblots to elucidate dose-dependent downregulation of individual Akt isoform activity after 4-hour treatment, with pAkt1 and pAkt2 specific antibodies (n = 1 biologically independent experiment). Source Data are provided with this paper.

a

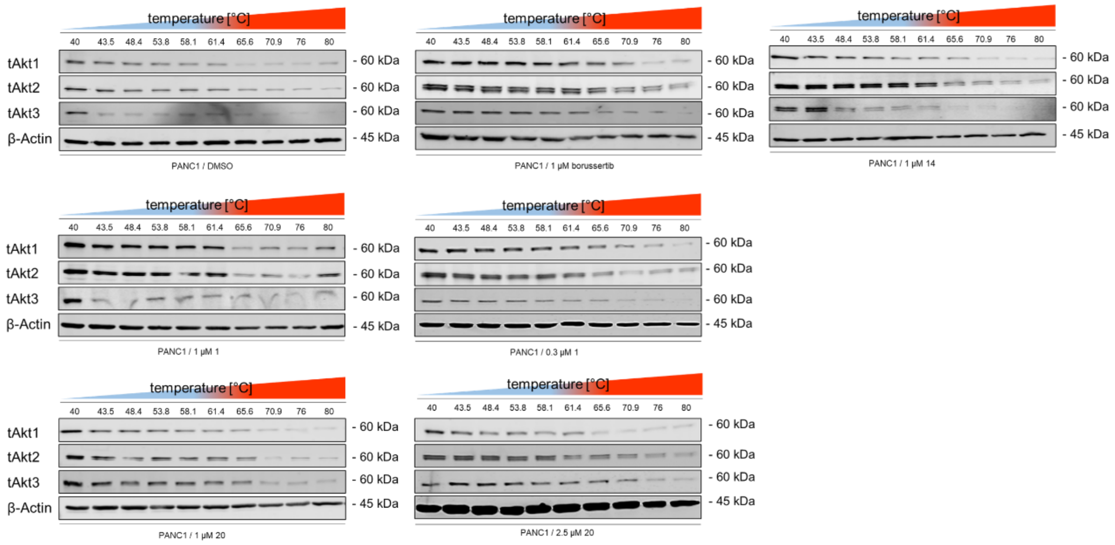

b

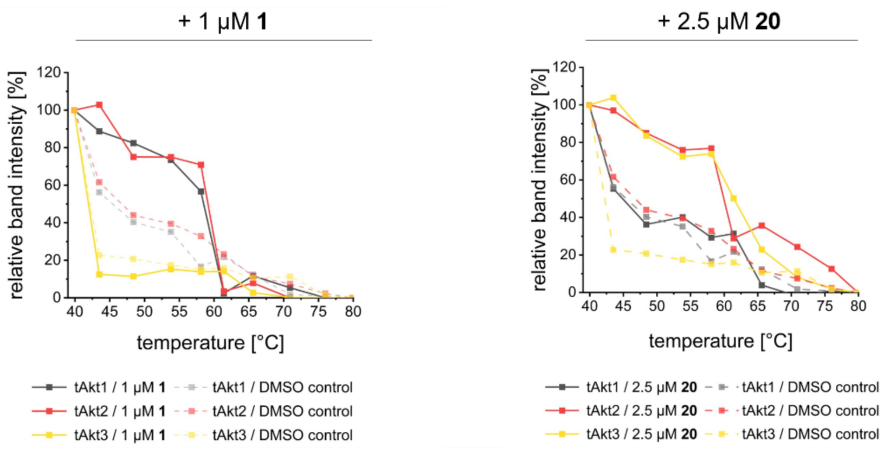

Supplementary Figure 17. Overview of CETSA experiments in PANC1 cells with promising CAAIs. (a) Immunoblots of CETSA experiments with cell line PANC1 treated with molecules borussertib, **1**, **14** and **20** and DMSO as a control. (b) Quantitative analysis of PANC1 CETSA experiments. Treatment with molecules **1** and **20** in comparison with DMSO. The experiment was performed once with n = 1 biologically independent experiment. Source Data are provided with this paper.

Supplementary Table 4. List of primers.

pBABE Seq fw	CTTTATCCAGCCCTCAC
pBABE Seq rv	ACCCTAACTGACACACATTCC
Myr-Tag Seq fw	ATGGGGAGCAGCAAGAGCAAG
HA-Tag Seq fw	TACGCCTACGACGTGCCCGAC

Supplementary Methods

General Chemistry. All reagents and solvents were purchased from Acros, Activate Scientific, Alfa Aesar, Apollo Scientific, Merck, Sigma-Aldrich, TCI Chemicals or VWR and used without further purification. Dry solvents were purchased as anhydrous reagents from commercial suppliers. ^{1}H and ^{13}C NMR spectra were recorded on a Bruker Avance AV500 (500 MHz and 125 MHz), AV600 (600 MHz and 151 MHz) and AV700 (700 MHz and 176 MHz). ^{1}H chemical shifts are reported in λ (ppm) as s (singlet), d (doublet), dd (doublet of doublet), t (triplet), q (quartet), m (multiplet) and are referenced to the residual solvent signal: $CDCl_3$-*d* (7.26), DMSO-d_6 (2.50) or MeOD-d_4 (3.34). ^{13}C spectra are referenced to residual solvent signal: $CDCl_3$-*d* (77.1), DMSO-d_6 (39.52) or MeOD-d_4 (49.86). High-resolution electrospray ionization mass spectra (ESI-FTMS) were recorded on a Thermo LTQ Orbitrap (high-resolution mass spectrometer from Thermo Electron) coupled to an Accela HPLC system supplied with a Hypersil GOLD column (Thermo Electron). LC-MS (ESI-MS) analysis was performed using Agilent HPLC system (1100 series) with CC 125/4 Nucleodur C18 gravity column (3 µm) from Macherey Nagel coupled to a Thermo Scientific Finnigan LCQ Advantage Max Ion Trap and ESA Corona detector. Analytical TLC was carried out on Merck 60 F254 aluminium-backed silica gel plates. Compounds were purified by column chromatography using VWR silica gel (40 - 63 µm particle size) or flash chromatography on a Biotage Isolera One and Büchi Reveleris system using Büchi Reveleris Silica Cartridges (4 - 120 g) monitored by UV at λ = 210 nm and 280 nm. Preparative HPLC was conducted on an Agilent HPLC system (1200 series) with a VP 125/21 Nucleodur C18 column from Macherey-Nagel and monitored by UV at λ = 210 nm and 254 nm. All final compounds were purified to > 95 % purity as determined by high-performance liquid chromatography (HPLC). Purity was measured using Büchi Reveleris Prep system with UV detection at λ = 210 nm (system: Nucleodur C18 gravity column from Macherey Nagel 4 mm x 125 mm, 3 µM, 10 – 100 % MeCN in H_2O, with 0.2 % TFA, for 15 min at 1.0 mL/min).

Synthetic Procedures.

Common Procedure A: Suzuki cross coupling with Phenylboronic acid

In a microwave vessel under argon atmosphere 1.0 eq. of the bromo-pyridine and 1.1 eq of phenylboronic acid were dissolved in 1,4-dioxane and water (5:1, 2 mL/mmol). The mixture was degassed for 10 min and then 2.0 eq. of the base K_3PO_4 as well as 0.1 eq. of [1,1'-Bis(diphenylphosphino)ferrocene]palladium(II)dichloride were added subsequently. Afterwards the suspension was stirred under microwave irridation for 1 h at 130 °C. The resulting suspension was filtered over Celite and extracted with DCM, the combined organic fractions were washed with saturated NaCl solution, dried over Na_2SO_4 and evaporated *in vacuo*. Silica gel column chromatography (1 – 30 % EtOAc/PE) yielded the desired product.

Common Procedure B: Suzuki cross coupling at benzylic position

Under argon atmosphere 1.0 eq. of the pyridine derivative and 1.2 eq. of boronic acid/ester were dissolved in 1,4-dioxane and water (5:1, 2 mL/mmol). The mixture was degassed for 10 min and then 2.0 eq. of the base K_3PO_4 as well as 0.1 eq. of [1,1'-Bis(diphenylphosphino)ferrocene]palladium(II)dichloride were added subsequently. Afterwards the suspension was stirred for 12 h at 75 °C. The resulting suspension was filtered over Celite and extracted with DCM, the combined organic fractions were washed with saturated NaCl solution, dried over Na_2SO_4 and evaporated *in vacuo*. Silica gel column chromatography (1 – 4 % MeOH/DCM + 1 % NH_3) yielded the desired product.

Common Procedure C: Nucleophilic substitution at benzylic position

First, 1.0 eq. of the pyridine and 3.0 eq. K_2CO_3 were suspended in DMF or THF (3 mL/mmol) and then 2.0 eq. of the nucleophile was added. The suspension was stirred and heated for 12 h at rt - 90 °C. The solution was extracted with dichloromethane, the combined organic fractions washed with NaCl solution and dried over Na_2SO_4 before the solvent was evaporated *in vacuo*. The desired product was obtained after silica gel column chromatography (4 - 8 % MeOH/DCM + 1 % NH_3).

Common Procedure D: Amide Coupling

In a round bottom flask 1.0 eq. of the nicotinic acid was suspended in Acetonitrile (5 mL/mmol), then 3.0 eq. of DIPEA and 1.5 eq. of HATU were added and stirred at room temperature for at least 30 min. Afterwards, 1.2 eq. of the amine were added slowly, and the suspension was allowed to be stirred at room temperature until completion. The solution was extracted with dichloromethane, the combined organic fractions washed with NaCl solution, dried over Na_2SO_4 and the solvent was evaporated *in vacuo*. The desired product was obtained after silica gel column chromatography (2 – 5 % MeOH/DCM + 1 % NH_3).

Common Procedure E: Suzuki cross coupling as final step

In a microwave vessel under argon atmosphere 1.0 eq. of the pyridine-derivative and 1.1 eq. of (4-((4-(6-acrylamido-2-oxo-2,3-dihydro-1H-benzo[d]imidazol-1-yl)piperidin-1-yl)methyl)phenyl)boronic acid (were dissolved in 1,4-dioxane and water (5:1, 2 mL/mmol). The mixture was degassed for 10 min and then 2.0 eq. of the base K_2CO_3 as well as 0.1 eq. of tetrakis(triphenylphosphine)palladium(0) were added subsequently. Afterwards the suspension was stirred under microwave irridation for 2 h at 130 °C. The resulting suspension was filtered over Celite and extracted with DCM, the combined organic fractions were washed with saturated NaCl solution, dried over Na_2SO_4 and evaporated *in vacuo*. Silica gel column chromatography (6 – 10 % MeOH/DCM + 1 % NH_3) yielded the desired product.

Synthesis of 5-chloro-6-nitro-1-(piperidin-4-yl)-1,3-dihydro-2H-benzo[d]imidazol-2-one (**S1**) has been reported.[2]

Synthesis of tert-butyl 4-(5-chloro-6-nitro-2-oxo-2,3-dihydro-1H-benzo[d]imidazol-1-yl)piperidin-1-carboxylate (**S2**).
S1 (5.4 g, 18.2 mmol) was stirred in 50 mL DCM and 2.4 mL of triethylamine (20.2 mmol, 1.1 eq.) were added. Then, 1.2 eq. Boc_2O (4.4 mL, 21.8 mmol) was dissolved in 10 mL DCM and then added dropwise to the reaction mixture, which was allowed to stir overnight at room temperature. Afterwards, water (30 mL) was added to the suspension and the aqueous layer extracted with DCM. The combined organic fractions were washed with $NaHCO_3$ solution. After drying with Na_2SO_4, filtration and evaporation of the solvents, a silica gel column chromatography was performed (1-2 % MeOH/DCM + 1 % NH_3) which yielded the titled compound as a yellow solid **S2** (6.3 g, 15.9 mmol, 85 %). **^{1}H-NMR** (600 MHz, $CDCl_3$-*d*) δ ppm 1.66 - 1.70 (m, 9H) 1.80 (dd, *J* = 12.15, 1.74 Hz, 4H) 2.32 (m, 4H) 4.38 (m, 1H) 7.66 (s, 1H) 8.14 (s, 1H); **^{13}C-NMR** (151 MHz, $CDCl_3$-*d*) δ ppm 27.98 (s, 2C) 52.39 (s, 3C) 80.28 (s, 2C) 86.60 (s, 1C) 105.80 (s, 1C) 117.23 (s, 1C) 121.89 (s, 1C) 127.91 (s, 1C) 129.91 (s, 1C) 143.35 (s, 1C) 147.96 (s, 1C) 149.80 (s, 1C) 154.52 (s, 1C); **HPLC-MS (ESI):** *m/z* for $C_{17}H_{22}N_4O_5Cl$ (**[M+H$^+$]**), 397.83 calcd., 397.40 found.

Synthesis of tert-butyl 4-(6-amino-2-oxo-2,3-dihydro-1H-benzo[d]imidazol-1-yl)piperidin-1-carboxylate (**S3**).
S2 (5.0 g, 12.0 mmol, 1.0 eq.), 5 % Pd/C moistened with water (0.2 g) and ammonium formate (7.56 g, 120.0 mmol, 10 eq.) were dissolved in methanol (36 mL, 3 mL/mmol) and allowed to stir for 16 h at 80 °C. The reaction mixture was filtered over Celite, evaporated and the crude product was dissolved in DCM and washed with $NaHCO_3$ solution. After drying with Na_2SO_4, filtration and evaporation of the solvents, a silica gel column chromatography was performed (3-4 % MeOH/DCM + 1 % NH_3) which yielded the titled compound as a pale rose solid **S2** (2.32 g, 7.0 mmol, 58 %). **^{1}H-NMR** (500 MHz, DMSO-*d_6*) δ ppm 1.55 (s, 9H) 1.69 (d, *J* = 10.99 Hz, 4H) 2.15 (m, 4H) 4.23 (m, 1H) 6.30 (dd, *J* = 8.62, 2.06 Hz, 1H) 6.53 (s, 1H) 7.38 (s, 1H); **^{13}C-NMR** (126 MHz, DMSO-*d_6*) δ ppm 28.57 (s, 2C) 50.95 (s, 3C) 79.35 (s, 2C) 83.74 (s, 1C) 95.49 (s, 1C) 107.62 (s, 1C) 114.99 (s, 1C) 116.22 (s, 1C) 129.65 (s, 1C) 146.28 (s, 1C) 148.80 (s, 1C) 150.13 (s, 1C) 154.26 (s, 1C); **HPLC-MS (ESI):** [R_t]: 5.03 min, *m/z* for $C_{17}H_{25}N_4O_3$ ([M+H$^+$]), 333.40 cacld., 333.84 found.

Synthesis of tert-butyl 4-(6-acrylamido-2-oxo-2,3-dihydro-1H-benzo[d]imidazol-1-yl)piperidin-1-carboxylate (**S4a**).
S3 (2.32 g, 7.0 mmol 1.0 eq.), was dissolved in dry THF (10 mL/mmol), 3 eq. DIPEA (3.4 mL, 21.0 mmol) were added and the solution was stirred for 15 min at 0 °C under argon atmosphere. Subsequently, a solution of 1.0 eq. acryloyl chloride (0.5 mL, 7.0 mmol) in 5 mL THF was added dropwise. The reaction mixture was stirred at room temperature overnight. Afterwards the reaction was quenched with a saturated solution of $NaHCO_3$. The aqueous layer was extracted with DCM and the combined organic layers were washed with saturated NaCl solution and dried over Na_2SO_4. The solvent was evaporated *in vacuo* and silica gel column chromatography (3 - 4 % MeOH/DCM + 1 % NH_3) yielded the desired product **S4a** (2.2 g, 5.8 mmol, 83 %). **^{1}H-NMR** (500 MHz, DMSO-*d_6*) δ ppm 1.58 (s, 9H) 1.74 (d, *J* = 12.05 Hz, 4H) 2.15 (m, 4H) 4.32 (m, 1H) 5.76 (d, *J* = 7.48 Hz, 2H) 6.25 (dd, *J* = 17.01, 1.91 Hz, 1H) 7.66 (d, *J* = 8.70 Hz, 1H) 7.83 (d, *J* = 1.68 Hz, 1H) 10.24 (s, 1H); **^{13}C-NMR** (126 MHz, DMSO-*d_6*) δ ppm 28.11 (s, 2C) 51.17 (s, 3C) 55.40 (s, 2C) 79.43 (s, 1C) 84.45 (s, 1C) 101.18 (s, 1C) 114.47 (s, 1C) 121.90 (s, 1C) 127.43 (s, 1C) 128.83 (s, 1C) 132.22 (s, 1C) 135.78 (s, 1C) 148.58 (s, 1C) 149.99 (s, 1C) 154.31 (s, 1C) 163.54 (s, 1C); **HPLC-MS (ESI):** [R_t]: 7.34 min, *m/z* for $C_{20}H_{27}N_4O_4$ ([M+H$^+$]), 387.45 cacld., 387.81 found.

Synthesis of N-(2-oxo-3-(piperidin-4-yl)-2,3-dihydro-1H-benzo[d]imidazol-5-yl)acrylamide (**S4**).
S4a (2.2 g, 5.8 mmol, 1.0 eq.) in DCM and TFA (3:1) (5 mL/mmol) was stirred for 12 h at room temperature. Afterwards the reaction mixture was basified with 10 M NaOH solution. The resulting precipitate was separated and extracted with dichloromethane. The combined organic fractions were dried over Na_2SO_4 and the solvent was evaporated *in vacuo*. The pure product **S4** (1.3 g, 4.7 mmol, 81 %) was obtained after silica gel column chromatography (7 – 8 % MeOH/DCM + 1 % NH_3). **^{1}H-NMR** (500 MHz, DMSO-*d_6*) δ ppm 1.88 (d, *J* = 12.51 Hz, 1H) 2.55 (m, 4H) 3.12 (q, *J* = 12.00 Hz, 4H) 4.42 (m, 1H) 5.99 (dd, *J* = 252.23, 17.09 Hz, 1H) 6.45 (dd, *J* = 17.01, 10.15 Hz, 1H) 6.92 - 6.96 (m, 1H) 6.98 (s, 1H) 7.85 (s, 1H); **^{13}C-NMR** (126 MHz, DMSO-*d_4*) δ ppm 26.11 (s, 2C) 43.49 (s, 2C) 47.96 (s, 1C) 102.11 (s, 1C) 109.31 (s, 1C) 113.80 (s, 1C) 125.17 (s, 1C) 126.88 (s, 1C) 129.45 (s, 1C) 132.82 (s, 1C) 154.41 (s, 1C) 163.31 (s, 1C); **HPLC-MS (ESI):** [R_t]: 3.25 min, *m/z* for $C_{15}H_{19}N_4O_2$ ([M+H$^+$]), 287.34 cacld., 287.18 found.

Synthesis of (4-((4-(6-acrylamido-2-oxo-2,3-dihydro-1H-benzo[d]imidazol-1-yl)piperidin-1-yl)methyl)phenyl)boronic acid (**S5).**
S4 (1.0 g, 3.5 mmol, 1.0 eq.) and 4-formylphenylboronic acid (625 mg, 4.2 mmol, 1.2 eq.) were dissolved in dry MeOH (5 mL/mmol), then a few drops of Et_3N and AcOH were added and the solution was stirred for 4 h at 75 °C under argon atmosphere. Afterwards, 4.0 eq. $NaCNBH_3$ (877 mg, 13.9 mmol) was added slowly and the reaction mixture was allowed to stir at 75 °C overnight. The precipitate was filtered and the solvent evaporated. The crude product was dissolved in DCM and washed with saturated $NaHCO_3$ solution. After drying over Na_2SO_4 and evaporation of the solvents *in vacuo,* a reversed phase column chromatography (Water/MeCN + 0.01 % TFA) yielded the desired product **S5** (980 mg, 2.3 mmol, 66 %). **^{1}H-NMR** (700 MHz, DMSO-*d_6*) δ ppm 1.94 (d, *J* = 12.80 Hz, 2H) 2.63 (d, *J* = 12.80 Hz, 2H) 3.22 (d, J = 11.40 Hz, 2H) 3.50 (d, *J* = 11.19 Hz, 2H) 4.34 (d, *J* = 3.98 Hz, 2H) 4.41 - 4.47 (m, 1H) 5.71 - 5.76 (m, 1 H) 6.24 (dd, *J* = 17.05, 1.88 Hz, 1H) 6.44 (dd, *J* = 16.94, 10.16 Hz, 1H) 6.91 - 7.02 (m, 2H) 7.22 - 7.29 (m, 2H) 7.49 (d, *J* = 7.96 Hz, 1H) 7.70 - 7.78 (m, 2H) 7.89 (d, *J* = 7.96 Hz, 2 H) 10.10 (s, 1H) 10.89 (s, 1H); **^{13}C-NMR** (176 MHz, DMSO-*d_6*) δ ppm 26.23 (s, 2C) 47.52 (s, 2C) 51.08 (s, 1C) 59.29 (s, 1C) 62.92 (s, 1C) 101.22 (s, 1C) 108.58 (s, 1C) 113.34 (s, 1C) 124.69 (s, 1C) 125.37 (s, 1C) 128.89 (s, 1C) 130.32 (s, 1C) 131.13 (s, 1C) 132.29 (s, 1C) 133.97 (s, 1C) 134.50 (s, 1C) 140.08 (s, 1C) 144.39 (s, 1C) 153.81 (s, 1C) 162.87 (s, 1C); **HPLC-MS (ESI):** [R_t]: 4.57 min, *m/z* for $C_{22}H_{26}BN_4O_4$ ([M+H$^+$]), 421.28 cacld., 421.27 found.

Synthesis of methyl 5-bromo-6-chloronicotinate (**S6**).

To a solution of 5-bromo-6-chloronicotinic acid (3.0 g, 12.6 mmol, 1.0 eq.) in DMF (25 mL, 0.5 mmol/mL) 2.5 eq. of K_2CO_3 (4.3 g, 31.7 mmol) were added, then 1.2 eq. of methyliodide (2.16 g, 0.98 mL, 15.2 mmol) were added as well. The mixture was stirred at room temperature overnight. Afterwards the solvent was removed under reduced pressure and the solid dissolved with DCM, then everything was washed with saturated NaCl solution. The combined organic fractions were dried over Na_2SO_4 and the solvent was evaporated *in vacuo*. The pure product **S6** (2,2 g, 9.0 mmol, 71 %) was obtained after silica gel column chromatography (5 – 10 % EtOAc/PE). **^{1}H-NMR** (600 MHz, DMSO-d_6) δ ppm 3.90 (s, 3 H) 8.60 (s, 1 H) 8.89 (s, 1 H); **^{13}C-NMR** (151 MHz, DMSO-d_6) δ ppm 52.88 (s, 1 C) 119.75 (s, 1 C) 126.38 (s, 1 C) 142.90 (s, 1 C) 148.72 (s, 1 C) 153.47 (s, 1 C) 163.36 (s, 1 C); **HPLC-MS (ESI):** [R_t]: 9.61 min, *m/z* for $C_7H_5BrClNO_2$ ([M+H$^+$]), 250.48 cacld., 250.12 found.

Synthesis of (5-bromo-6-chloropyridin-3-yl)methanol (**S7**).

To a solution of 5-bromo-6-chloronicotinic acid (1.0 g, 4.2 mmol, 1.0 eq.) in THF (15 mL) at 0 °C was 1 M borane solution (12.7 mL, 12.7 mmol, 3.0 eq.) added dropwise and stirred for 30 min. After stirring for 2 hours at room temperature the reaction was stopped by addition of water (30 mL). Ethylacetate was added and the combined organic fractions were washed with $NaHCO_3$ solution, then dried over Na_2SO_4. The solvent was removed under reduced pressure and the desired product **S7** (410.0 mg, 1.8 mmol, 44 %) was obtained after silica gel column chromatography (10 % EtOAc/PE). **^{1}H-NMR** (500 MHz, DMSO-d_6) δ ppm 4.54 (d, *J* = 5.65 Hz, 2 H) 8.15 (s, 1 H) 8.36 (s, 1 H); **^{13}C-NMR** (151 MHz, DMSO-d_6) δ ppm 59.64 (s, 1C) 119.41 (s, 1C) 139.89 (s, 1C) 141.44 (s, 1C) 147.16 (s, 1C) 147.87 (s, 1C); **HPLC-MS (ESI):** *m/z* for $C_6H_5BrClNO$ ([M+H$^+$]), 222.47 cacld., 222.60 found.

Synthesis of 2-chloro-3-phenylpyridine (**S8**).

3-Bromo-2-chloropyridine (50.0 mg, 0.26 mmol, 1.0 eq.), phenylboronic acid (63.6 mg, 0.31 mmol, 1.2 eq.), K_2CO_3 (107.7 mg, 0.77 mmol, 3.0 eq.) and [1,1'-bis(diphenylphosphino)ferrocene]palladium(II)dichloride (19.1 mg, 0.026 mol, 0.1 eq.) were used following common procedure A and yielded the titled compound **S8** as a solid (34.0 mg, 0.18 mmol, 69 %). **^{1}H-NMR** (700 MHz, DMSO-d_6) δ ppm 7.41 - 7.56 (m, 6 H) 7.88 (d, *J* = 7.53 Hz, 4 H) 8.44 (d, *J* = 4.73 Hz, 4 H); **^{13}C-NMR** (176 MHz, DMSO-d_6) δ ppm 123.44 (s, 1 C) 128.28 (s, 1 C) 128.37 (s, 1 C) 129.18 (s, 1 C) 136.21 (s, 1 C) 137.04 (s, 1 C) 140.31 (s, 1 C) 148.34 (s, 1 C) 148.68 (s, 1 C); **HPLC-MS (ESI):** [R_t]: 9.16 min, *m/z* for $C_{11}H_9ClN$ ([M+H$^+$]), 190.64 cacld., 190.12 found.

Synthesis of 2-chloro-5-methyl-3-phenylpyridine (**S9**).

3-Bromo-2-chloro-5-metyhlpyridine (2.0 g, 9.6 mmol, 1.0 eq.), phenylboronic acid (1.2 g, 9.6 mmol, 1.0 eq.), K_3PO_4 (4.1 g, 19.3 mmol, 2.0 eq.) and [1,1'-bis(diphenylphosphino)ferrocene]palladium(II)dichloride (328 mg, 0.45 mol, 0.05 eq.) were used following common procedure A and yielded the titled compound **S9** as a solid (1.8 g, 9.1 mmol, 94 %). **^{1}H-NMR** (600 MHz, DMSO-d_6) δ ppm 2.33 (s, 3 H) 7.37 - 7.54 (m, 5 H) 7.70 (s, 1 H) 8.26 (s, 1 H); **^{13}C-NMR** (151 MHz, DMSO-d_6) δ ppm 16.93 (s, 1 C) 128.21 (s, 1 C) 128.34 (s, 1 C) 129.17 (s, 1 C) 133.06 (s, 1 C) 135.44 (s, 1 C) 137.10 (s, 1 C) 140.78 (s, 1 C) 145.51 (s, 1 C) 148.62 (s, 1 C); **HPLC-MS (ESI):** *m/z* for $C_{12}H_{11}ClN$ ([M+H$^+$]), 204.67 cacld., 204.15 found.

Synthesis of methyl 6-chloro-5-phenylnicotinate (**S10**).

S6 (1.0 g, 3.9 mmol, 1.0 eq.), phenylboronic acid (486 mg, 3.9 mmol, 1.0 eq.), K_3PO_4 (1.6 g, 7.9 mmol, 2.0 eq.) and [1,1'-bis(diphenylphosphino)ferrocene]palladium(II)dichloride (142 mg, 0.19 mmol, 0.05 eq.) were used following common procedure A and yielded the titled compound **S10** as a solid (730 mg, 2.9 mmol, 73 %). **^{1}H-NMR** (700 MHz, DMSO-d_6) δ ppm 3.91 (s, 3 H) 7.46 - 7.55 (m, 5 H) 8.21 (d, *J* = 2.37 Hz, 1 H) 8.91 (d, *J* = 2.37 Hz, 1 H); **^{13}C-NMR** (176 MHz, DMSO-d_6) δ ppm 52.68 (s, 1 C) 125.35 (s, 1 C) 128.52 (s, 2 C) 128.75 (s, 1 C) 129.17 (s, 2 C) 136.03 (s, 1 C) 136.23 (s, 1 C) 140.18 (s, 1 C) 148.91 (s, 1 C) 152.49 (s, 1 C) 164.31 (s, 1 C); **HPLC-MS (ESI):** *m/z* for $C_{13}H_{11}ClNO_2$ ([M+H$^+$]), 248.68 cacld., 248.12 found.

Synthesis of (6-chloro-5-phenylpyridin-3-yl)methanol (**S11**).

S7 (150 mg, 0.67 mmol, 1.0 eq.), phenylboronic acid (82.1 mg, 0.67 mmol, 1.0 eq.), K_3PO_4 (285 mg, 1.3 mmol, 2.0 eq.) and [1,1'-bis(diphenylphosphino)ferrocene]palladium(II)dichloride (43.8 mg, 0.06 mol, 0.1 eq.) were used following common procedure A and yielded the titled compound **S11** as a solid (110 mg, 0.5 mmol, 75 %). **^{1}H-NMR** (700 MHz, DMSO-d_6) δ ppm 4.57 (dd, *J*= 36.35, 6.02 Hz, 2 H) 5.41 - 5.52 (m, 1 H) 7.42 - 7.53 (m, 5 H) 7.77 (s, 1 H) 8.37 (s, 1 H); **^{13}C-NMR** (176 MHz, DMSO-d_6) δ ppm 59.96 (s, 1C) 128.77 (s, 2C) 128.89 (s, 1C) 129.62 (s, 2C) 138.16 (s, 1C) 139.00 (s, 1C) 139.89 (s, 1C) 141.43 (s, 1C) 147.15 (s, 1C) 147.26 (s, 1C); **HPLC-MS (ESI):** *m/z* for $C_{12}H_{10}ClNO$ ([M+H$^+$]), 220.67 cacld., 220.15 found.

Synthesis of 5-(bromomethyl)-2-chloro-3-phenylpyridine (**S12**).

S9 (1.0 g, 4.9 mmol, 1.0 eq.) was dissolved in 25 mL tetrachlormethane, then AIBN (80.3 mg, 0.49 mmol, 0.1 eq.) and N-bromosuccinimide (961 mg, 5.4 mmol, 1.1 eq.) were added. The reaction mixture was heated under reflux to 90 °C and stirred for 1 h. Afterwards the succcinimide was filtered off and the solvent was evaporated under reduced pressure. Then the solid was dissolved with DCM, and everything washed with saturated NaCl solution. The combined organic fractions were dried over Na_2SO_4 and the solvent was evaporated *in vacuo*. The pure product **S12** (602 mg, 2.3 mmol, 44 %) was obtained after silica gel column chromatography (3-4 % EtOAc/PE). **^{1}H-NMR** (600 MHz, DMSO-d_6) δ ppm 4.79 (s, 2H) 7.40 - 7.54 (m, 5H) 7.96 - 7.99 (m, 1H) 8.51 - 8.54 (m, 1H); **^{13}C-NMR** (151 MHz, DMSO-d_6) δ ppm 29.34 (s, 1C) 66.19 (s, 1C) 128.48 (s, 1C) 129.15 (s, 1C) 134.20 (s, 1C) 136.05 (s, 1C) 136.53 (s, 1C) 140.84 (s, 1C) 147.89 (s, 1C) 148.79 (s, 1C); **HPLC-MS (ESI):** *m/z* for $C_{12}H_{10}BrClN$ ([M+H$^+$]), 283.57 cacld., 284.01 found.

Synthesis of 2-chloro-3-phenyl-5-(pyrrolidin-1-ylmethyl)pyridine (**S13**).

S12 (50.0 mg, 0.17 mmol, 1.0 eq.), 3.0 eq. K_2CO_3 (73.4 mg, 0.53 mmol) and the nucleophile (25.1 mg, 0.35 mmol, 2.0 eq.) were used following common procedure C and yielded the titled compound as a solid (35.0 mg, 0.12 mmol, 64 %). **^{1}H-NMR** (500 MHz, DMSO-*d*$_6$) *δ* ppm 1.70 (dt, J=6.65, 3.48 Hz, 4 H) 2.47 (t, J=6.46 Hz, 4 H) 3.66 (s, 2 H) 7.43 - 7.51 (m, 5 H) 7.76 (s, 1 H) 8.35 (s, 1 H); **^{13}C-NMR** (126 MHz, DMSO-*d*$_6$) *δ* ppm 23.61 (s, 2C) 53.86 (s, 2C) 56.00 (s, 1C) 128.88 (s, 2C) 129.70 (s, 2C) 135.32 (s, 1C) 136.09 (s, 1C) 137.50 (s, 1C) 140.73 (s, 1C) 147.17 (s, 1C) 148.83 (s, 1C); **HPLC-MS (ESI):** *m/z* for $C_{16}H_{18}ClN_2$ ([M+H$^+$]), 273.78 cacld., 272.40 found.

Synthesis of 5-((1H-imidazol-1-yl)methyl)-2-chloro-3-phenylpyridine (**S14**).

S12 (50.0 mg, 0.17 mmol, 1.0 eq.), 3.0 eq. K_2CO_3 (73.4 mg, 0.53 mmol) and the nucleophile (23.8 mg, 0.35 mmol, 2.0 eq.) were used following common procedure C and yielded the titled compound as a solid (29.5 mg, 0.11 mmol, 69 %). **^{1}H-NMR** (600 MHz, DMSO-*d*$_6$) *δ* ppm 5.30 (s, 2 H) 6.91 (t, *J* = 1.15 Hz, 1 H) 7.30 (t, *J* = 1.15 Hz, 1 H) 7.48 (ddd, *J* = 22.20, 14.60, 6.90 Hz, 5 H) 7.81 (d, *J* = 2.34 Hz, 1 H) 7.82 (s, 1 H) 8.41 (s, 1 H); **^{13}C-NMR** (151 MHz, DMSO-*d*$_6$) *δ* ppm 46.46 (s, 1 C) 119.92 (s, 1C) 128,92 (s, 2C) 129.51 (s, 1C) 129.65 (s, 2C) 133.86 (s, 1C) 137.15 (s, 2C) 137.91 (s, 1C) 140.16 (s, 2 C); **HPLC-MS (ESI):** *m/z* for $C_{15}H_{13}ClN_3$ ([M+H$^+$]), 270.73 cacld., 269.20 found.

Synthesis of 2-chloro-5-((1-methyl-1H-pyrazol-4-yl)methyl)-3-phenylpyridine (**S15**).

S12 (50.0 mg, 0.17 mmol, 1.0 eq.), boronic acid (22.3 mg, 0.17 mmol, 1.0 eq.), K_3PO_4 (75.1 mg, 0.35 mmol, 2.0 eq.) and [1,1'-bis(diphenylphosphino)ferrocene]palladium(II)dichloride (6.21 mg, 0.03 mmol, 0.05 eq.) were used following common procedure B and yielded the titled compound as a solid (42.5 mg, 0.14 mmol, 85 %). **^{1}H-NMR** (600 MHz, DMSO-*d*$_6$) *δ* ppm3.75 (s, 3 H) 3.83 (s, 2 H) 7.32 (s, 1 H) 7.41 - 7.51 (m, 5 H) 7.53 (s, 1 H) 7.69 (d, *J* = 2.20 Hz, 1 H) 8.31 (d, *J* = 2.20 Hz, 1 H); **^{13}C-NMR** (126 MHz, MeOD-*d*$_4$) *δ* ppm 25.99 (s, 1 C) 38.38 (s, 1 C) 118.72 (s, 1 C) 128.27 (s, 1 C) 128.36 (s, 2 C) 129.19 (s, 2 C) 129.32 (s, 1 C) 135.68 (s, 1 C) 137.04 (s, 1 C) 137.20 (s, 1 C) 138.07 (s, 1 C) 140.08 (s, 1 C) 145.89 (s, 1 C) 148.22 (s, 1 C); **HPLC-MS (ESI):** [R$_t$]: 8.84 min, *m/z* for $C_{16}H_{15}ClN_3$ ([M+H$^+$]), 284.76 cacld., 284.17 found.

Synthesis of 5-((6-chloro-5-phenylpyridin-3-yl)methyl)pyridin-2-amine (**S16**).

S12 (50.0 mg, 0.17 mmol, 1.0 eq.), boronic ester (38.9 mg, 0.17 mmol, 1.0 eq.), K_3PO_4 (75.1 mg, 0.35 mmol, 2.0 eq.) and [1,1'-bis(diphenylphosphino)ferrocene]palladium(II)dichloride (6.21 mg, 0.03 mmol, 0.05 eq.) were used following common procedure B and yielded the titled compound as a solid (27.6 mg, 0.09 mmol, 55 %). **^{1}H-NMR** (600 MHz, DMSO-*d*$_6$) *δ* ppm 3.94 (s, 2 H) 6.93 (d, *J* = 8.99 Hz, 1 H) 7.41 - 7.54 (m, 5 H) 7.81 (d, *J* = 2.20 Hz, 1 H) 7.86 - 7.90 (m, 1 H) 7.93 (s, 1 H) 7.99 (s, 2 H) 8.38 (s, 1 H); **^{13}C-NMR** (126 MHz, MeOD-*d*$_4$) *δ* ppm 32.71 (s, 1 C) 114.27 (s, 1 C) 117.62 (s, 1 C) 124.48 (s, 1 C) 128.86 (s, 2 C) 129.72 (s, 2 C) 134.70 (s, 1 C) 135.82 (s, 1 C) 136.37 (s, 1 C) 137.35 (s, 1 C) 141.03 (s, 1 C) 145.65 (s, 1 C) 147.09 (s, 1 C) 149.03 (s, 1 C) 153.42 (s, 1 C); **HPLC-MS (ESI):** [R$_t$]: 6.04 min, *m/z* for $C_{17}H_{15}ClN_3$ ([M+H$^+$]), 296.76 cacld., 296.23 found.

Synthesis of 2-chloro-5-((5-methylfuran-2-yl)methyl)-3-phenylpyridine (**S17**).

S12 (50.0 mg, 0.17 mmol, 1.0 eq.), boronic ester (36.8 mg, 0.17 mmol, 1.0 eq.), K_3PO_4 (75.1 mg, 0.35 mmol, 2.0 eq.) and [1,1'-bis(diphenylphosphino)ferrocene]palladium(II)dichloride (6.21 mg, 0.03 mmol, 0.05 eq.) were used following common procedure B and yielded the titled compound as a solid (33.5 mg, 0.11 mmol, 67 %). **^{1}H-NMR** (700 MHz, $CDCl_3$-*d*) *δ* ppm 2.25 (s, 3 H) 3.96 (s, 2 H) 5.88 (s, 1 H) 5.96 (s, 1 H) 7.41 - 7.49 (m, 5 H) 7.58 (s, 1 H) 8.32 (s, 1 H); **^{13}C-NMR** (176 MHz, $CDCl_3$-*d*) *δ* ppm 13.51 (s, 1 C) 31.04 (s, 1 C) 106.20 (s, 1 C) 107.74 (s, 1 C) 128.33 (s, 2 C) 128.37 (s, 1 C) 129.33 (s, 2 C) 133.51 (s, 1 C) 136.71 (s, 1 C) 137.20 (s, 1 C) 140.37 (s, 1 C) 147.51 (s, 1 C) 148.08 (s, 1 C) 150.30 (s, 1 C) 151.78 (s, 1 C); **HPLC-MS (ESI):** [R$_t$]: 10.91 min, *m/z* for $C_{17}H_{15}ClNO$ ([M+H$^+$]), 284.76 cacld., 284.17 found.

Synthesis of 3-((6-chloro-5-phenylpyridin-3-yl)methyl)phenol (**S18**).

S12 (50.0 mg, 0.17 mmol, 1.0 eq.), boronic acid (24.4 mg, 0.17 mmol, 1.0 eq.), K_3PO_4 (75.1 mg, 0.35 mmol, 2.0 eq.) and [1,1'-bis(diphenylphosphino)ferrocene]palladium(II)dichloride (6.21 mg, 0.03 mmol, 0.05 eq.) were used following common procedure B and yielded the titled compound as a solid (29.0 mg, 0.09 mmol, 58 %). **^{1}H-NMR** (600 MHz, DMSO-*d*$_6$) *δ* ppm 3.93 (s, 2 H) 6.60 (d, *J* = 8.07 Hz, 1 H) 6.65 (s, 1 H) 6.71 (d, *J* = 7.34 Hz, 1 H) 7.09 (t, *J* =7 .79 Hz, 1 H) 7.45 (s, 5 H) 7.72 (d, *J* = 2.20 Hz, 1 H) 8.33 (s, 1 H) 9.32 (s, 1 H); **^{13}C-NMR** (151 MHz, DMSO-*d*$_6$) *δ* ppm 36.89 (s, 1 C) 113.35 (s, 1 C) 115.49 (s, 1 C) 119.28 (s, 1 C) 128.30 (s, 1 C) 128.39 (s, 2 C) 129.17 (s, 2 C) 129.62 (s, 1 C) 135.71 (s, 1 C) 136.86 (s, 1 C) 136.98 (s, 1 C) 140.37 (s, 1 C) 141.34 (s, 1 C) 146.06 (s, 1 C) 148.48 (s, 1 C) 157.52 (s, 1 C); **HPLC-MS (ESI):** [R$_t$]: 9.57 min, *m/z* for $C_{18}H_{16}ClNO$ ([M+H$^+$]), 296.77 cacld., 296.18 found.

Synthesis of 4-((6-chloro-5-phenylpyridin-3-yl)methyl)aniline (**S19**).

S12 (50.0 mg, 0.17 mmol, 1.0 eq.), boronic ester (38.7 mg, 0.17 mmol, 1.0 eq.), K_3PO_4 (75.1 mg, 0.35 mmol, 2.0 eq.) and [1,1'-bis(diphenylphosphino)ferrocene]palladium(II)dichloride (6.21 mg, 0.03 mmol, 0.05 eq.) were used following common procedure B and yielded the titled compound as a solid (38.9 mg, 0.13 mmol, 78 %). **^{1}H-NMR** (500 MHz, MeOD-*d*$_4$) *δ* ppm 4.07 (s, 2H) 5.51 - 5.85 (m, 2 H) 7.10 (d, *J* = 7.89 Hz, 1H) 7.12 (s, 1H) 7.28 (d, *J* = 7.70 Hz, 1H) 7.36 - 7.40 (m, 1H) 7.43 - 7.51 (m, 5H) 7.77 (d, *J* = 2.20 Hz, 1H) 8.37 (d, *J* = 2.38 Hz, 1H); **^{13}C-NMR** (126 MHz, MeOD-*d*$_4$) *δ* ppm 36.50 (s, 1C) 112.71 (s, 1C) 114.63 (s, 1C) 116.55 (s, 1C) 118.42 (s, 1C) 120.00 (s, 1C) 121.87 (s, 1C) 127.01 (s, 1C) 128.09 - 128.77 (m, 1C) 129.19 (s, 1C) 130.05 (s, 1C) 135.87 (s, 1C) 136.27 (s, 1C) 136.89 (s, 1C) 140.51 (s, 1C) 141.84 (s, 1C) 146.30 (s, 1C) 148.62 (s, 1C); **HPLC-MS (ESI):** *m/z* for $C_{18}H_{16}ClN_2$ ([M+H$^+$]), 295.78 cacld., 295.15 found.

Synthesis of 3-((6-chloro-5-phenylpyridin-3-yl)methyl)aniline (**S20**).
5-(Bromomethyl)-2-chloro-3-phenylpyridine (**S12**, 50.0 mg, 0.17 mmol, 1.0 eq.), boronic ester (38.7 mg, 0.17 mmol, 1.0 eq.), K_3PO_4 (75.1 mg, 0.35 mmol, 2.0 eq.) and [1,1'-bis(diphenylphosphino)ferrocene]palladium(II)dichloride (6.21 mg, 0.03 mmol, 0.05 eq.) were used following common procedure B and yielded the titled compound as a solid (26.5 mg, 0.09 mmol, 52 %). **^{1}H-NMR** (600 MHz, DMSO-d_6) δ ppm 4.07 (s, 2H) 5.51 - 5.85 (m, 2 H) 7.10 (d, J = 7.89 Hz, 1H) 7.12 (s, 1H) 7.28 (d, J = 7.70 Hz, 1H) 7.36 - 7.40 (m, 1H) 7.43 - 7.51 (m, 5H) 7.77 (d, J = 2.20 Hz, 1H) 8.37 (d, J = 2.38 Hz, 1H); **^{13}C-NMR** (151 MHz, DMSO-d_6) δ ppm 36.50 (s, 1C) 112.71 (s, 1C) 114.63 (s, 1C) 116.55 (s, 1C) 118.42 (s, 1C) 120.00 (s, 1C) 121.87 (s, 1C) 127.01 (s, 1C) 128.09 - 128.77 (m, 1C) 129.19 (s, 1C) 130.05 (s, 1C) 135.87 (s, 1C) 136.27 (s, 1C) 136.89 (s, 1C) 140.51 (s, 1C) 141.84 (s, 1C) 146.30 (s, 1C) 148.62 (s, 1C); **HPLC-MS (ESI):** [R$_t$]: 8.14 min, *m/z* for $C_{18}H_{16}ClN_2$ ([M+H$^+$]), 295.78 cacld., 295.22 found.

Synthesis of 2-chloro-3-phenyl-5-(pyridin-3-ylmethyl)pyridine (**S21**).
S12 (50.0 mg, 0.17 mmol, 1.0 eq.), boronic ester (21.7 mg, 0.17 mmol, 1.0 eq.), K_3PO_4 (75.1 mg, 0.35 mmol, 2.0 eq.) and [1,1'-bis(diphenylphosphino)ferrocene]palladium(II)dichloride (6.21 mg, 0.03 mmol, 0.05 eq.) were used following common procedure B and yielded the titled compound as a solid (41.0 mg, 0.13 mmol, 81 %). **^{1}H-NMR** (700 MHz, MeOD-d_4) δ ppm 4.12 (s, 2H) 7.38 - 7.43 (m, 4H) 7.44 (s, 1H) 7.44 - 7.47 (m, 1H) 7.69 (d, J = 2.47 Hz, 1H) 7.75 (d, J = 7.90 Hz, 1H) 8.29 (d, J = 2.47 Hz, 1H) 8.42 (dd, J = 4.95, 1.51 Hz, 1H) 8.51 (d, J = 1.72 Hz, 1H); **^{13}C-NMR** (156 MHz, MeOD-d_4) δ ppm 35.82 (s, 1C) 125.65 (s, 1C) 129.41 (s, 2C) 129.67 (s, 1C) 130.47 (s, 2C) 137.37 (s, 1C) 137.83 (s, 1C) 138.63 (s, 1C) 138.69 (s, 1C) 138.84 (s, 1C) 142.04 (s, 1C) 148.59 (s, 1C) 148.76 (s, 1C) 149.43 (s, 1C) 150.49 (s, 1C); **HPLC-MS (ESI):** [R$_t$]: 6. 43min, *m/z* for $C_{17}H_{14}ClN_2$ ([M+H$^+$]), 281.75 cacld., 281.22 found.

Synthesis of 2-chloro-5-((pyrazol-4-yl)methyl)-3-phenylpyridine (**S22**).
S12 (50.0 mg, 0.17 mmol, 1.0 eq.), boronic ester (19.8 mg, 0.17 mmol, 1.0 eq.), K_3PO_4 (75.1 mg, 0.35 mmol, 2.0 eq.) and [1,1'-bis(diphenylphosphino)ferrocene]palladium(II)dichloride (6.21 mg, 0.03 mmol, 0.05 eq.) were used following common procedure B and yielded the titled compound as a solid (34.2 mg, 0.12 mmol, 70 %). **^{1}H-NMR** (500 MHz, MeOD-d_4) δ ppm 5.46 (s, 2H) 6.36 (t, J = 2.14 Hz, 1H) 7.36 - 7.48 (m, 5H) 7.56 (d, J = 1.83 Hz, 1H) 7.64 (d, J = 2.29 Hz, 1H) 7.81 (d, J = 2.44 Hz, 1H) 8.24 (d, J = 2.44 Hz, 1H); **^{13}C-NMR** (126 MHz, MeOD-d_4) δ ppm 52.84 (s, 1C) 107.50 (s, 1C) 129.36 (s, 1C) 129.76 (s, 2C) 130.30 (s, 2C) 132.13 (s, 1C) 134.46 (s, 1C) 138.51 (s, 1C) 138.56 (s, 1C) 140.88 (s, 1C) 141.41 (s, 1C) 148.40 (s, 1C) 149.96 (s, 1C); **HPLC-MS (ESI):** [R$_t$]: 8.25 min, *m/z* for $C_{15}H_{13}ClN_3$([M+H$^+$]), 270.73 cacld., 270.12 found.

Synthesis of 6-chloro-5-phenylnicotinic acid (**S23**).
S10 (200 mg, 0.8 mmol, 1.0 eq.) were dissolved in THF and MeOH (1:1, 10 mL/mmol) and then 2 mL conc. NaOH solution was added. The resulting mixture was stirred at rt for 2 h. Afterwards DCM was added and the pH was adjusted to 1. The combined organic phases were washed with 1 N HCl and then dried over Na_2SO_4, filtrated and the solvent was removed under reduced pressure. Without further purification the titled compound was yielded as white solid (168 mg, 0.72 mmol, 90 %). **^{1}H-NMR** (700 MHz, DMSO-d_6) δ ppm 7.41 - 7.59 (m, 5 H) 8.18 (d, J = 2.26 Hz, 1 H) 8.89 (d, J = 2.26 Hz, 1 H) 13.71 (s, 1 H); **^{13}C-NMR** (176 MHz, DMSO-d_6) δ ppm 126.40 (s, 1 C) 128.51 (s, 2 C) 128.68 (s, 1 C) 129.18 (s, 2 C) 136.10 (s, 1 C) 136.21 (s, 1 C) 140.34 (s, 1 C) 149.16 (s, 1 C) 152.13 (s, 1 C) 165.29 (s, 1 C); **HPLC-MS (ESI):** [R$_t$]: 8.28 min, *m/z* for $C_{12}H_9ClNO_2$ ([M+H$^+$]), 234.65 cacld., 234.12 found.

Synthesis of 6-chloro-N-methyl-5-phenylnicotinamide (**S24**).
S23 (50.0 mg, 0.21 mmol, 1.0 eq.), amine (14.4 mg, 0.21 mmol, 1.0 eq.), HATU (121 mg, 0.32 mmol, 1.5 eq.) and DIPEA (0.22 mL, 1.3 mmol, 6.0 eq.) were used following common procedure D and yielded the titled compound as a solid (30.1 mg, 0.12 mmol, 60 %). **^{1}H-NMR** (500 MHz, DMSO-d_6) δ ppm 2.78 - 2.83 (m, 3 H) 7.43 - 7.58 (m, 5 H) 8.23 (d, J = 2.44 Hz, 1 H) 8.76 (d, J = 4.42 Hz, 1 H) 8.82 (d, J = 2.44 Hz, 1 H); **^{13}C-NMR** (126 MHz, DMSO-d_6) δ ppm 26.25 (s, 1 C) 128.50 (s, 2 C) 128.64 (s, 1 C) 129.30 (s, 2 C) 129.45 (s, 1 C) 135.65 (s, 1 C) 136.50 (s, 1 C) 138.61 (s, 1 C) 147.54 (s, 1 C) 150.40 (s, 1 C) 163.89 (s, 1 C); **HPLC-MS (ESI):** *m/z* for $C_{13}H_{12}ClN_2O$ ([M+H$^+$]), 247.69 cacld., 257.15 found.

Synthesis of 6-chloro-N-isobutyl-5-phenylnicotinamide (**S25**).
S23 (50.0 mg, 0.21 mmol, 1.0 eq.), amine (17.2 mg, 0.21 mmol, 1.0 eq.), HATU (121 mg, 0.32 mmol, 1.5 eq.) and DIPEA (0.22 mL, 1.3 mmol, 6.0 eq.) were used following common procedure D and yielded the titled compound as a solid (31.0 mg, 0.10 mmol, 51 %). **^{1}H-NMR** (500 MHz, DMSO-d_6) δ ppm 0.85 - 0.94 (m, 6 H) 1.84 (dt, J = 13.50, 6.83 Hz, 1 H) 3.10 (dd, J = 6.87, 5.80 Hz, 2 H) 7.43 - 7.61 (m, 5 H) 8.26 (d, J = 2.44 Hz, 1 H) 8.76 (t, J = 5.72 Hz, 1 H) 8.83 (d, J = 2.29 Hz, 1 H); **^{13}C-NMR** (126 MHz, DMSO-d_6) δ ppm 20.24 (s, 2 C) 28.05 (s, 1 C) 46.82 (s, 1 C) 128.49 (s, 2 C) 128.62 (s, 1 C) 129.32 (s, 2 C) 129.60 (s, 1 C) 135.62 (s, 1 C) 136.54 (s, 1 C) 138.64 (s, 1 C) 147.71 (s, 1 C) 150.39 (s, 1 C) 163.51 (s, 1 C); **HPLC-MS (ESI):** [R$_t$]: 9.03 min, *m/z* for $C_{16}H_{18}ClN_2O$ ([M+H$^+$]), 289.78 cacld., 289.13 found.

Synthesis of 6-chloro-N-(1-methyl-1H-pyrazol-4-yl)-5-phenylnicotinamide (**S26**).
S23 (50.0 mg, 0.21 mmol, 1.0 eq.), amine (20.7 mg, 0.21 mmol, 1.0 eq.), HATU (121 mg, 0.32 mmol, 1.5 eq.) and DIPEA (0.22 mL, 1.3 mmol, 6.0 eq.) were used following common procedure D and yielded the titled compound as a solid (15.5 mg, 0.05 mmol, 24 %). **^{1}H-NMR** (400 MHz, MeOD-d_4) δ ppm 3.93 (s, 3 H) 7.48 - 7.58 (m, 5 H) 7.67 (d, J = 0.49 Hz, 1 H) 7.86 (s, 1 H) 8.08 (s, 1 H) 8.34 (d, J = 2.45 Hz, 1 H) 8.93 (d, J = 2.45 Hz, 1 H); **^{13}C-NMR** (101 MHz, MeOD-d_4) δ ppm 37.98 (s, 1 C) 121.44 (s, 1 C) 122.57 (s, 1 C) 128.25 (s, 2 C) 128.51 (s, 1 C) 129.01 (s, 2 C) 129.39 (s, 1 C) 130.52 (s, 1 C) 136.63 (s, 1 C) 136.98 (s, 1 C) 138.81 (s, 1 C) 147.01 (s, 1 C) 151.77 (s, 1 C) 162.16 (s, 1 C); **HPLC-MS (ESI):** [R$_t$]: 6.70 min, *m/z* for $C_{16}H_{13}ClN_4O$ ([M+H$^+$]), 313.76 cacld., 313.10 found.

Synthesis of (6-chloro-5-phenylpyridin-3-yl)(pyrrolidin-1-yl)methanone (**S27**).
S23 (50.0 mg, 0.21 mmol, 1.0 eq.), amine (15.2 mg, 0.21 mmol, 1.0 eq.), HATU (121 mg, 0.32 mmol, 1.5 eq.) and DIPEA (0.22 mL, 1.3 mmol, 6.0 eq.) were used following common procedure D and yielded the titled compound as a solid (11.5 mg, 0.04 mmol, 19 %). **^{1}H-NMR** (500 MHz, $CDCl_3$-*d*) δ ppm 1.91 - 2.05 (m, 4 H) 3.53 (t, *J* = 6.48 Hz, 2 H) 3.68 (t, *J* = 6.87 Hz, 2 H) 7.42 - 7.53 (m, 5 H) 7.88 (d, *J* = 2.29 Hz, 1 H) 8.57 (d, *J* = 2.29 Hz, 1 H); **^{13}C-NMR** (126 MHz, $CDCl_3$-*d*) δ ppm 24.34 (s, 1 C) 26.43 (s, 1 C) 46.60 (s, 1 C) 49.66 (s, 1 C) 128.45 (s, 2 C) 128.67 (s, 1 C) 129.20 (s, 2 C) 131.81 (s, 1 C) 136.54 (s, 1 C) 136.84 (s, 1 C) 138.80 (s, 1 C) 146.49 (s, 1 C) 150.76 (s, 1 C) 165.93 (s, 1 C); **HPLC-MS (ESI):** *m/z* for $C_{16}H_{16}ClN_2O$ ([M+H$^+$]), 287.76 cacld., 287.07 found.

Synthesis of 6-chloro-5-phenyl-N-(pyridin-3-yl)nicotinamide (**S28**).
S23 (50.0 mg, 0.21 mmol, 1.0 eq.), amine (20.1 mg, 0.21 mmol, 1.0 eq.), HATU (121 mg, 0.32 mmol, 1.5 eq.) and DIPEA (0.22 mL, 1.3 mmol, 6.0 eq.) were used following common procedure D and yielded the titled compound as a solid (48.4 mg, 0.15 mmol, 74 %). **^{1}H-NMR** (600 MHz, DMSO-d_6) δ ppm7.42 - 7.63 (m, 6H) 8.33 (d, *J* = 8.44 Hz, 1H) 8.41 - 8.48 (m, 2H) 8.97 (d, *J* = 2.38 Hz, 1H) 9.05 (d, *J* = 2.20 Hz, 1H) 10.88 (s, 1H); **^{13}C-NMR** (151 MHz, DMSO-d_6) δ ppm 124.75 (s, 1C) 128.40 (s, 2C) 128.74 (s, 1C) 129.17 (s, 2C) 129.21 (s, 1C) 129.75 (s, 1C) 135.75 (s, 1C) 136.10 (s, 1C) 136.33 (s, 1C) 139.18 (s, 1C) 139.55 (s, 1C) 142.95 (s, 1C) 148.18 (s, 1C) 151.24 (s, 1C) 163.29 (s, 1C); **HPLC-MS (ESI):** [R$_t$]: 7.02 min, *m/z* for $C_{17}H_{13}ClN_3O$ ([M+H$^+$]), 310.76 cacld., 310.13 found.

Synthesis of 6-chloro-5-phenyl-N-(pyridin-2-yl)nicotinamide (**S29**).
S23 (50.0 mg, 0.21 mmol, 1.0 eq.), amine (20.1 mg, 0.21 mmol, 1.0 eq.), HATU (121 mg, 0.32 mmol, 1.5 eq.) and DIPEA (0.22 mL, 1.3 mmol, 6.0 eq.) were used following common procedure D and yielded the titled compound as a solid (52.5 mg, 0.18 mmol, 80 %). **^{1}H-NMR** (700 MHz, DMSO-d_6) δ ppm 7.16 - 7.24 (m, 1H) 7.47 - 7.51 (m, 1H) 7.54 (t, *J* = 7.58 Hz, 2H) 7.60 - 7.63 (m, 2H) 7.85 - 7.89 (m, 1H) 8.20 (d, *J* = 8.28 Hz, 1H) 8.39 - 8.41 (m, 1H) 8.45 (d, *J* = 2.37 Hz, 1H) 8.95 (d, *J* = 2.37 Hz, 1H) 11.24 (s, 1H); **^{13}C-NMR** (151 MHz, DMSO-d_6) δ ppm 114.66 (s, 1C) 120.18 (s, 1C) 128.42 (s, 2C) 128.61 (s, 1C) 129.36 (s, 2C) 129.48 (s, 1C) 135.42 (s, 1C) 136.35 (s, 1C) 138.29 (s, 1C) 139.56 (s, 1C) 148.03 (s, 1C) 148.23 (s, 1C) 150.94 (s, 1C) 151.84 (s, 1C) 163.49 (s, 1C); **HPLC-MS (ESI):** [R$_t$]: 9.10 min, *m/z* for $C_{17}H_{13}ClN_3O$ ([M+H$^+$]), 310.76 cacld., 310.08 found.

Synthesis of 6-chloro-N-(3-nitrophenyl)-5-phenylnicotinamide (**S30a**).
S23 (50.0 mg, 0.21 mmol, 1.0 eq.), amine (29.5 mg, 0.21 mmol, 1.0 eq.), HATU (121 mg, 0.32 mmol, 1.5 eq.) and DIPEA (0.22 mL, 1.3 mmol, 6.0 eq.) were used following common procedure D and yielded the titled compound as a solid (31.0 mg, 0.09 mmol, 41 %). **^{1}H-NMR** (500 MHz, MeOD-d_4) δ ppm 7.43 - 7.55 (m, 5H) 7.59 (t, *J* = 8.24 Hz, 1H) 8.01 (ddd, *J* = 8.24, 2.29, 0.92 Hz, 1H) 8.08 (ddd, *J*= 8.16, 2.06, 0.92 Hz, 1H) 8.34 (d, *J* = 2.44 Hz, 1H) 8.74 (t, *J* = 2.14 Hz, 1H) 8.93 (d, *J* = 2.44 Hz, 1H); **^{13}C-NMR** (126 MHz, MeOD-d_4) δ ppm 116.41 (s, 1C) 120.09 (s, 1C) 127.37 (s, 1C) 129.54 (s, 2C) 129.83 (s, 1C) 130.29 (s, 2C) 130.83 (s, 1C) 130.91 (s, 1C) 137.75 (s, 1C) 138.25 (s, 1C) 140.22 (s, 1C) 140.79 (s, 1C) 148.32 (s, 1C) 149.74 (s, 1C) 153.42 (s, 1C) 165.34 (s, 1C); **HPLC-MS (ESI):** [R$_t$]: 10.07 min, *m/z* for $C_{18}H_{13}ClN_3O_3$ ([M+H$^+$]), 354.76 cacld., 354.16 found.

Synthesis of N-(3-aminophenyl)-6-chloro-5-phenylnicotinamide (**S30**).
S30a (50.0 mg, 0.14 mmol, 1.0 eq.), was solved in Ethanol/Water (5:1, 10 mL), then iron powder (39.5 mg, 0.7 mmol, 5.0 eq.), and NH_4Cl (0,37 g 7.0 mmol, 10 eq.) were added. The mixture was sonificated at rt for 30 min. The resulting precipitate was separated and the filtrate extracted with dichloromethane. The combined organic fractions were dried over Na_2SO_4 and the solvent was evaporated *in vacuo*. The pure product **S30** (29.0 mg, 0.09 mmol, 64 %) was obtained after silica gel column chromatography (3 % MeOH/DCM + 1 % NH_3). **^{1}H-NMR** (400 MHz, DMSO-d_6) δ ppm 6.89 (d, *J* = 7.34 Hz, 1H) 7.36 (d, *J* = 7.58 Hz, 1H) 7.39 - 7.46 (m, 1H) 7.47 - 7.62 (m, 5H) 7.75 (s, 1H) 8.38 (d, *J* = 2.45 Hz, 1H) 8.94 (d, *J* = 2.45 Hz, 1H) 10.58 (s, 1H); **^{13}C-NMR** (101 MHz, DMSO-d_6) δ ppm 128.53 (s, 2C) 128.70 (s, 1C) 129.32 (s, 2C) 129.85 (s, 1C) 135.63 (s, 1C) 136.40 (s, 1C) 139.13 (s, 1C) 139.65 (s, 2C) 148.08 (s, 1C) 150.88 (s, 1C) 162.89 (s, 1C); **HPLC-MS (ESI):** [R$_t$]: 7.60 min, *m/z* for $C_{18}H_{15}ClN_3O_1$ ([M+H$^+$]), 324.78 cacld., 324.14 found.

Synthesis of N-(2-oxo-3-(1-(4-(3-phenylpyridin-2-yl)benzyl)piperidin-4-yl)-2,3-dihydro-1H-benzo[d]imidazol-5-yl)acrylamide (**1**).
Chloropyridine (**S8**, 20.0 mg, 0.07 mmol, 1.0 eq.), (4-((4-(6-acrylamido-2-oxo-2,3-dihydro-1*H*-benzo[*d*]imidazol-1-yl)piperidin-1-yl)methyl)phenyl)boronic acid (29.4 mg, 0.07 mmol, 1.0 eq.), K_2CO_3 (19.4 mg, 0.14 mmol, 2.0 eq.) and tetrakis(triphenylphosphine)palladium(0) (8.0 mg, 0.007 mol, 0.1 eq.) were used following common procedure E and yielded the final compound as a solid (34.0 mg, 0.06 mmol, 91 %). **^{1}H-NMR** (700 MHz, MeOD-d_4) δ ppm 1.79 (d, *J* = 10.00 Hz, 2 H) 2.26 (t, *J* = 11.45 Hz, 2 H) 2.50 - 2.59 (m, 2 H) 3.09 (d, *J* = 11.83 Hz, 2 H) 3.64 (s, 2 H) 4.30 (s, 1 H) 5.80 (dd, *J* = 10.00, 1.72 Hz, 1 H) 6.35 - 6.41 (m, 1 H) 6.43 - 6.50 (m, 1 H) 7.04 (d, *J* = 8.39 Hz, 1 H) 7.19 (dd, *J* = 6.67, 2.90 Hz, 2 H) 7.25 - 7.37 (m, 8 H) 7.52 (dd, *J* = 7.74, 4.95 Hz, 1 H) 7.74 (d, *J* = 1.72 Hz, 1 H) 7.90 (dd, *J* = 7.74, 1.72 Hz, 1 H) 8.62 (dd, *J* = 4.89, 1.67 Hz, 1 H); **^{13}C-NMR** (176 MHz, MeOD-d_4) δ ppm 29.82 (s, 2 C) 52.40 (s, 1 C) 54.17 (s, 2 C) 63.35 (s, 1 C) 104.24 (s, 1 C) 110.53 (s, 1 C) 115.66 (s, 1 C) 124.13 (s, 1 C) 126.78 (s, 1 C) 127.71 (s, 1 C) 128.67 (s, 1 C) 129.55 (s, 2 C) 130.44 (s, 2 C) 130.68 (s, 1 C) 130.85 (s, 2 C) 131.15 (s, 1 C) 132.63 (s, 2 C) 133.84 (s, 1 C) 138.47 (s, 1 C) 140.52 (s, 1 C) 140.66 (s, 1 C) 140.95 (s, 1 C) 148.95 (s, 2 C) 156.78 (s, 1 C) 158.45 (s, 1 C) 166.24 (s, 1 C); **HRMS (ESI):** [R$_t$]: 5.98 min, *m/z* for $C_{33}H_{31}N_5O_2$ ([M+H$^+$]), 530.2550 cacld., 530.2557 found.

Synthesis of N-(3-(1-(4-(5-methyl-3-phenylpyridin-2-yl)benzyl)piperidin-4-yl)-2-oxo-2,3-dihydro-1H-benzo[d]imidazol-5-yl)acrylamide (**2**).
Chloropyridine (**S9**, 20.0 mg, 0.07 mmol, 1.0 eq.), (4-((4-(6-acrylamido-2-oxo-2,3-dihydro-1*H*-benzo[*d*]imidazol-1-yl)piperidin-1-yl)methyl)phenyl)boronic acid (29.4 mg, 0.07 mmol, 1.0 eq.), K_2CO_3 (19.4 mg, 0.14 mmol, 2.0 eq.) and tetrakis(triphenylphosphine)palladium(0) (8.0 mg, 0.007 mol, 0.1 eq.) were used following common procedure E and yielded the final

compound as a solid (27.0 mg, 0.05 mmol, 70 %).**^{1}H-NMR** (700 MHz, MeOD-*d_4*) *δ* ppm 2.14 (d, *J* = 13.44 Hz, 2 H) 2.60 (s, 3 H) 2.81 (d, *J* = 8.17 Hz, 2 H) 3.24 - 3.32 (m, 2 H) 3.64 (d, *J* = 10.65 Hz, 2 H) 4.43 (s, 2 H) 4.54 (t, *J* = 11.99 Hz, 1 H) 5.81 (d, *J* = 11.72 Hz, 1 H) 6.36 - 6.42 (m, 1 H) 6.43 - 6.52 (m, 1 H) 7.00 (d, *J* = 8.39 Hz, 1 H) 7.05 (d, *J* = 8.28 Hz, 1 H) 7.24 (d, *J* = 7.96 Hz, 2 H) 7.32 - 7.37 (m, 3 H) 7.50 - 7.54 (m, 2 H) 7.57 (d, *J* = 7.64 Hz, 2 H) 7.97 (s, 1 H) 8.18 (s, 1 H) 8.66 (s, 1 H) 10.08 (s, 1 H) 10.79 (s, 1 H); **^{13}C-NMR** (176 MHz, MeOD-*d_4*) *δ* ppm 18.16 (s, 1 C) 27.52 (s, 2 C) 49.68 (s, 1 C) 53.45 (s, 2 C) 61.32 (s, 1 C) 101.56 (s, 1 C) 103.48 (s, 1 C) 110.87 (s, 1 C) 115.05 (s, 1 C) 115.62 (s, 1 C) 116.69 (s, 1 C) 118.33 (s, 1 C) 126.70 (s, 1 C) 127.87 (s, 1 C) 129.65 (s, 1 C) 129.90 (s, 2 C) 130.45 (s, 1 C) 130.94 (s, 2 C) 132.17 (s, 1 C) 132.56 (s, 2 C) 134.09 (s, 1 C) 137.30 (s, 1 C) 138.64 (s, 1 C) 138.96 (s, 1 C) 139.94 (s, 1 C) 145.71 (s, 1 C) 151.99 (s, 1 C) 156.60 (s, 1 C) 166.18 (s, 1 C); **HRMS (ESI):** [R$_t$]: 5.85 min, *m/z* for $C_{34}H_{34}N_5O_2$ ([M+H$^+$]), 544.2634 cacld., 544.2696 found.

Synthesis of N-(3-(1-(4-(5-(hydroxymethyl)-3-phenylpyridin-2-yl)benzyl)piperidin-4-yl)-2-oxo-2,3-dihydro-1H-benzo[d]imidazol-5-yl)acrylamide (**3**).
Chloropyridine (**S11**, 20.0 mg, 0.09 mmol, 1.0 eq.), (4-((4-(6-acrylamido-2-oxo-2,3-dihydro-1*H*-benzo[*d*]imidazol-1-yl)piperidin-1-yl)methyl)phenyl)boronic acid (37.8 mg, 0.09 mmol, 1.0 eq.), K_2CO_3 (24.5 mg, 0.14 mmol, 2.0 eq.) and tetrakis(triphenylphosphine)palladium(0) (10.3 mg, 0.009 mol, 0.1 eq.) were used following common procedure E and yielded the final compound as a solid (18.1 mg, 0.03 mmol, 36 %). **^{1}H-NMR** (600 MHz, DMSO-*d_6*) *δ* ppm 2.51 - 2.62 (m, 1 H) 3.20 (m, 2 H) 3.88 (m, 4 H) 4.37 (m, 1 H) 4.67 (s, 4 H) 5.99 (dd, *J* = 301.42, 16.96 Hz, 2 H) 6.43 (dd, *J* = 16.99, 10.16 Hz, 1 H) 6.94 (s, 1 H) 7.20 (d, *J* = 7.79 Hz, 1 H) 7.22 - 7.30 (m, 1 H) 7.33 (m, 5 H) 7.41 (m, 3 H) 7.82 (d, *J* = 22.28 Hz, 1 H) 8.65 (s, 1 H); **^{13}C-NMR** (151 MHz, DMSO-*d_6*) *δ* ppm 25.35 (s, 2C) 47.01 (s, 1C) 50.52 (s, 2C) 58.35 (s, 1C) 59.67 (s, 2C) 61.90 (s, 1C) 100.86 (s, 1C) 108.32 (s, 1C) 112.74 (s, 1C) 124.09 (s, 1C) 125.28 (s, 1C) 127.94 (s, 2C) 128.78 (s, 2C) 128.98 (s, 2C) 129.55 (s, 2C) 130.29 (s, 2C) 131.34 (s, 1C) 131.83 (s, 1C) 134.72 (s, 1C) 138.69 (s, 1C) 145.99 (s, 1C) 153.40 (s, 1C) 162.29 (s, 1C); **HRMS (ESI):** *m/z* for $C_{34}H_{33}N_5O_3$ ([M+H$^+$]), 560.2656 cacld., 560.2654 found.

Synthesis of 6-(4-((4-(6-acrylamido-2-oxo-2,3-dihydro-1H-benzo[d]imidazol-1-yl)piperidin-1-yl)methyl)phenyl)-5-phenylnicotinic acid (**4**).
Chloropyridine (**S23**, 20.0 mg, 0.06 mmol, 1.0 eq.), (4-((4-(6-acrylamido-2-oxo-2,3-dihydro-1*H*-benzo[*d*]imidazol-1-yl)piperidin-1-yl)methyl)phenyl)boronic acid (28.2 mg, 0.06 mmol, 1.0 eq.), K_2CO_3 (15.5 mg, 0.12 mmol, 2.0 eq.) and tetrakis(triphenylphosphine)palladium(0) (6.9 mg, 0.006 mol, 0.1 eq.) were used following common procedure E and yielded the final compound as a solid (21.1 mg, 0.04 mmol, 70 %).**^{1}H-NMR** (700 MHz, DMSO-*d_6*) *δ* ppm 1.96 (d, *J* = 12.58 Hz, 2H) 2.62 (d, *J* = 12.69 Hz, 2H) 3.20 (d, *J* = 11.29 Hz, 2H) 3.46 (d, *J* = 11.08 Hz, 2H) 4.33 (d, *J* = 2.47 Hz, 2H) 4.37 (s, 1H) 5.74 (d, *J* = 10.11 Hz, 1H) 6.24 (d, *J* = 16.99 Hz, 1H) 6.39 - 6.46 (m, 1H) 6.93 - 6.93 (m, 1H) 6.95 (s, 2H) 7.24 - 7.28 (m, 1H) 7.35 (s, 2H) 7.45 (s, 2H) 7.49 - 7.75 (m, 2H) 7.81 - 7.92 (m, 1H) 8.23 (s, 1H) 9.16 (s, 1H) 9.71 - 9.79 (m, 1H) 10.08 (s, 1H) 10.88 (s, 1H); **^{13}C-NMR** (176 MHz, DMSO-*d_6*) *δ* ppm 25.91 (s, 2C) 47.54 (s, 1C) 51.10 (s, 2C) 58.84 (s, 1C) 101.12 (s, 1C) 108.86 (s, 1C) 113.28 (s, 1C) 115.11 (s, 1C) 116.78 (s, 1C) 124.64 (s, 1C) 125.43 (s, 1C) 126.41 (s, 1C) 127.70 (s, 1C) 128.59 (s, 2C) 129.03 (s, 1C) 129.34 (s, 2C) 129.68 (s, 1C) 130.15 (s, 1C) 130.92 (s, 1C) 131.89 (s, 1C) 132.38 (s, 1C) 135.48 (s, 1C) 138.39 (s, 1C) 139.17 (s, 1C) 140.47 (s, 1C) 148.76 (s, 1C) 153.95 (s, 1C) 158.92 (s, 1C) 162.84 (s, 1C) 165.97 (s, 1C); **HRMS (ESI):** [R$_t$]: 5.88 min, *m/z* for $C_{34}H_{32}N_5O_4$ ([M+H$^+$]), 574.2448 cacld., 574.2441 found.

Synthesis of methyl 6-(4-((4-(6-acrylamido-2-oxo-2,3-dihydro-1H-benzo[d]imidazol-1-yl)piperidin-1-yl)methyl)phenyl)-5-phenylnicotinate (**5**).
Chloropyridine (**S10**, 40.0 mg, 0.12 mmol, 1.0 eq.), (4-((4-(6-acrylamido-2-oxo-2,3-dihydro-1*H*-benzo[*d*]imidazol-1-yl)piperidin-1-yl)methyl)phenyl)boronic acid (50.4 mg, 0.12 mmol, 1.0 eq.), K_2CO_3 (33.2 mg, 0.24 mmol, 2.0 eq.) and tetrakis(triphenylphosphine)palladium(0) (9.2 mg, 0.008 mol, 0.1 eq.) were used following common procedure E and yielded the final compound as a solid (47.6 mg, 0.08 mmol, 50 %).**^{1}H-NMR** (700 MHz, DMSO-*d_6*) *δ* ppm 1.95 (d, *J* = 12.48 Hz, 2 H) 2.62 (d, *J* = 13.01 Hz, 2 H) 3.20 (d, *J* = 12.37 Hz, 2 H) 3.46 (d, *J* = 11.51 Hz, 2 H) 3.94 (s, 3 H) 4.33 (d, *J* = 4.95 Hz, 2 H) 4.35 - 4.41 (m, 1 H) 5.74 (d, *J* = 11.94 Hz, 1 H) 6.24 (d, *J* = 16.89 Hz, 1 H) 6.39 - 6.47 (m, 1 H) 6.94 (s, 2 H) 7.26 (d, *J* = 5.92 Hz, 2 H) 7.33 - 7.39 (m, 3 H) 7.43 - 7.48 (m, 3 H) 7.84 (s, 1 H) 8.25 (s, 1 H) 9.18 (s, 1 H) 9.71 - 9.78 (m, 1 H) 10.08 (s, 1 H) 10.88 (s, 1 H); **^{13}C-NMR** (176 MHz, DMSO-*d_6*) *δ* ppm 25.90 (s, 2 C) 47.54 (s, 1 C) 51.10 (s, 1 C) 52.58 (s, 2 C) 58.81 (s, 1 C) 101.39 (s, 1 C) 108.86 (s, 1 C) 113.28 (s, 1 C) 115.23 (s, 1 C) 116.90 (s, 1 C) 124.42 (s, 1 C) 124.63 (s, 1 C) 126.42 (s, 1 C) 127.93 (s, 1 C) 128.62 (s, 2 C) 129.03 (s, 1 C) 129.33 (s, 2 C) 129.79 (s, 1 C) 130.15 (s, 1 C) 130.94 (s, 1 C) 131.88 (s, 1 C) 132.38 (s, 1 C) 135.57 (s, 1 C) 138.21 (s, 1 C) 139.03 (s, 1 C) 140.31 (s, 1 C) 148.49 (s, 1 C) 153.94 (s, 1 C) 159.26 (s, 1 C) 162.83 (s, 1 C) 164.96 (s, 1 C); **HRMS (ESI):** [R$_t$]: 4.81 min, *m/z* for $C_{35}H_{34}N_5O_4$ ([M+H$^+$]), 588.2605 cacld., 588.2600 found.

Synthesis of 6-(4-((4-(6-acrylamido-2-oxo-2,3-dihydro-1H-benzo[d]imidazol-1-yl)piperidin-1-yl)methyl)phenyl)-N-methyl-5-phenylnicotinamide (**6**).
Chloropyridine (**S24**, 20.0 mg, 0.08 mmol, 1.0 eq.), (4-((4-(6-acrylamido-2-oxo-2,3-dihydro-1*H*-benzo[*d*]imidazol-1-yl)piperidin-1-yl)methyl)phenyl)boronic acid (33.6 mg, 0.08 mmol, 1.0 eq.), K_2CO_3 (22.4 mg, 0.16 mmol, 2.0 eq.) and tetrakis(triphenylphosphine)palladium(0) (9.2 mg, 0.008 mol, 0.1 eq.) were used following common procedure E and yielded the final compound as a solid (23.3 mg, 0.04 mmol, 49 %).**^{1}H-NMR** (400 MHz, DMSO-*d_6*) *δ* ppm 1.96 (d, *J* = 12.72 Hz, 2 H) 2.55 - 2.69 (m, 2 H) 2.84 (d, *J* = 4.65 Hz, 3 H) 3.13 - 3.26 (m, 2 H) 3.46 (d, *J* = 11.25 Hz, 2 H) 4.32 (d, *J* = 4.16 Hz, 2 H) 4.37 - 4.41 (m, 1 H) 5.71 - 5.77 (m, 1 H) 6.24 (dd, *J* = 16.99, 2.08 Hz, 1 H) 6.43 (dd, *J* = 16.87, 10.03 Hz, 1 H) 6.94 (s, 1 H) 7.22 - 7.28 (m, 2 H) 7.32 - 7.38 (m, 3 H) 7.44 (s, 4 H) 7.84 (s, 1 H) 8.24 (d, *J* = 1.96 Hz, 1 H) 8.77 (d, *J* = 4.65 Hz, 1 H) 9.08 (d, *J* = 2.20 Hz, 1 H) 9.70 - 9.78 (m, 1 H) 10.09 (s, 1 H) 10.88 (s, 1 H); **^{13}C-NMR** (176 MHz, DMSO-*d_6*) *δ* ppm 25.96 (s, 1C) 26.30 (s, 2C) 47.61 (s, 1C) 51.16 (s, 2C) 58.96 (s,

1C) 101.53 (s, 1C) 108.97 (s, 1C) 113.43 (s, 1C) 115.02 (s, 1C) 116.68 (s, 1C) 124.73 (s, 1C) 126.59 (s, 1C) 127.82 (s, 1C) 128.61 (s, 1C) 128.67 (s, 1C) 129.10 (s, 1C) 129.50 (s, 2C) 130.20 (s, 1C) 130.99 (s, 1C) 131.91 (s, 1C) 132.43 (s, 1C) 135.21 (s, 1C) 137.27 (s, 1C) 138.74 (s, 1C) 140.71 (s, 1C) 147.15 (s, 1C) 154.04 (s, 1C) 157.41 (s, 1C) 158.17 (s, 1C) 158.39 (s, 1C) 162.97 (s, 1C) 164.80 (s, 1C); **HRMS (ESI):** [R_t]: 5.60 min, *m/z* for $C_{35}H_{35}N_6O_3$ ([M+H$^+$]), 587.2765 cacld., 587.2757 found.

Synthesis of N-(2-oxo-3-(1-(4-(3-phenyl-5-(pyrrolidin-1-ylmethyl)pyridin-2-yl)benzyl)piperidin-4-yl)-2,3-dihydro-1H-benzo[d]imidazol-5-yl)acrylamide (**7**).

Chloropyridine (**S13**, 20.0 mg, 0.07 mmol, 1.0 eq.), (4-((4-(6-acrylamido-2-oxo-2,3-dihydro-1*H*-benzo[*d*]imidazol-1-yl)piperidin-1-yl)methyl)phenyl)boronic acid (29.4 mg, 0.07 mmol, 1.0 eq.), K_2CO_3 (19.4 mg, 0.14 mmol, 2.0 eq.) and tetrakis(triphenylphosphine)palladium(0) (8.0 mg, 0.007 mol, 0.1 eq.) were used following common procedure E and yielded the final compound as a solid (7.7 mg, 0.01 mmol, 18 %).**^{1}H-NMR** (600 MHz, DMSO-d_6) δ ppm 1.64 (d, J = 9.92 Hz, 4 H) 1.72 (s, 4 H) 3.51 (s, 4 H) 3.66 - 3.75 (m, 2 H) 4.10 (m, 1 H) 5.99 (dd, J = 1.00 Hz, 1 H) 6.41 (dd, J = 11.14, 10.07 Hz, 1 H) 7.19 (m, 4 H) 7.24 (m, 5 H) 7.30 (t, J = 6.33 Hz, 5 H) 7.69 (d, J = 4.58 Hz, 4 H) 8.58 (s, 1 H); **^{13}C-NMR** (151 MHz, DMSO-d_6) δ ppm 23.15 (s, 2C) 28.68 (s, 1) 50.12 (s, 1C) 52.64 (s, 1C) 53.55 (s, 2C) 56.24 (s, 1C) 61.55 (s, 1C) 96.70 (s, 1C) 100.90 (s, 1C) 108.66 (s, 1C) 112.10 (s, 1C) 124.49 (s, 1C) 127.29 (s, 1C) 128.25 (s, 1C) 128.40 (s, 2C) 129.34 (s, 2C) 129.56 (s, 2C) 134.91 (s, 1C) 138.63 (s, 1C) 139.65 (s, 1C) 148.30 (s, 1C) 154.00 (s, 1C) 162.77 (s, 1C); **HPLC-MS (ESI):** *m/z* for $C_{38}H_{40}N_6O_2$ ([M+H$^+$]), 613.3285 cacld., 613.3288 found.

Synthesis of N-(2-oxo-3-(1-(4-(3-phenyl-5-(pyrrolidin-1-carbonyl)pyridin-2-yl)benzyl)piperidin-4-yl)-2,3-dihydro-1H-benzo[d]imidazol-5-yl)acrylamide (**8**).

Chloropyridine (**S27**, 20.0 mg, 0.07 mmol, 1.0 eq.), (4-((4-(6-acrylamido-2-oxo-2,3-dihydro-1*H*-benzo[*d*]imidazol-1-yl)piperidin-1-yl)methyl)phenyl)boronic acid (29.4 mg, 0.07 mmol, 1.0 eq.), K_2CO_3 (19.4 mg, 0.14 mmol, 2.0 eq.) and tetrakis(triphenylphosphine)palladium(0) (8.0 mg, 0.007 mol, 0.1 eq.) were used following common procedure E and yielded the final compound as a solid (15.1 mg, 0.02 mmol, 34 %). **^{1}H-NMR** (600 MHz, MeOD-d_4) δ ppm 1.95 - 2.06 (m, 4H) 2.08 - 2.15 (m, 2H) 2.73 - 2.84 (m, 2H) 3.20 - 3.29 (m, 2H) 3.59 - 3.70 (m, 4H) 4.36 - 4.41 (m, 2H) 4.47 - 4.57 (m, 1H) 5.75 - 5.82 (m, 1H) 6.35 - 6.49 (m, 1H) 6.94 - 6.98 (m, 1H) 7.02 - 7.06 (m, 1H) 7.20 - 7.26 (m, 2H) 7.28 - 7.34 (m, 4H) 7.44 - 7.53 (m, 6H) 7.94 - 7.98 (m, 1H) 8.02 (s, 1H) 8.77 (s, 1H) 10.06 (s, 1H) 10.76 (s, 1H); **^{13}C-NMR** (151 MHz, MeOD-d_4) δ ppm 25.39 (s, 2C) 27.42 (s, 2C) 47.87 (s, 2C) 51.09 (s, 1C) 53.42 (s, 2C) 61.42 (s, 1C) 101.47 (s, 1C) 103.62 (s, 1C) 110.89 (s, 1C) 115.61 (s, 1C) 126.70 (s, 1C) 127.96 (s, 1C) 129.19 (s, 1C) 129.91 (s, 2C) 130.95 (s, 2C) 132.13 (s, 1 C) 132.30 (s, 1C) 132.53 (s, 1C) 133.38 (s, 1C) 134.11 (s, 1C) 138.09 (s, 1C) 139.05 (s, 1C) 139.14 (s, 1C) 140.03 (s, 1C) 142.82 (s, 1C) 147.70 (s, 1C) 156.59 (s, 1C) 158.67 (s, 1C) 166.21 (s, 1C) 168.63 (s, 1C); **HRMS (ESI):** [R_t]: 6.21 min, *m/z* for $C_{38}H_{39}N_6O_3$ ([M+H$^+$]), 626.3078 cacld., 627.3068 found.

Synthesis of N-(3-(1-(4-(5-((isobutylamino)methyl)-3-phenylpyridin-2-yl)benzyl)piperidin-4-yl)-2-oxo-2,3-dihydro-1H-benzo[d]imidazol-5-yl)acrylamide (**9**).

Chloropyridine (**S25**, 20.0 mg, 0.07 mmol, 1.0 eq.), (4-((4-(6-acrylamido-2-oxo-2,3-dihydro-1*H*-benzo[*d*]imidazol-1-yl)piperidin-1-yl)methyl)phenyl)boronic acid (29.4 mg, 0.07 mmol, 1.0 eq.), K_2CO_3 (19.4 mg, 0.14 mmol, 2.0 eq.) and tetrakis(triphenylphosphine)palladium(0) (8.0 mg, 0.007 mol, 0.1 eq.) were used following common procedure E and yielded the final compound as a solid (34.4 mg, 0.06 mmol, 80 %). **^{1}H-NMR** (700 MHz, MeOD-d_4) δ ppm 1.07 (s, 6 H) 2.10 (m, 2 H) 3.02 (dd, J=7.10, 3.01 Hz, 2 H) 4.54 (m, 2 H) 6.14 (dd, J=17.97, 18.93 Hz, 4 H) 6.46 (dd, J=16.99, 10.11 Hz, 1 H) 7.16 (dd, J=139.18, 135.31 Hz, 4 H) 7.31 (dd, J=19.79, 8.60 Hz, 4 H) 7.49 (s, 5 H) 7.55 (s, 2 H) 7.57 (dd, J=7.96, 3.01 Hz, 2 H) 7.64 (s, 2 H) 7.66 (m, 4 H) 8.10 (s, 1 H) 8.79 (s, 1 H); **^{13}C-NMR** (151 MHz, MeOD-d_4) δ ppm 8.96 (s, 2C) 20.10 (s, 2C) 27.13 (s, 1C) 47.69 (s, 2C) 49.54 (s, 1C) 52.54 (s, 1C) 56.06 (s, 1C) 110.46 (s, 1C) 126.33 (s, 1C) 127.46 (s, 1C) 127.93 (s, 1C) 128.89 (s, 1C) 129.49 (s, 1C) 129.73 (s, 2C) 129.80 (s, 2C) 130.47 (s, 1C) 131.69 (s, 1C) 131.95 (s, 1C) 132.11 (s, 1C) 132.20 (s, 1C) 132.71 (s, 1C) 132.84 (s, 2C) 132.90 (s, 2C) 133.57 (s, 1C) 133.58 (s, 1C) 133.67 (s, 1C); **HRMS (ESI):** *m/z* for $C_{38}H_{42}N_6O_2$ ([M+H$^+$]), 615.3442 cacld., 615.3441 found.

Synthesis of 6-(4-((4-(6-acrylamido-2-oxo-2,3-dihydro-1H-benzo[d]imidazol-1-yl)piperidin-1-yl)methyl)phenyl)-N-isobutyl-5-phenylnicotinamide (**10**).

Chloropyridine (**S25**, 20.0 mg, 0.07 mmol, 1.0 eq.), (4-((4-(6-acrylamido-2-oxo-2,3-dihydro-1*H*-benzo[*d*]imidazol-1-yl)piperidin-1-yl)methyl)phenyl)boronic acid (29.4 mg, 0.07 mmol, 1.0 eq.), K_2CO_3 (19.4 mg, 0.14 mmol, 2.0 eq.) and tetrakis(triphenylphosphine)palladium(0) (8.0 mg, 0.007 mol, 0.1 eq.) were used following common procedure E and yielded the final compound as a solid (23.0 mg, 0.03 mmol, 52 %). **^{1}H-NMR** (400 MHz, DMSO-d_6) δ ppm 0.91 (d, J = 6.85 Hz, 6 H) 1.87 (dt, J = 13.45, 6.72 Hz, 1 H) 1.96 (d, J = 11.25 Hz, 2 H) 2.55 - 2.68 (m, 2 H) 3.11 - 3.17 (m, 2 H) 3.17 - 3.27 (m, 2 H) 3.46 (d, J = 11.00 Hz, 2 H) 4.32 (d, J = 4.16 Hz, 2 H) 4.37 (s, 1 H) 5.74 (d, J = 12.23 Hz, 1 H) 6.24 (dd, J = 16.99, 2.08 Hz, 1 H) 6.43 (dd, J = 16.87, 10.03 Hz, 1 H) 6.94 (s, 2 H) 7.22 - 7.29 (m, 2 H) 7.32 - 7.39 (m, 3 H) 7.44 (s, 3 H) 7.84 (s, 1 H) 8.27 (d, J = 2.20 Hz, 1 H) 8.78 (t, J = 5.75 Hz, 1 H) 9.09 (d, J = 1.96 Hz, 1 H) 9.74 (s, 1 H) 10.09 (s, 1 H) 10.88 (s, 1 H); **^{13}C-NMR** (176 MHz, MeOD-d_4) δ ppm 20.28 (s, 2C) 25.98 (s, 2C) 28.18 (s, 1C) 46.85 (s, 2C) 47.63 (s, 1C) 51.17 (s, 1C) 59.01 (s, 1C) 108.99 (s, 1C) 113.43 (s, 1C) 115.15 (s, 1C) 116.85 (s, 1C) 124.70 (s, 1C) 126.61 (s, 1C) 127.82 (s, 1C) 128.60 (s, 1C) 128.83 (s, 2C) 129.10 (s, 1C) 129.49 (s, 2C) 130.18 (s, 1C) 130.99 (s, 1C) 131.92 (s, 1C) 132.43 (s, 1C) 135.18 (s, 1C) 137.27 (s, 1C) 138.76 (s, 1C) 140.75 (s, 1C) 147.33 (s, 1C) 154.06 (s, 1C) 157.40 (s, 1C) 162.99 (s, 1C) 164.44 (s, 1C);**HRMS (ESI):** [R_t]: 6.34 min, *m/z* for $C_{38}H_{41}N_6O_3$ ([M+H$^+$]), 629.3234 cacld., 629.3223 found.

Synthesis of N-(3-(1-(4-(5-((1H-imidazol-1-yl)methyl)-3-phenylpyridin-2-yl)benzyl)piperidin-4-yl)-2-oxo-2,3-dihydro-1H-benzo[d]imidazol-5-yl)acrylamide (**11**).

Chloropyridine (**S14**, 20.0 mg, 0.07 mmol, 1.0 eq.), (4-((4-(6-acrylamido-2-oxo-2,3-dihydro-1*H*-benzo[*d*]imidazol-1-yl)piperidin-1-yl)methyl)phenyl)boronic acid (29.4 mg, 0.07 mmol, 1.0 eq.), K_2CO_3 (19.4 mg, 0.14 mmol, 2.0 eq.) and

tetrakis(triphenylphosphine)palladium(0) (8.0 mg, 0.007 mol, 0.1 eq.) were used following common procedure E and yielded the final compound as a solid (8.9 mg, 0.02 mmol, 21 %). **^{1}H-NMR** (500 MHz, MeOD-*d*$_4$) *δ* ppm 2.08 (dd, J=30.21, 13.58 Hz, 2 H) 4.38 (s, 2 H) 4.48 - 4.56 (m, 1 H) 5.65 (d, J=4.73 Hz, 2 H) 6.08 (dd, J=297.85, 292.51 Hz, 4 H) 6.45 (dd, J=26.55, 9.00 Hz, 1 H) 7.14 (d, J=8.24 Hz, 4 H) 7.17 - 7.22 (m, 1 H) 7.24 - 7.32 (m, 5 H) 7.32 - 7.44 (m, 2 H) 7.48 (s, 2 H) 7.64 (s, 4 H) 7.79 (s, 1 H) 7.99 (s, 1 H) 8.76 (s, 1 H) 9.18 (s, 1 H); **^{13}C-NMR** (126 MHz, MeOD-*d*$_4$) *δ* ppm 49.49 (s, 2C) 49.61 (s, 2C) 50.74 (s, 2C) 53.16 (s, 1C) 121.82 (s, 2C) 123.36 (s, 2C) 124.35 (s, 1C) 124.66 (s, 1C) 127.72 (s, 1C) 128.83 (s, 1C) 129.09 (s, 2C) 129.67 (s, 2C) 130.72 (s, 2C) 130.88 (s, 1C) 131.16 (s, 1C) 131.91 (s, 1C) 132.16 (s, 2C) 132.37 (s, 2C) 136.95 (s, 2C) 138.68 (s, 1C) 139.77 (s, 1C) 140.73 (s, 2C) 142.44 (s, 1C) 148.94 (s, 2C) 157.97 (s, 1C) 164.01 (s, 1C) 179.72 (s, 1C); **HRMS (ESI):** *m/z* for $C_{37}H_{35}N_7O_2$ ([M+H$^+$]), 610.2925 cacld., 610.2923 found.

Synthesis of N-(3-(1-(4-(5-((1-methyl-1H-pyrazol-4-yl)methyl)-3-phenylpyridin-2-yl)benzyl)piperidin-4-yl)-2-oxo-2,3-dihydro-1H-benzo[d]imidazol-5-yl)acrylamide (**12**).
Chloropyridine (**S15**, 20.0 mg, 0.06 mmol, 1.0 eq.), (4-((4-(6-acrylamido-2-oxo-2,3-dihydro-1*H*-benzo[*d*]imidazol-1-yl)piperidin-1-yl)methyl)phenyl)boronic acid (28.2 mg, 0.06 mmol, 1.0 eq.), K_2CO_3 (15.5 mg, 0.12 mmol, 2.0 eq.) and tetrakis(triphenylphosphine)palladium(0) (6.9 mg, 0.006 mol, 0.1 eq.) were used following common procedure E and yielded the final compound as a solid (12.0 mg, 0.02 mmol, 30 %). **^{1}H-NMR** (600 MHz, DMSO-*d*$_6$) *δ* ppm 1.64 (d, *J* = 9.90 Hz, 2H) 2.07 (s, 2H) 2.21 - 2.34 (m, 2H) 2.93 (d, *J* = 10.64 Hz, 2H) 3.77 (s, 3H) 3.87 (s, 2H) 4.07 - 4.15 (m, 1H) 5.73 (dd, *J* = 10.27, 1.83 Hz, 1H) 6.25 (d, *J* = 1.83 Hz, 1H) 6.39 (d, *J* = 10.27 Hz, 1H) 6.91 (d, *J* = 8.44 Hz, 1H) 7.16 (dd, *J* = 7.52, 1.65 Hz, 2H) 7.19 - 7.25 (m, 4H) 7.26 - 7.32 (m, 4H) 7.35 (s, 2H) 7.57 (s, 1H) 7.61 (d, *J* = 1.83 Hz, 1H) 7.68 (s, 1H) 8.55 (d, *J* = 1.83 Hz, 1H) 10.07 (s, 1H) 10.80 (s, 1H); **^{13}C-NMR** (151 MHz, DMSO-*d*$_6$) *δ* ppm 25.71 (s, 2C) 26.97 (s, 1C) 29.15 (s, 1C) 38.87 (s, 1C) 50.60 (s, 1 C) 53.09 (s, 2C) 56.50 (s, 1C) 62.01 (s, 1C) 62.49 (s, 1C) 101.38 (s, 1C) 109.11 (s, 1C) 112.58 (s, 1C) 119.52 (s, 1C) 124.96 (s, 1C) 126.81 (s, 1C) 127.70 (s, 1C) 128.68 (s, 1C) 128.81 (s, 2C) 129.45 (s, 1C) 129.79 (s, 3C) 129.97 (s, 1C) 132.51 (s, 1C) 133.12 (s, 1C) 135.49 (s, 1C) 136.31 (s, 1C) 138.04 (s, 1C) 138.66 (s, 1C) 139.11 (s, 1C) 140.13 (s, 1C) 148.58 (s, 1C) 154.32 (s, 1C) 154.47 (s, 1C) 163.24 (s, 1C); **HRMS (ESI):** [R$_t$]: 4.88 min, *m/z* for $C_{38}H_{37}N_7O_2$ ([M+H$^+$]), 624.3081 cacld., 624.3071 found.

Synthesis of 6-(4-((4-(6-acrylamido-2-oxo-2,3-dihydro-1H-benzo[d]imidazol-1-yl)piperidin-1-yl)methyl)phenyl)-N-(1-methyl-1H-pyrazol-4-yl)-5-phenylnicotinamide (**13**).
Chloropyridine (**S26**, 10.0 mg, 0.03 mmol, 1.0 eq.), (4-((4-(6-acrylamido-2-oxo-2,3-dihydro-1*H*-benzo[*d*]imidazol-1-yl)piperidin-1-yl)methyl)phenyl)boronic acid (12.6 mg, 0.03 mmol, 1.0 eq.), K_2CO_3 (8.3 mg, 0.06 mmol, 2.0 eq.) and tetrakis(triphenylphosphine)palladium(0) (3.4 mg, 0.003 mol, 0.1 eq.) were used following common procedure E and yielded the final compound as a solid (10.7 mg, 0.02 mmol, 54 %).**^{1}H-NMR** (500 MHz, DMSO-*d*$_6$) *δ* ppm 1.91 - 2.02 (m, 2 H) 2.57 - 2.67 (m, 2 H) 3.15 - 3.26 (m, 2 H) 3.43 - 3.51 (m, 2 H) 3.84 (s, 3 H) 4.29 - 4.35 (m, 2 H) 4.37 - 4.42 (m, 1 H) 5.72 - 5.77 (m, 1 H) 6.21 - 6.27 (m, 1 H) 6.38 - 6.47 (m, 1 H) 6.92 - 6.95 (m, 2 H) 7.28 - 7.31 (m, 2 H) 7.36 (s, 3 H) 7.46 (s, 3 H) 7.59 (s, 1 H) 7.84 - 7.86 (m, 1 H) 8.07 (s, 1 H) 8.37 (d, *J* = 2.14 Hz, 1 H) 9.18 (d, *J* = 2.14 Hz, 1 H) 10.10 (s, 1 H) 10.72 (s, 1 H) 10.90 (s, 1 H); **^{13}C-NMR** (151 MHz, DMSO-*d*$_6$) *δ* ppm 25.48 (s, 1C) 28.67 (s, 2C) 38.73 (s, 1C) 50.11 (s, 1C) 52.63 (s, 2C) 61.48 (s, 1C) 100.89 (s, 1C) 108.62 (s, 1C) 112.10 (s, 1C) 121.13 (s, 1C) 121,82 (s, 1C) 124.47 (s, 1C) 126.33 (s, 1C) 127.34 (s, 1C) 127.66 (s, 1C) 128.23 (s, 1C) 128.49 (s, 2C) 128.96 (s, 1C) 129.38 (s, 2C) 130.14 (s, 2C) 131.75 (s, 1C) 132.04 (s, 1C) 132.63 (s, 1C) 134.89 (s, 1C) 137.23 (s, 1C) 137.93 (s, 1C) 138.54 (s, 1C) 139.04 (s, 1C) 147.35 (s, 1C) 153.98 (s, 1C) 158.27 (s, 1C) 161.47 (s, 1C) 162.75 (s, 1C); **HPLC-MS (ESI):** [R$_t$]: 5.13 min, *m/z* for $C_{38}H_{37}N_8O_3$ ([M+H$^+$]), 653.2983 cacld., 653.2976 found.

Synthesis of N-(3-(1-(4-(5-((1H-pyrazol-4-yl)methyl)-3-phenylpyridin-2-yl)benzyl)piperidin-4-yl)-2-oxo-2,3-dihydro-1H-benzo[d]imidazol-5-yl)acrylamide (**14**).
Chloropyridine (**S22**, 20.0 mg, 0.06 mmol, 1.0 eq.), (4-((4-(6-acrylamido-2-oxo-2,3-dihydro-1*H*-benzo[*d*]imidazol-1-yl)piperidin-1-yl)methyl)phenyl)boronic acid (28.2 mg, 0.06 mmol, 1.0 eq.), K_2CO_3 (15.5 mg, 0.12 mmol, 2.0 eq.) and tetrakis(triphenylphosphine)palladium(0) (6.9 mg, 0.006 mol, 0.1 eq.) were used following common procedure E and yielded the final compound as a solid (12.8 mg, 0.02 mmol, 33 %). **^{1}H-NMR** (600 MHz, DMSO-*d*$_6$) *δ* ppm 1.64 (d, *J* = 9.90 Hz, 2H) 1.98 - 2.14 (m, 2H) 2.29 (d, *J* = 10.27 Hz, 2H) 2.94 (d, *J* = 9.54 Hz, 2H) 3.51 (s, 2H) 4.07 - 4.15 (m, 1H) 5.48 (s, 2H) 5.70 - 5.77 (m, 1H) 6.24 (dd, *J* = 17.06, 2.02 Hz, 1H) 6.27 - 6.31 (m, 1H) 6.41 (dd, *J* = 16.87, 9.90 Hz, 1H) 6.91 (d, *J* = 8.44 Hz, 1H) 7.09 - 7.13 (m, 1H) 7.20 - 7.25 (m, 4H) 7.27 - 7.33 (m, 4H) 7.50 (d, *J* = 1.10 Hz, 1H) 7.60 - 7.72 (m, 2H) 7.95 (d, *J* = 2.20 Hz, 1H) 8.56 (d, *J* = 2.20 Hz, 1H) 10.07 (s, 1H) 10.80 (s, 1H); **^{13}C-NMR** (126 MHz, DMSO-*d*$_6$) *δ* ppm 28.62 (s, 2C) 50.08 (s, 1C) 51.70 (s, 2C) 52.59 (s, 1C) 54.73 (s, 1C) 61.33 (s, 1C) 100.91 (s, 1C) 105.66 (s, 1C) 108.63 (s, 1C) 112.13 (s, 1C) 124.48 (s, 1C) 126.34 (s, 1C) 127.40 (s, 1C) 128.29 (s, 1C) 128.41 (s, 2C) 128.97 (s, 1C) 129.21 (s, 2C) 129.52 (s, 1C) 130.43 (s, 2C) 131.88 (s, 1C) 132.04 (s, 1C) 132.63 (s, 1C) 135.09 (s, 1C) 137.84 (s, 1C) 139.30 (s, 1C) 139.41 (s, 2C) 147.48 (s, 1C) 153.99 (s, 1C) 155.49 (s, 1C) 162.76 (s, 1C); **HRMS (ESI):** [R$_t$]: 6.19 min, *m/z* for $C_{37}H_{36}N_7O_2$ ([M+H$^+$]), 610.2925 cacld., 610.2925 found.

Synthesis of N-(3-(1-(4-(5-((5-methylfuran-2-yl)methyl)-3-phenylpyridin-2-yl)benzyl)piperidin-4-yl)-2-oxo-2,3-dihydro-1H-benzo[d]imidazol-5-yl)acrylamide (**15**).
Chloropyridine (**S17**, 20.0 mg, 0.07 mmol, 1.0 eq.), (4-((4-(6-acrylamido-2-oxo-2,3-dihydro-1*H*-benzo[*d*]imidazol-1-yl)piperidin-1-yl)methyl)phenyl)boronic acid (29.4 mg, 0.07 mmol, 1.0 eq.), K_2CO_3 (19.4 mg, 0.14 mmol, 2.0 eq.) and tetrakis(triphenylphosphine)palladium(0) (8.0 mg, 0.007 mol, 0.1 eq.) were used following common procedure E and yielded the final compound as a solid (17.4 mg, 0.03 mmol, 39 %). **^{1}H-NMR** (500 MHz, MeOD-*d*$_4$) *δ* ppm 1.60 - 1.69 (m, 2H) 2.03 - 2.14 (m, 2H) 2.21 (s, 3H) 2.25 - 2.35 (m, 2H) 2.90 - 2.99 (m, 2H) 3.55 (m, 2H) 4.04 (s, 1H) 4.08 - 4.14 (m, 1H) 5.69 - 5.76 (m, 1H) 6.20 - 6.27 (m, 1H) 6.34 - 6.44 (m, 1H) 6.88 - 6.94 (m, 1H) 7.14 - 7.23 (m, 4H) 7.23 - 7.32 (m, 5H) 7.35 (s, 2H) 7.62 - 7.74 (m, 2H) 8.52 - 8.56 (m, 1H) 10.02 (s, 1H) 10.73 (s, 1H); **^{13}C-NMR** (176 MHz, DMSO-*d*$_6$) *δ* ppm 13.26 (s, 1C) 25.47 (s, 1C) 28.65 (s, 1C) 30.29 (s, 1C) 50.12

(s, 2C) 52.62 (s, 1C) 56.00 (s, 1C) 61.51 (s, 1C) 100.88 (s, 1C) 106.46 (s, 1C) 107.40 (s, 1C) 108.61 (s, 1C) 112.10 (s, 1C) 115.27 (s, 1C) 124.47 (s, 1C) 126.31 (s, 1C) 126.44 (s, 1C) 126.93 (s, 1C) 127.29 (s, 1C) 128.20 (s, 1C) 128.36 (s, 1C) 128.88 (s, 1C) 128.96 (s, 1C) 129.26 (s, 1C) 129.49 (s, 1C) 132.03 (s, 1C) 132.62 (s, 1C) 132.67 (s, 1C) 135.05 (s, 1C) 137.70 (s, 1C) 138.46 (s, 1C) 139.50 (s, 1C) 148.19 (s, 1C) 150.66 (s, 1C) 151.38 (s, 1C) 153.98 (s, 1C) 154.34 (s, 1C) 162.75 (s, 1C); **HPLC-MS (ESI):** [R_t]: 5.09 min, *m/z* for $C_{39}H_{37}N_5O_3$ ([M+H$^+$]), 624.2969 calcd., 624.2969 found.

Synthesis of N-(3-(1-(4-(5-(3-hydroxybenzyl)-3-phenylpyridin-2-yl)benzyl)piperidin-4-yl)-2-oxo-2,3-dihydro-1H-benzo[d]imidazol-5-yl)acrylamide (**16**).
Chloropyridine (**S18**, 20.0 mg, 0.07 mmol, 1.0 eq.), (4-((4-(6-acrylamido-2-oxo-2,3-dihydro-1*H*-benzo[*d*]imidazol-1-yl)piperidin-1-yl)methyl)phenyl)boronic acid (29.4 mg, 0.07 mmol, 1.0 eq.), K_2CO_3 (18.6 mg, 0.14 mmol, 2.0 eq.) and tetrakis(triphenylphosphine)palladium(0) (8.0 mg, 0.007 mol, 0.1 eq.) were used following common procedure E and yielded the final compound as a solid (21.2 mg, 0.03 mmol, 47 %). **^{1}H-NMR** (700 MHz, DMSO-d_6) δ ppm 1.95 (d, J = 12.26 Hz, 2 H) 2.61 (d, J = 14.09 Hz, 2 H) 3.15 - 3.24 (m, 2 H) 3.45 (d, J = 11.51 Hz, 2 H) 4.07 (s, 2 H) 4.27 - 4.32 (m, 2 H) 4.35 - 4.39 (m, 1 H) 4.51 (s, 2 H) 5.74 (d, J = 10.22 Hz, 1 H) 6.23 (d, J = 17.21 Hz, 1 H) 6.43 (dd, J = 16.83, 10.16 Hz, 1 H) 6.95 (s, 2 H) 7.10 - 7.17 (m, 4 H) 7.30 (s, 2 H) 7.35 - 7.41 (m, 5 H) 7.71 (s, 1 H) 7.83 (s, 1 H) 8.61 (s, 1 H) 9.72 (s, 1 H) 10.08 (s, 1 H) 10.88 (s, 1 H); **^{13}C-NMR** (126 MHz, DMSO-d_6) δ ppm 25.89 (s, 2 C) 36.88 (s, 1C) 47.55 (s, 1C) 51.06 (s, 2 C) 58.90 (s, 1C) 101.39 (s, 1C) 108.86 (s, 1C) 113.27 (s, 1C) 115.34 (s, 1C) 117.02 (s, 1C) 124.63 (s, 1C) 126.41 (s, 1C) 127.47 (s, 1C) 128.42 (s, 2 C) 128.88 (s, 1C) 129.01 (s, 1C) 129.33 (s, 2 C) 129.93 (s, 3 C) 130.04 (s, 2 C) 130.79 (s, 2 C) 131.89 (s, 1C) 132.38 (s, 1C) 135.42 (s, 1C) 136.03 (s, 1C) 138.74 (s, 1C) 139.11 (s, 1C) 140.99 (s, 1C) 148.25 (s, 2 C) 153.23 (s, 1C) 153.94 (s, 1C) 162.83 (s, 1C); **HPLC-MS (ESI):** [R_t]: 6.07 min, *m/z* for $C_{40}H_{37}N_5O_3$ ([M+H$^+$]), 636.2969 calcd., 636.2968 found.

Synthesis of N-(3-(1-(4-(5-(3-aminobenzyl)-3-phenylpyridin-2-yl)benzyl)piperidin-4-yl)-2-oxo-2,3-dihydro-1H-benzo[d]imidazol-5-yl)acrylamide (**17**).
Chloropyridine (**S20**, 20.0 mg, 0.07 mmol, 1.0 eq.), (4-((4-(6-acrylamido-2-oxo-2,3-dihydro-1*H*-benzo[*d*]imidazol-1-yl)piperidin-1-yl)methyl)phenyl)boronic acid (29.4 mg, 0.07 mmol, 1.0 eq.), K_2CO_3 (18.7 mg, 0.14 mmol, 2.0 eq.) and tetrakis(triphenylphosphine)palladium(0) (8.0 mg, 0.007 mol, 0.1 eq.) were used following common procedure E and yielded the final compound as a solid (9.4 mg, 0.01 mmol, 21 %). **^{1}H-NMR** (600 MHz, MeOD-d_4) δ ppm 2.06 - 2.14 (m, 2H) 2.71 - 2.84 (m, 2H) 3.21 - 3.29 (m, 2H) 3.56 - 3.71 (m, 2H) 4.28 (s, 2H) 4.35 - 4.42 (m, 2H) 4.47 - 4.57 (m, 1H) 5.74 - 5.84 (m, 1H) 6.35 - 6.41 (m, 1H) 6.42 - 6.48 (m, 1H) 6.94 - 7.06 (m, 2H) 7.14 - 7.20 (m, 1H) 7.30 (s, 4H) 7.33 - 7.39 (m, 1H) 7.47 (s, 2H) 7.52 (d, J = 7.70 Hz, 4H) 7.91 - 7.94 (m, 1H) 7.97 - 8.00 (m, 1H) 8.61 - 8.66 (m, 1H) 8.98 - 9.01 (m, 1H) 10.05 - 10.07 (m, 1H) 10.76 - 10.79 (m, 1H); **^{13}C-NMR** (151 M Hz, MeOD-d_4) δ ppm 27.55 (s, 2C) 38.78 (s, 1C) 53.37 (s, 2C) 61.35 (s, 1C) 103.53 (s, 1C) 110.85 (s, 1C) 115.65 (s, 1C) 117.20 (s, 1C) 122.36 (s, 1C) 124.12 (s, 1C) 124.55 (s, 1C) 126.70 (s, 1C) 127.62 (s, 1C) 129.41 (s, 2C) 129.84 (s, 1C) 130.43 (s, 2C) 130.62 (s, 1C) 130.87 (s, 1C) 131.59 (s, 1C) 131.86 (s, 2C) 132.11 (s, 1C) 132.31 (s, 1C) 133.37 (s, 1C) 134.09 (s, 1C) 138.74 (s, 1C) 139.35 (s, 1C) 139.49 (s, 1C) 140.65 (s, 1C) 143.34 (s, 1C) 143.63 (s, 1C) 147.31 (s, 1C) 154.19 (s, 1C) 156.58 (s, 1C) 166.17 (s, 1C); **HRMS (ESI):** [R_t]: 5.38 min, *m/z* for $C_{40}H_{39}N_6O_2$ ([M+H$^+$]), 635.3219 calcd., 635.3128 found.

Synthesis of 6-(4-((4-(6-acrylamido-2-oxo-2,3-dihydro-1H-benzo[d]imidazol-1-yl)piperidin-1-yl)methyl)phenyl)-N-(3-aminophenyl)-5-phenylnicotinamide (**18**).
Chloropyridine (**S30**, 20.0 mg, 0.06 mmol, 1.0 eq.), (4-((4-(6-acrylamido-2-oxo-2,3-dihydro-1*H*-benzo[*d*]imidazol-1-yl)piperidin-1-yl)methyl)phenyl)boronic acid (28.2 mg, 0.06 mmol, 1.0 eq.), K_2CO_3 (15.5 mg, 0.12 mmol, 2.0 eq.) and tetrakis(triphenylphosphine)palladium(0) (6.9 mg, 0.006 mol, 0.1 eq.) were used following common procedure E and yielded the final compound as a solid (17.0 mg, 0.03 mmol, 42 %). **^{1}H-NMR** (600 M Hz, DMSO-d_6) δ ppm 1.65 (d, J = 9.90 Hz, 2H) 2.09 (t, J = 11.10 Hz, 2H) 2.30 (dd, J = 12.47, 3.48 Hz, 2H) 2.95 (d, J = 11.19 Hz, 2H) 3.53 (s, 2H) 4.11 (s, 1H) 5.12 (s, 2H) 5.71 - 5.78 (m, 1H) 6.21 - 6.27 (m, 1H) 6.32 - 6.36 (m, 1H) 6.41 (dd, J = 16.96, 10.18 Hz, 1H) 6.88 (d, J = 8.99 Hz, 1H) 6.91 (d, J = 8.44 Hz, 1H) 6.96 - 7.02 (m, 1H) 7.11 (t, J = 1.93 Hz, 1H) 7.24 - 7.31 (m, 5H) 7.31 - 7.39 (m, 5H) 7.70 (d, J = 1.65 Hz, 1H) 8.31 (d, J =2.20 Hz, 1H) 9.13 (d, J = 2.02 Hz, 1H) 10.07 (s, 1H) 10.20 (s, 1H) 10.80 (s, 1H); **^{13}C-NMR** (151 M Hz, DMSO-d_6) δ ppm 25.43 (s, 2C) 29.15 (s, 1C) 50.60 (s, 1C) 53.12 (s, 2C) 61.98 (s, 1C) 101.38 (s, 1C) 104.66 (s, 1C) 106.54 (s, 1C) 108.76 (s, 1C) 109.11 (s, 1C) 110.51 (s, 1C) 112.58 (s, 1C) 124.96 (s, 1C) 126.82 (s, 1C) 128.08 (s, 1C) 128.81 (s, 2C) 128.96 (s, 2C) 129.36 (s, 2C) 129.45 (s, 1C) 129.54 (s, 1C) 129.89 (s, 1C) 130.11 (s, 2C) 132.52 (s, 1C) 133.12 (s, 1C) 135.23 (s, 1C) 138.02 (s, 1C) 138.49 (s, 1C) 138.98 (s, 1C) 139.53 (s, 1C) 139.90 (s, 1C) 147.95 (s, 1C) 149.50 (s, 1C) 154.47 (s, 1C) 158.69 (s, 1C) 163.24 (s, 1C) 163.80 (s, 1C); **HRMS (ESI):** [R_t]: 5.68 min, *m/z* for $C_{40}H_{38}N_7O_3$ ([M+H$^+$]), 664.3030 calcd., 664.3030 found.

Synthesis of N-(3-(1-(4-(5-(4-aminobenzyl)-3-phenylpyridin-2-yl)benzyl)piperidin-4-yl)-2-oxo-2,3-dihydro-1H-benzo[d]imidazol-5-yl)acrylamide (**19**).
Chloropyridine (**S19**, 20.0 mg, 0.07 mmol, 1.0 eq.), (4-((4-(6-acrylamido-2-oxo-2,3-dihydro-1*H*-benzo[*d*]imidazol-1-yl)piperidin-1-yl)methyl)phenyl)boronic acid (29.4 mg, 0.07 mmol, 1.0 eq.), K_2CO_3 (18.7 mg, 0.14 mmol, 2.0 eq.) and tetrakis(triphenylphosphine)palladium(0) (8.0 mg, 0.007 mol, 0.1 eq.) were used following common procedure E and yielded the final compound as a solid (9.4 mg, 0.01 mmol, 21 %). **^{1}H-NMR** (700 M Hz, DMSO-d_6) δ ppm 1.95 (d, J = 12.26 Hz, 2 H) 2.61 (d, J = 14.09 Hz, 2 H) 3.15 - 3.24 (m, 2 H) 3.45 (d, J = 11.51 Hz, 2 H) 4.07 (s, 2 H) 4.27 - 4.32 (m, 2 H) 4.35 - 4.39 (m, 1 H) 4.51 (s, 2 H) 5.74 (d, J = 10.22 Hz, 1 H) 6.23 (d, J = 17.21 Hz, 1 H) 6.43 (dd, J = 16.83, 10.16 Hz, 1 H) 6.95 (s, 2 H) 7.10 - 7.17 (m, 4 H) 7.30 (s, 2 H) 7.35 - 7.41 (m, 5 H) 7.71 (s, 1 H) 7.83 (s, 1 H) 8.61 (s, 1 H) 9.72 (s, 1 H) 10.08 (s, 1 H) 10.88 (s, 1 H); **^{13}C-NMR** (126 M Hz, DMSO-d_6) δ ppm 25.89 (s, 2 C) 36.88 (s, 1C) 47.55 (s, 1C) 51.06 (s, 2 C) 58.90 (s, 1C) 101.39 (s, 1C) 108.86 (s, 1C) 113.27 (s, 1C) 115.34 (s, 1C) 117.02 (s, 1C) 124.63 (s, 1C) 126.41 (s, 1C) 127.47 (s, 1C) 128.42 (s, 2 C) 128.88 (s, 1C) 129.01 (s, 1C) 129.33 (s, 2

C) 129.93 (s, 3 C) 130.04 (s, 2 C) 130.79 (s, 2 C) 131.89 (s, 1C) 132.38 (s, 1C) 135.42 (s, 1C) 136.03 (s, 1C) 138.74 (s, 1C) 139.11 (s, 1C) 140.99 (s, 1C) 148.25 (s, 2 C) 153.23 (s, 1C) 153.94 (s, 1C) 162.83 (s, 1C); **HRMS (ESI):** [R_t]: 5.71 min, *m/z* for $C_{40}H_{38}N_6O_2$ ([M+H$^+$]), 635.3129 calcd., 635.3128 found.

Synthesis of N-(3-(1-(4-(5-((6-aminopyridin-3-yl)methyl)-3-phenylpyridin-2-yl)benzyl)piperidin-4-yl)-2-oxo-2,3-dihydro-1H-benzo[d]imidazol-5-yl)acrylamide (**20**).
Chloropyridine (**S16**, 20.0 mg, 0.06 mmol, 1.0 eq.), (4-((4-(6-acrylamido-2-oxo-2,3-dihydro-1*H*-benzo[*d*]imidazol-1-yl)piperidin-1-yl)methyl)phenyl)boronic acid (28.2 mg, 0.06 mmol, 1.0 eq.), K_2CO_3 (15.5 mg, 0.12 mmol, 2.0 eq.) and tetrakis(triphenylphosphine)palladium(0) (6.9 mg, 0.006 mol, 0.1 eq.) were used following common procedure E and yielded the final compound as a solid (14.2 mg, 0.02 mmol, 37%). **^{1}H-NMR** (500 M Hz, MeOD-*d*$_4$) δ ppm 2.06 - 2.13 (m, 2H) 2.71 - 2.84 (m, 2H) 3.19 - 3.29 (m, 2H) 3.58 - 3.68 (m, 2H) 4.12 (s, 2H) 4.39 (s, 2H) 4.48 - 4.56 (m, 1H) 5.76 - 5.81 (m, 1H) 6.33 - 6.40 (m, 1H) 6.44 (d, *J* = 9.77 Hz, 1H) 6.96 - 7.06 (m, 3H) 7.16 - 7.21 (m, 2H) 7.30 (dd, *J* = 5.11, 1.91 Hz, 3H) 7.45 - 7.54 (m, 4H) 7.86 (d, *J* = 1.37 Hz, 1H) 7.91 - 7.94 (m, 2H) 7.97 (d, *J* = 1.98 Hz, 1H) 8.64 (d, *J* = 2.14 Hz, 1H); **^{13}C-NMR** (176 M Hz, DMSO-*d*$_6$) δ ppm 22.87 (s, 2C) 27.21 (s, 1C) 33.04 (s, 1C) 49.72 (s, 1C) 51.66 (s, 2C) 60.78 (s, 1C) 101.76 (s, 1C) 107.97 (s, 1C) 108.42 (s, 1C) 113.09 (s, 1C) 123.42 (s, 1C) 125.26 (s, 1C) 126.27 (s, 1C) 127.11 (s, 2C) 128.02 (s, 1C) 128.39 (s, 2C) 128.69 (s, 2C) 130.16 (s, 2C) 131.37 (s, 1C) 135.38 (s, 1C) 135.72 (s, 1C) 137.81 - (s, 1C) 138.20 (s, 1C) 138.40 (s, 1C) 145.06 (s, 1C) 146.56 (s, 1C) 153.87 (s, 1C) 154.26 (s, 1C) 157.17 (s, 1C) 163.62 (s, 1C); **HRMS (ESI):** [R_t]: 5.68 min, *m/z* for $C_{39}H_{38}N_7O_2$ ([M+H$^+$]), 636.3081Calcd., 636.3078 found.

Synthesis of N-(2-oxo-3-(1-(4-(3-phenyl-5-(pyridin-3-ylmethyl)pyridin-2-yl)benzyl)piperidin-4-yl)-2,3-dihydro-1H-benzo[d]imidazol-5-yl)acrylamide (**21**).
Chloropyridine (**S21**, 20.0 mg, 0.06 mmol, 1.0 eq.), (4-((4-(6-acrylamido-2-oxo-2,3-dihydro-1*H*-benzo[*d*]imidazol-1-yl)piperidin-1-yl)methyl)phenyl)boronic acid (28.2 mg, 0.06 mmol, 1.0 eq.), K_2CO_3 (15.5 mg, 0.12 mmol, 2.0 eq.) and tetrakis(triphenylphosphine)palladium(0) (6.9 mg, 0.006 mol, 0.1 eq.) were used following common procedure E and yielded the final compound as a solid (15.2 mg, 0.02 mmol, 40 %). **^{1}H-NMR** (600 M Hz, DMSO-*d*$_6$) δ ppm 1.92 - 1.98 (m, 2H) 2.57 - 2.65 (m, 2H) 3.16 - 3.24 (m, 2H) 3.41 - 3.48 (m, 2H) 4.27 (s, 2H) 4.30 (s, 3H) 4.34 - 4.40 (m, 2H) 5.71 - 5.76 (m, 1H) 6.21 - 6.26 (m, 1H) 6.39 - 6.47 (m, 1H) 6.95 (s, 2H) 7.04 (s, 1H) 7.13 (s, 1H) 7.14 - 7.19 (m, 3H) 7.21 (s, 1H) 7.28 - 7.34 (m, 5H) 7.35 - 7.38 (m, 3H) 7.39 - 7.44 (m, 3H) 7.79 - 7.82 (m, 2H) 7.83 - 7.87 (m, 4H) 8.32 - 8.37 (m, 1H) 8.70 (d, *J* = 2.20 Hz, 4H) 8.90 (d, *J* = 1.47 Hz, 1H) 9.73 - 9.78 (m, 1H) 10.09 (s, 1H) 10.89 (s, 1H); **^{13}C-NMR** (151 M Hz, DMSO-*d*$_6$) δ ppm 26.37 (s, 2C) 34.64 (s, 1C) 48.02 (s, 1C) 51.55 (s, 2C) 59.36 (s, 1C) 101.88 (s, 1C) 109.36 (s, 1C) 113.40 (s, 1C) 113.76 (s, 1C) 115.34 (s, 1C) 117.28 (s, 1C) 119.21 (s, 1C) 125.12 (s, 1C) 126.47 (s, 1C) 126.91 (s, 1C) 128.04 (s, 1C) 128.90 (s, 2C) 129.50 (s, 1C) 129.88 (s, 2C) 130.53 (s, 1C) 131.32 (s, 1C) 132.37 (s, 1C) 132.87 (s, 1C) 134.84 (s, 1C) 136.10 (s, 1C) 139.14 (s, 1C) 139.40 (s, 1C) 139.64 (s, 1C) 141.31 (s, 1C) 143.48 (s, 1C) 145.13 (s, 1C) 148.85 (s, 1C) 154.19 (s, 1C) 154.43 (s, 1C) 163.32 (s, 1C); **HRMS (ESI):**, *m/z* for $C_{39}H_{37}N_6O_2$ ([M+H$^+$]), 621.2972 calcd., 621.2962 found.

Synthesis of 6-(4-((4-(6-acrylamido-2-oxo-2,3-dihydro-1H-benzo[d]imidazol-1-yl)piperidin-1-yl)methyl)phenyl)-5-phenyl-N-(pyridin-3-yl)nicotinamide (**22**).
Chloropyridine (**S28**, 20.0 mg, 0.06 mmol, 1.0 eq.), (4-((4-(6-acrylamido-2-oxo-2,3-dihydro-1*H*-benzo[*d*]imidazol-1-yl)piperidin-1-yl)methyl)phenyl)boronic acid (28.2 mg, 0.06 mmol, 1.0 eq.), K_2CO_3 (15.5 mg, 0.12 mmol, 2.0 eq.) and tetrakis(triphenylphosphine)palladium(0) (6.9 mg, 0.006 mol, 0.1 eq.) were used following common procedure E and yielded the final compound as a solid (13.4 mg, 0.02 mmol, 34 %). **^{1}H-NMR** (500 M Hz, DMSO-*d*$_6$) δ ppm 1.61 - 1.70 (m, 2H) 1.91 (s, 3H) 2.04 - 2.15 (m, 2H) 2.23 - 2.36 (m, 2H) 2.91 - 3.01 (m, 2H) 3.54 (s, 2H) 4.06 - 4.17 (m, 1H) 5.66 - 5.78 (m, 1H) 6.26 (s, 1H) 6.35 - 6.51 (m, 1H) 6.92 (d, *J* = 8.39 Hz, 1H) 7.23 - 7.41 (m, 6H) 7.41 - 7.47 (m, 1H) 7.70 (d, *J* = 1.37 Hz, 1H) 8.17 - 8.24 (m, 1H) 8.35 (dd, *J* = 4.65, 1.45 Hz, 1H) 8.38 (d, *J* = 2.29 Hz, 1H) 8.95 (d, *J* = 2.44 Hz, 1H) 9.19 (d, *J* = 2.14 Hz, 1H) 10.09 (s, 1H) 10.70 (s, 1H) 10.82 (s, 1H) 12.00 - 12.20 (m, 1H); **^{13}C-NMR** (126 M Hz, DMSO-*d*$_6$) δ ppm 19.05 (s, 1C) 29.14 (s, 2C) 50.56 (s, 1C) 53.12 (s, 2C) 56.49 (s, 1C) 61.96 (s, 1C) 101.37 (s, 1C) 109.13 (s, 1C) 112.58 (s, 1C) 124.13 (s, 1C) 124.95 (s, 1C) 126.87 (s, 1C) 127.88 (s, 2C) 128.18 (s, 1C) 128.68 (s, 1C) 128.84 (s, 1C) 129.02 (s, 2C) 129.43 (s, 2C) 129.89 (s, 1C) 130.15 (s, 1C) 132.50 (s, 1C) 133.11 (s, 1C) 135.36 (s, 1C) 136.02 (s, 1C) 138.18 (s, 1C) 139.41 (s, 1C) 142.44 (s, 2C) 145.38 (s, 1C) 148.08 (s, 1C) 154.46 (s, 1C) 159.15 (s, 1C) 163.23 (s, 1C) 164.55 (s, 1C); **HRMS (ESI):** [R_t]: 5.63 min, *m/z* for $C_{39}H_{36}N_7O_3$ ([M+H$^+$]), 650.2874 calcd., 650.2866 found.

Synthesis of 6-(4-((4-(6-acrylamido-2-oxo-2,3-dihydro-1H-benzo[d]imidazol-1-yl)piperidin-1-yl)methyl)phenyl)-5-phenyl-N-(pyridin-2-yl)nicotinamide (**23**).
Chloropyridine (**S29**, 20.0 mg, 0.06 mmol, 1.0 eq.), (4-((4-(6-acrylamido-2-oxo-2,3-dihydro-1*H*-benzo[*d*]imidazol-1-yl)piperidin-1-yl)methyl)phenyl)boronic acid (28.2 mg, 0.06 mmol, 1.0 eq.), K_2CO_3 (15.5 mg, 0.12 mmol, 2.0 eq.) and tetrakis(triphenylphosphine)palladium(0) (6.9 mg, 0.006 mol, 0.1 eq.) were used following common procedure E and yielded the final compound as a solid (23.5 mg, 0.04 mmol, 60 %). **^{1}H-NMR** (600 M Hz, DMSO-*d*$_6$) δ ppm 1.96 (d, *J* = 12.29 Hz, 2H) 2.56 - 2.69 (m, 2H) 3.21 (d, *J* = 12.29 Hz, 2H) 3.47 (d, *J* = 11.55 Hz, 2H) 4.34 (d, *J* = 4.22 Hz, 2H) 4.37 - 4.45 (m, 1H) 5.72 - 5.77 (m, 1H) 6.24 (dd, *J* = 16.96, 1.93 Hz, 1H) 6.43 (dd, *J* = 16.96, 10.18 Hz, 1H) 6.95 (s, 2H) 7.21 (ddd, *J* = 7.29, 4.91, 1.01 Hz, 1H) 7.29 - 7.40 (m, 5H) 7.42 - 7.48 (m, 4H) 7.84 (s, 1H) 7.89 (ddd, *J* = 8.34, 7.43, 2.02 Hz, 1H) 8.24 (d, *J* = 8.44 Hz, 1H) 8.42 (ddd, *J* = 4.91, 1.88, 0.92 Hz, 1H) 8.48 (d, *J* = 2.20 Hz, 1H) 9.22 (d, *J* = 2.02 Hz, 1H) 9.72 - 9.81 (m, 1H) 10.09 (s, 1H) 10.89 (s, 1H) 11.27 (s, 1H); **^{13}C-NMR** (151 M Hz, DMSO-*d*$_6$) δ ppm 25.92 (s, 2C) 47.55 (s, 1C) 51.10 (s, 2C) 58.86 (s, 1C) 101.41 (s, 1C) 108.87 (s, 1C) 113.29 (s, 1C) 114.73 (s, 1C) 114.94 (s, 2C) 116.88 (s, 1C) 120.15 (s, 1C) 124.65 (s, 1C) 126.44 (s, 1C) 127.77 (s, 1C) 128.50 (s, 2C) 129.04 (s, 1C) 129.52 (s, 2C) 129.59 (s, 1C) 130.17 (s, 1C) 130.96 (s, 2C) 131.90 (s, 1C) 132.39 (s, 1C) 134.90 (s, 1C) 138.23 (s, 1C) 138.40 (s, 1C)

138.57 (s, 1C) 140.61 (s, 1C) 147.83 (s, 1C) 147.95 (s, 1C) 151.95 (s, 1C) 153.96 (s, 1C) 157.92 (s, 1C) 162.84 (s, 1C) 164.25 (s, 1C); **HRMS (ESI):** [R_t]: 6.38 min, *m/z* for $C_{39}H_{36}N_7O_3$ ([$M+H^+$]), 650.2874 calcd., 650.2868 found.

Supplementary References

1. Huang, X. et al. Crystal Structure of an Inactive Akt2 Kinase Domain. *Structure* **11**, 21-30 (2003).
2. Quambusch, L. et al. Covalent-Allosteric Inhibitors to Achieve Akt Isoform-Selectivity. *Angew Chem Int Ed Engl* **58**, 18823-18829 (2019).
3. Baker, N.A., Sept, D., Joseph, S., Holst, M.J. & McCammon, J.A. Electrostatics of nanosystems: application to microtubules and the ribosome. *Proceedings of the National Academy of Sciences* **98**, 10037-10041 (2001).
4. Zhai, X., Ward, R.A., Doig, P. & Argyrou, A. Insight into the Therapeutic Selectivity of the Irreversible EGFR Tyrosine Kinase Inhibitor Osimertinib through Enzyme Kinetic Studies. *Biochemistry* **59**, 1428-1441 (2020).
