# Supplementary material for: Cellular model system to dissect the isoform-selectivity of Akt inhibitors
Source: Nat Commun. 2021 Sep 6;12:5297. doi: 10.1038/s41467-021-25512-8 (PMC8421423; doi:10.1038/s41467-021-25512-8)
Supplement: Supplementary file 3 — Reporting Summary [file 41467_2021_25512_MOESM3_ESM.pdf]

nature research

Corresponding author(s): Daniel Rauh

Last updated by author(s): Aug 9, 2021

Reporting Summary

Nature Research wishes to improve the reproducibility of the work that we publish. This form provides structure for consistency and transparency in reporting. For further information on Nature Research policies, see our Editorial Policies and the Editorial Policy Checklist.

Statistics

For all statistical analyses, confirm that the following items are present in the figure legend, table legend, main text, or Methods section.

n/a	Confirmed	
☐	☒	The exact sample size (*n*) for each experimental group/condition, given as a discrete number and unit of measurement
☐	☒	A statement on whether measurements were taken from distinct samples or whether the same sample was measured repeatedly
☒	☐	The statistical test(s) used AND whether they are one- or two-sided<br>*Only common tests should be described solely by name; describe more complex techniques in the Methods section.*
☒	☐	A description of all covariates tested
☒	☐	A description of any assumptions or corrections, such as tests of normality and adjustment for multiple comparisons
☐	☒	A full description of the statistical parameters including central tendency (e.g. means) or other basic estimates (e.g. regression coefficient) AND variation (e.g. standard deviation) or associated estimates of uncertainty (e.g. confidence intervals)
☒	☐	For null hypothesis testing, the test statistic (e.g. *F*, *t*, *r*) with confidence intervals, effect sizes, degrees of freedom and *P* value noted<br>*Give P values as exact values whenever suitable.*
☒	☐	For Bayesian analysis, information on the choice of priors and Markov chain Monte Carlo settings
☒	☐	For hierarchical and complex designs, identification of the appropriate level for tests and full reporting of outcomes
☒	☐	Estimates of effect sizes (e.g. Cohen's *d*, Pearson's *r*), indicating how they were calculated

Our web collection on statistics for biologists contains articles on many of the points above.

Software and code

Policy information about availability of computer code

Data collection	NMR data collected with: Bruker Avance AV500, AV600, AV700<br>Fluorescent data collected with: Tecan Infinite M1000 Pro, EnVision (PerkinElmer)<br>WB read out: Odyssey CLx (LiCor)<br>MS-data collected: Thermo Fisher Scientific Velos Pro, Finnigan LCQ Advantage, LTQ Orbitrap
Data analysis	All software used in current study were commercially or publicly available and described in the methods section.<br>Data displayed in graphs were generated and analyzed with OriginPro.<br>NMR data was analysed with ACD/Labs 12.01 (Advanced Chemistry Development Inc.)<br>Kinetic data was analyzed with OriginPro 9.7.5 (OriginLab).<br>HTRF and CTG data were analyzed with Quattro Software Suite 11.2.0.3<br>MS data was evaluated with Xcalibur 2.2.48, deconvoluted with MagTran v1.02, MaxQuant v1.6.1.0 or ProMass for Xcalibur v2.8 rev.2<br>Crystal structures were obtained using XDS (VERSION Jan 31, 2020 BUILT=20200131), WinCoot 0.8.9.1, PHASER (integrated in Phenix), Phenix 1.17.1 and PyMOL 2.0.6<br>Homology models were build with SWISS-MODEL homology-modelling server, sequence alignment was performed with Clustal Omega and Ligand models generated with Ligandscout 4.0 (Inte:Ligand).

For manuscripts utilizing custom algorithms or software that are central to the research but not yet described in published literature, software must be made available to editors and reviewers. We strongly encourage code deposition in a community repository (e.g. GitHub). See the Nature Research guidelines for submitting code & software for further information.

Data

Policy information about availability of data

All manuscripts must include a data availability statement. This statement should provide the following information, where applicable:

- Accession codes, unique identifiers, or web links for publicly available datasets
- A list of figures that have associated raw data
- A description of any restrictions on data availability

The data supporting the findings of this study are available in the paper and its Supplementary Information. Source Data are provided with this paper. The crystal structure data generated in this study have been deposited in the PDB database under accession code 7NH4 [http://doi.org/10.2210/pdb7NH4/pdb] and 7NH5 [http://doi.org/10.2210/pdb7NH5/pdb]. Already reported Structures are deposited under following accession codes: 1MRY [http://doi.org/10.2210/pdb1MRY/pdb], 2UVM [http://doi.org/10.2210/pdb2UVM/pdb], 4GV1 [http://doi.org/10.2210/pdb4GV1/pdb], 6HHF [http://doi.org/10.2210/pdb6HHF/pdb], and 6S9X [http://doi.org/10.2210/pdb6S9X/pdb]. Original diffraction data can be accessed on proteindiffraction.org [http://doi.org].

Field-specific reporting

Please select the one below that is the best fit for your research. If you are not sure, read the appropriate sections before making your selection.

☒ Life sciences ☐ Behavioural & social sciences ☐ Ecological, evolutionary & environmental sciences

For a reference copy of the document with all sections, see nature.com/documents/nr-reporting-summary-flat.pdf

Life sciences study design

All studies must disclose on these points even when the disclosure is negative.

Sample size	For HTRF assay the sample size was determined based on the manufacturer's protocol. For cell viability CTG assay the sample size was determined using a concentration series of eight repetitive bisecting concentrations. The optimal cell number for the cell viability assay was determined from the linear growth range of each cell line. For western blot sample preparation the number of seeded cells was based on prior experience and experiments. No statistical method was used to predetermine sample size.
Data exclusions	No data were excluded.
Replication	HTRF CTG assay were succsessfully replicated at least three times with two technical replicates in each experiment. All other experiments were performed succsessfully at least twice to ensure reproducibility. Qualitative and quantitative Western blots were performed once.
Randomization	For in vitro studies, the samples/cells were randomized into different groups prior to treatment.
Blinding	Investigators were not blinded to the experiments as the researchers need to rank and load the samples based on the treatment information. Blinding was not relevant for biochemical in vitro experiments we used manufactured enzymes and known substrates.

Reporting for specific materials, systems and methods

We require information from authors about some types of materials, experimental systems and methods used in many studies. Here, indicate whether each material, system or method listed is relevant to your study. If you are not sure if a list item applies to your research, read the appropriate section before selecting a response.

Materials & experimental systems

n/a	Involved in the study	
☐	☒	Antibodies
☐	☒	Eukaryotic cell lines
☒	☐	Palaeontology and archaeology
☒	☐	Animals and other organisms
☒	☐	Human research participants
☒	☐	Clinical data
☒	☐	Dual use research of concern

Methods

n/a	Involved in the study	
☒	☐	ChIP-seq
☒	☐	Flow cytometry
☒	☐	MRI-based neuroimaging

Antibodies

Antibodies used	Anti-pAkt(Ser473) (CST, cat. no. 4060), anti-tAkt1 (CST, cat. no. 2938), anti-tAkt2 (CST, cat. no. 3063), anti-tAkt3 (CST, cat. No. 3788), anti-pAkt1 (CST, cat. No. 9018), anti-pAkt2 (CST, cat. No. 8599), anti-pAkt3 (Thermo Fisher Scientific, cat. No. PA5-12898), anti-pPRAS40(Thr246) (CST, cat. no. 2997), anti-pS6(Ser235/236) (CST, cat. no. 2317, 1:2000), anti-pERK1/2(Thr202/204) (CST, cat. No. 4370), anti-pFOXO (CST, cat. no. 2599, 1:500), anti-pGSK3β (CST, cat. no. 5558), anti-cPARP/PARP (CST, cat. no. 9542), anti-β-Actin (Sigma, cat. no. A5441, 1:5000), anti-mouse IgG (H+L) (DyLight™ 680 Conjugate) (CST, cat. no. 5470, 1:15000), anti-rabbit IgG (H+L)

(DyLight™ 800 4X PEG Conjugate) (CST, cat. no. 5151, 1:15000). Antibodies were diluted in a ratio of 1:1000. Deviating dilutions are depicted behind the corresponding antibody.

Validation

Anti-pAkt(Ser473) (CST, cat. no. 4060):
Specificity / Sensitivity: Phospho-Akt (Ser473) (D9E) XP® Rabbit mAb detects endogenous levels of Akt only when phosphorylated at Ser473.
Species Reactivity: Human, Mouse, Rat, Hamster, Monkey, D. melanogaster, Zebrafish, Bovine
Species predicted to react based on 100% sequence homology: Chicken, Xenopus, Dog, Pig

anti-tAkt1 (CST, cat. no. 2938):
Specificity / Sensitivity: Akt1 (C73H10) Rabbit mAb detects endogenous levels of total Akt1 protein. This antibody does not cross-react with Akt2 or Akt3.
Species Reactivity: Human, Mouse, Rat, Monkey

anti-tAkt2 (CST, cat. no. 3063)
Specificity / Sensitivity: Akt2 (D6G4) Rabbit mAb detects endogenous levels of total Akt2 protein. It does not cross-react with Akt1 or Akt3.
Species Reactivity: Human, Mouse, Rat, Monkey

anti-tAkt3 (CST, cat. No. 3788):
Specificity / Sensitivity: Akt3 (62A8) Rabbit mAb detectes endogenous levels of total Akt3 protein. The antibody does not cross-react with recombinant Akt1 or Akt2.
Species Reactivity: Human, Mouse, Rat

anti-pAkt1 (CST, cat. No. 9018):
Specificity / Sensitivity: Phospho-Akt1 (Ser473) (D7F10) XP® Rabbit mAb (Akt1 Specific) recognizes endogenous levels of Akt1 protein only when phosphorylated at Ser473. It does not detect Akt2 protein when phosphorylated at Ser474.
Species Reactivity: Human, Mouse, Rat

anti-pAkt2 (CST, cat. No. 8599):
Specificity / Sensitivity: Phospho-Akt2 (Ser474) (D3H2) Rabbit mAb (Akt2 Specific) recognizes endogenous levels of Akt2 protein only when phosphorylated at Ser474. This antibody does not cross-react with Akt1 protein when phosphorylated at Ser473 or with Akt3 protein when phosphorylated at Ser472.
Species Reactivity: Human, Mouse, Rat

anti-pAkt3 (Thermo Fisher Scientific, cat. No. PA5-12898):
This antibody is predicted to react with mouse and rat based on sequence homology.

anti-pPRAS40(Thr246) (CST, cat. no. 2997):
Specificity / Sensitivity: Phospho-PRAS40 (Thr246) (C77D7) Rabbit mAb detects endogenous levels of PRAS40 protein only when phosphorylated at Thr246.
Species Reactivity: Human, Mouse, Rat, Monkey

anti-pS6(Ser235/236) (CST, cat. no. 2317):
Specificity / Sensitivity: S6 Ribosomal Protein (54D2) Mouse mAb detects endogenous levels of total S6 ribosomal protein independent of phosphorylation.
Species Reactivity: Human, Mouse, Rat, Monkey, D. melanogaster

anti-pERK1/2(Thr202/204) (CST, cat. No. 4370)
Specificity / Sensitivity: Phospho-p44/42 MAPK (Erk1/2) (Thr202/Tyr204) (D13.14.4E) XP® Rabbit mAb detects endogenous levels of p44 and p42 MAP Kinase (Erk1 and Erk2) when dually phosphorylated at Thr202 and Tyr204 of Erk1 (Thr185 and Tyr187 of Erk2), and singly phosphorylated at Thr202. This antibody does not cross-react with the corresponding phosphorylated residues of either JNK/SAPK or p38 MAP kinases.
Species Reactivity: Human, Mouse, Rat, Hamster, Monkey, Mink, D. melanogaster, Zebrafish, Bovine, Dog, Pig, S. cerevisiae
Species predicted to react based on 100% sequence homology: Chicken, C. elegans

anti-pFOXO (CST, cat. no. 2599):
Specificity / Sensitivity: Phospho-FoxO1 (Thr24)/FoxO3a (Thr32)/Fox04 (Thr28) (4G6) Rabbit mAb detects endogenous levels of FoxO1 when phosphorylated at Thr24, of FoxO3a when phosphorylated at Thr32 or FoxO4 when phosphorylated at Thr28.
Species Reactivity: Human, Mouse, Monkey

anti-pGSK3β (CST, cat. no. 5558):
Specificity / Sensitivity: Phospho-GSK-3β (Ser9) (D85E12) XP® Rabbit mAb detects endogenous levels of GSK-3β only when phosphorylated at Ser9. This antibody reacts with denatured components of bovine serum, including BSA.
Species Reactivity: Human, Mouse, Rat, Hamster

anti-cPARP/PARP (CST, cat. no. 9542):
Specificity / Sensitivity: PARP Antibody detects endogenous levels of full length PARP1 (116 kDa), as well as the large fragment (89 kDa) of PARP1 resulting from caspase cleavage. The antibody does not cross-react with related proteins or other PARP isoforms.
Species Reactivity: Human, Mouse, Rat, Monkey

anti-β-Actin (Sigma, cat. no. A5441):
species reactivity: pig, Hirudo medicinalis, bovine, rat, canine, feline, human, rabbit, carp, mouse, guinea pig, chicken, sheep

anti-mouse IgG (H+L) (DyLight™ 680 Conjugate) (CST, cat. no. 5470):
Specificity / Sensitivity: Anti-mouse IgG (H+L) (DyLight™ 680 Conjugate) reacts with heavy and light chain of most mouse immunoglobulins. No cross-reactivity to other serum proteins has been detected. This antibody may cross-react with immunoglobulins from other species.

anti-rabbit IgG (H+L) (DyLight™ 800 4X PEG Conjugate) (CST, cat. no. 5151):
Specificity / Sensitivity: Anti-rabbit IgG (H+L) (DyLight™ 800 4X PEG Conjugate) reacts with heavy and light chain of most rabbit immunoglobulins. No cross-reactivity to other serum proteins has been detected. This antibody may cross-react with immunoglobulins from other species

Antibody validation Cell Signaling Technologies:
CST adhere to the Hallmarks of Antibody Validation™, six complementary strategies that can be used to determine the functionality, specificity, and sensitivity of an antibody in any given assay. CST adapted the work by Uhlen, et. al., ("A Proposal for Validation of Antibodies." Nature Methods (2016)) to build the Hallmarks of Antibody Validation.
Binary Model: Antibody signal is measured in model systems with known presence/absence of target signal. Includes wild-type vs. genetic knockout, targeted induction or silencing.

Ranged Expression: Antibody signal strength is measured in cell lines or tissues representing a known continuum of target expression levels. Includes siRNA and heterozygous knockout assays.

Orthogonal Data: Antibody signal is correlated to target expression in model systems measured using antibody independent assays. Includes mass spectrometry and in situ hybridization.

Multiple Antibodies: Antibody signal is compared to the signal observed using antibodies targeting nonoverlapping epitopes of the target. Includes IP, ChIP, and ChIP-seq.

Heterologous Expression: Antibody signal is evaluated in cell lines following heterologous expression of native (or mutated) target protein.

Complementary Assays: Antibody specificity may be validated using complementary assays. Includes competitive ELISA, peptide dot blots, peptide blocking, or protein arrays.

Antibody validation Sigma Aldrich:
Antibody Standard Validation: Species validation, Storage and Handling, Application validation, Lot validation, Quality control
Antibody validation Thermo:
Invitrogen antibodies are currently undergoing a rigorous 2-part testing approach
Part 1—Target specificity verification
This helps ensure the antibody will bind to the correct target. Our antibodies are being tested using at least 1 of the following methods to ensure proper functionality in researcher's experiments. Click on each testing method below for detailed testing strategies, workflow examples and data figure legends.
Knockout—expression testing using CRISPR-Cas9 cell models
Knockdown—expression testing using RNAi to knockdown gene of interest
Independent antibody verification (IAV)—measurement of target expression is performed using two differentially raised antibodies recognizing the same protein target
Cell treatment—detecting downstream events following cell treatment
Relative expression—using naturally occurring variable expression to confirm specificity
Neutralization—functional blocking of protein activity by antibody binding
Peptide array—using arrays to test reactivity against known protein modifications
SNAP-ChIP™—using SNAP ChIP to test reactivity against known protein modifications
Immunoprecipitation-Mass Spectrometry (IP-MS)—testing using immunoprecipitation followed by mass spectrometry to identify antibody targets
Part 2—Functional application validation
These tests help ensure the antibody works in a particular application(s) of interest, which may include (but are not limited to):
Western blotting
Flow cytometry
ChIP
Immunofluorescence imaging
Immunohistochemistry
Most antibodies were developed with specific applications in mind. Testing that an antibody generates acceptable results in a specific application is the second part of confirming antibody performance.
Advanced Verification
Thermo Fisher Scientific is committed to adopting validation standards for our Invitrogen antibody portfolio. The Advanced Verification badge is applied to products that have passed application and specificity testing. This badge can be found in the search results and at the top of the product specific webpages. Data supporting the Advanced Verification badges can be found in product specific data galleries. To learn more please visit Invitrogen Antibody Validation

Eukaryotic cell lines

Policy information about cell lines

Cell line source(s)	HEK293T cells were a gift from Dr. Daniel Summerer (Technical University Dortmund) (ATCC; Cat# CRL-3216). Ba/F3 cells were obtained from Dr. Martin Sos (University of Cologne). PANC1 cells were a gift from Dr. Jens Siveke (University Hospital, Essen) (ATCC; Cat# CRL-1469). BaF3 myr-Akt1/2/3 were generated using retroviral infection.
Authentication	Evaluation of BaF3 myr-Akt1/2/3 by DNA-seq. No additional authentification of parental BaF3 and HEK293T were performed. Cell line authentication of PANC1 was performed. For cell line authentication we used the Cell Line Authenticity Basic Service from Eurofins Genomics (genotyping according to ANSI/ATCC standard ASN-0002). Cell Line Authentication Service for human cell lines is performed using Applied BiosystemsTM AmpFLSTRTM IdentifilerTM Plus PCR Amplification Kit with 16 markers (CSF1PO, D2S1338, D3S1358, D5S818, D7S820, D8S1179, D13S317, D16S539, D18S51, D19S433, D21S11, FGA, TH01, TPOX, vWA, and the gender marker Amelogenin) for high-resolution discrimination and includes all relevant markers of ANSI/ATCC ASN0002-2011. Authentication of human cell lines: Standardization of STR profiling.
Mycoplasma contamination	Cell lines were not tested for mycoplasma contamination.
Commonly misidentified lines (See ICLAC register)	No commonly misidentified lines are included.
